# Organocatalytic Microfluidic Double‐Layer Capacitors

**DOI:** 10.1002/anie.202517078

**Published:** 2025-09-21

**Authors:** Shen‐Yi Guo, Miguel Paraja, Augustina Jozeliūnaitė, Manuel Gallardo‐Villagrán, Qing‐Xia Zhang, Alenka Marsalek, Naomi Sakai, Stefan Matile

**Affiliations:** ^1^ Department of Organic Chemistry University of Geneva Geneva Switzerland; ^2^ National Centre of Competence in Research (NCCR) Molecular Systems Engineering Basel Switzerland

**Keywords:** Arginine, Electrical double layer, Electric‐field catalysis, Enamine chemistry, Flow chemistry, Organocatalysis, Polyarginine

## Abstract

Ideas to use external electric fields to enable, accelerate and direct the movement of electrons during chemical reactions are not new. Theory and experiments under special conditions predict that electric‐field catalysis (EFC) from externally applied fields could change the way we make molecules. The challenge is the incompatibility with organic synthesis under scalable bulk conditions. Access to applied electric fields (AEFs) > 1 V nm^−1^, predicted as necessary for direct transition‐state stabilization, is not possible even with electromicrofluidic systems, where the distance between the plate electrodes is minimized. Therefore, we decided to shift our attention from the applied fields to their consequences. We consider electrical double layers (EDLs) that form within a few nanometers from the plate electrodes as engineerable supramolecular electrodes. Applying lessons from cell‐penetrating peptides (CPPs), we report supramolecular electrodes with effective electric fields (EEFs) that exceed applied fields by more than five million. According to a proline‐catalyzed aldol condensation installed as benchmark reaction, those engineered from polyarginine and pyrenebutyrate are most active for EFC, exactly as in cellular uptake. With the best supramolecular electrodes, EFC triples the yield of one of the most optimized reactions in organocatalysis. New methods to access scalable EFC open up broad perspectives in organic synthesis and beyond.

## Graphical abstract

Highest expectations from catalysis with external electric fields, from organic synthesis to sustainable industrial production and the origin of life, cannot be explored because of the incompatibility with scalable bulk conditions. This challenge is addressed with bioinspired organocatalytic electrical double‐layer architectures as supramolecular electrodes in microfluidic capacitors. The result is an enabling method for scalable electric‐field catalysis.

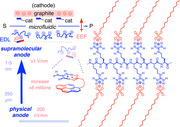

In every chemical reaction, electrons move from one place to another, within the same molecule or between different molecules. Theoretical studies have predicted that externally applied electric fields (AEFs) could control these movements (Figure 1a).^[^ ^1^, ^2^, ^3^, ^4^, ^5^ ^]^ In its simplest form, electric‐field catalysis (EFC) operates by stabilizing large transition state dipoles (Figure 1b). Advanced EFC concepts include dipole reorientation in fixed substrates and more sophisticated ways to control the flow of electrons during a reaction. It is also because of its generality that the possible role of EFC to enable the reactions related to the origin of life attracts increasing attention, particularly considering the preference for phosphates with large transition‐state dipoles.^[^ ^3^, ^6^, ^7^ ^]^ Today, theory and model systems^[^ ^8^, ^9^ ^]^ support that local electric fields in enzymes generally account for the efficiency of biocatalysis near diffusion control.^[^ ^4^, ^5^, ^7^, ^9^, ^10^, ^11^ ^]^ The same generality has led to predictions that EFC could fundamentally transform methods in organic synthesis, which in turn could contribute significantly to sustainable industrial production.^[^ ^1^, ^2^ ^]^

**Figure 1:**
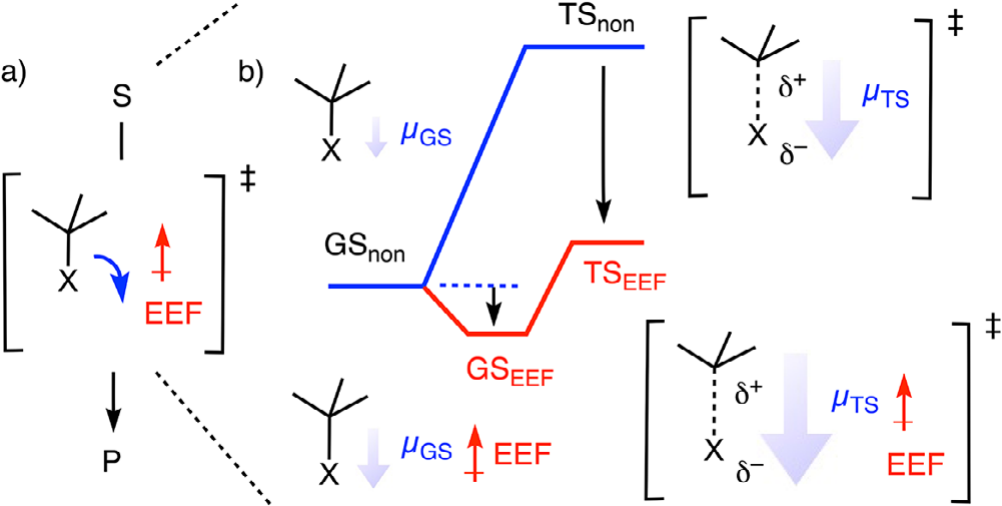
a) Effective electric fields (EEFs) are expected to enable, accelerate, and direct the flow of electrons during a reaction from substrate S to product P. b) For a reaction moving from a non‐stabilized ground state GS_non_ to a rate‐limiting transition state TS_non_, EFC stabilizes the larger transition‐state dipoles *µ*_TS_ in TS_EEF_ more than the smaller ground‐state dipoles *µ*_GS_ in GS_EEF_.

For applications from organic synthesis to sustainable industrial production and studies on the origin of life, the big central challenge with EFC using externally AEFs is its incompatibility with organic chemistry under scalable bulk conditions. Because of this limitation, pioneering EFC studies have focused mostly on special conditions, like water droplets,^[^ ^3^, ^6^, ^12^ ^]^ bubbles,^[^ ^13^ ^]^ coacervates,^[^ ^14^ ^]^ STM tips,^[^ ^15^, ^16^ ^]^ or more specialized house‐made devices.^[^ ^2^, ^17^, ^18^, ^19^ ^]^ To access scalable bulk conditions, we have recently reported EFC on carbon nanotubes in microfluidic electrochemical reactors.^[^ ^20^ ^]^ One reason reactions in microfluidic reactors attract much attention is their intrinsic scalability. Electromicrofluidic reactors have thus been developed to facilitate the electron‐transfer‐based redox chemistry even in large‐scale industrial production.^[^ ^21^, ^22^, ^23^ ^]^ However, used as capacitors, we felt they could also be ideal to realize scalable supramolecular EFC under conditions where electroorganic redox chemistry does not occur.

In the electromicrofluidic reactors used for this study, 5 x 5 cm^2^ electrodes are separated by a thin foil with the flow channel (Figure 2). The thickness of this foil, usually *d* = 250 µm, determines the distance between the plate electrodes. At *V* = 2.5 V, this gives an AEF = *V*/*d* = 10 µV nm^−1^. Even if we maximize the voltage to *V* = 500 V and minimize the electrode separation to *d* = 50 µm, the resulting AEF = 0.01 V nm^−1^ would remain below the >1 V nm^−1^ estimated as necessary for direct impact on reactions (Figure 1b).^[^ ^1^, ^15^, ^24^ ^]^ To overcome this problem, we here propose to shift attention from metal plates to supramolecular electrodes. Using proline‐catalyzed Robinson annulation as a benchmark reaction,^[^ ^8^, ^25^, ^26^, ^27^, ^28^, ^29^ ^]^ we apply lessons from the cellular uptake of cell‐penetrating peptides (CPPs)^[^ ^30^ ^]^ to construct organocatalytic electrical double layers (EDLs).

**Figure 2:**
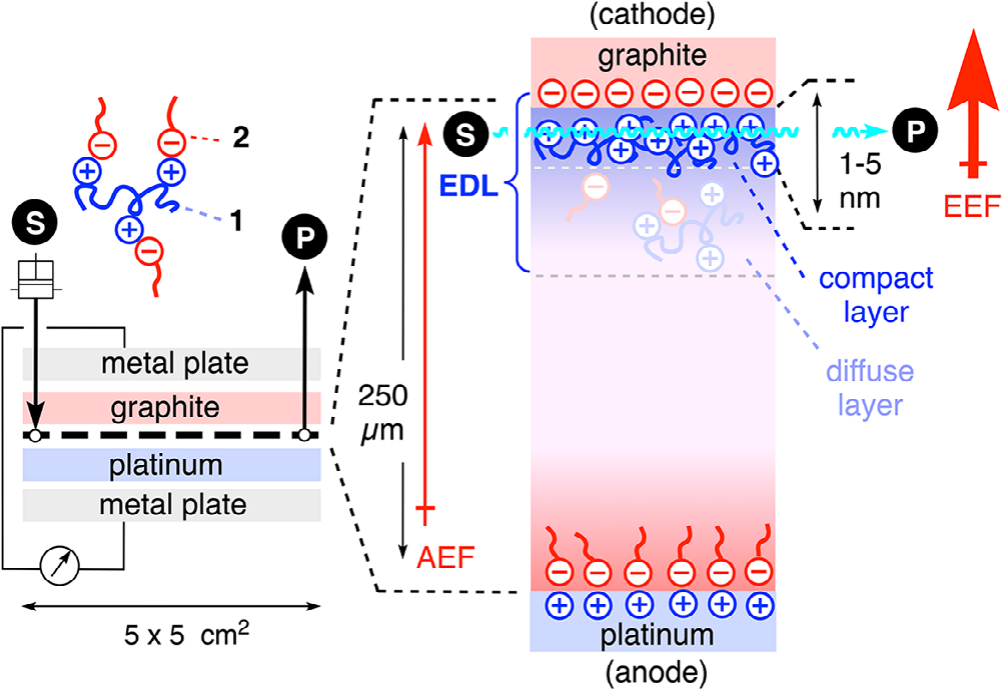
Design of microfluidic capacitors composed of graphite and platinum electrodes separated by a *d* = 250 µm foil with the flow channel and equipped with syringe pump and potentiostat. To construct organocatalytic EDL architectures, pR **1** and SDS **2**, for instance, are injected with the substrate S. While the AEFs are limited to *V*/*d* ≤ 10 mV nm^−1^, EDL architectures engineered to act as supramolecular anodes promise access to *d* = 1–5 nm between the effective electrodes and thus translate the AEFs into much higher effective electric fields (EEFs).

EDLs form at the interface between a liquid and a charged solid, particularly on the electrode surface polarized by an applied voltage (Figure 2).^[^ ^31^, ^32^ ^]^ Since the concept was developed by Helmholtz,^[^ ^33^ ^]^ it has been studied and refined by Gouy, Chapman, Stern and others.^[^ ^31^, ^34^, ^35^ ^]^ In the presence of ion pairs in solution, the first layer of EDLs is mainly composed of ions that are driven by the AEF to the physical electrode of the opposite charge, forming a highly ordered layer of cations on the cathode and anions on the anode. Without ions in polar solvents, this so‐called compact, Helmholtz or Stern layer is composed of uniformly oriented solvent molecules. The second layer of EDLs, the diffuse layer, is less ordered and also contains ion pairs with ions of the charge of the physical electrode.

The potential drops steeply within the Helmholtz layer of thickness *d* ∼ 1–5 nm, resulting in high EEFs. Intrinsic EDLs have been regularly considered as contributors to EFC in different formats.^[^ ^2^, ^17^, ^18^ ^]^ In electromicrofluidic reactors at *V* = 2 V, already EDLs from an oriented polar solvent, MeCN, have been reported to produce an EEF = 1.3 V nm^−1^.^[^ ^17^ ^]^ This is likely an underestimate due to the unknown local dielectric constant,^[^ ^31^ ^]^ but the minimum electric field of 1 V nm^−1^ required for EFC^[^ ^1^, ^15^ ^]^ was reached also with the least favorable assumptions. This solvent EEF increased with applied voltage and promoted surface modification.^[^ ^17^ ^]^

In 2003, we reported that polyarginine (pR) **1** phase transfers and dissolves into organic solvents if the right amphiphilic counterions are present, like (sodium) dodecylsulfate (SDS) **2** (Figure 2).^[^ ^30^ ^]^ At that time, this was of interest to explain how arginine‐rich CPPs could move across biomembranes.^[^ ^30^, ^36^ ^]^ The discovery stimulated a broad screening of amphiphilic counterions that would activate CPPs in biomembranes and other materials,^[^ ^30^, ^36^, ^37^ ^]^ which resulted in the discovery of pyrenebutyrate as the best CPP activators,^[^ ^38^ ^]^ which was joined recently by push–pull aromatics,^[^ ^39^ ^]^ fluorinated fatty acids,^[^ ^39^ ^]^ boron clusters^[^ ^40^ ^]^ and new calixarenes.^[^ ^41^ ^]^

Applying these lessons from nature, we initiated studies on organocatalytic EDL engineering with pR **1** and SDS **2**. With voltage applied, pR **1** should be driven to the cathode to form a compact Helmholtz layer that can serve as a supramolecular anode, generating the EEFs > 1 V nm^−1^ required for EFC (Figure 2). EFC in this engineered Helmholtz layer should be supported by interactions available within the supramolecular architecture itself (mostly hydrogen bonding with pR **1**), cation‐π interactions with the graphite cathode, as well as ionic interactions with ion pairs in the diffuse layer. On the plate anode, SDS **2** should afford the complementary supramolecular cathode in response to the applied voltage.

Proline‐catalyzed asymmetric Robinson annulation of the achiral substrate **3** was considered as benchmark reaction (Figure 3a).^[^ ^8^, ^25^, ^26^, ^27^, ^28^, ^29^, ^42^, ^43^ ^]^ In the Houk–List mechanism, the addition of catalyst **4** is controlled by transition state **TS‐1**, which dehydrates to an iminium intermediate and tautomerizes through **TS‐2** into the enamine intermediate.^[^ ^44^, ^45^ ^]^ Intramolecular aldol condensation through **TS‐3** generates another iminium intermediate, which hydrolyzes into product **5**. Theoretical simulations and small‐molecule catalysts with local electric fields have confirmed the compatibility of proline catalysis with EFC.^[^ ^8^, ^45^ ^]^ With large emerging dipoles, **TS‐1** and the aldolization **TS‐3** leading to iminium intermediates could particularly benefit from EFC.

**Figure 3:**
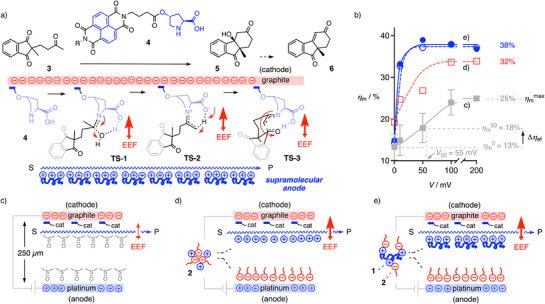
a) Asymmetric Robinson annulation of substrate (S) **3** to products (P) **5** and **6** catalyzed by proline catalyst **4** with an NDI interfacer to the graphite cathode (R = L‐leucine hexylamide, 50 mol%) in DMSO, with key transition states (TS) and possible stabilization by EEF from Helmholtz layers as supramolecular anodes, here composed of polyarginine. b) Microfluidic yield of the aldolization of **3** (50 mM) as a function of applied voltage (platinum minus graphite electrodes) in the presence of catalyst **4** (50 mol%) and EDL components DMSO (filled squares, with schematic interpretation in c, error bars represent SD from experimental duplicates) plus SDS **2** (1 mM, empty squares, d), pR **1** (Cl^–^ salt, 1 mM, monomer unit concentration, empty circles), or both (filled circles, e), with definitions of effective voltage *V*_50_, EFC contribution Δ*η*_ef_, and maximal microfluidic yield *η*_m_^max^ at saturation.

Freshly polished graphite electrodes were selected as cathodes in the envisioned microfluidic capacitor because their structure changes the least with applied voltage, and their aromatic surface offers voltage‐induced ion‐π interactions to contribute to EFC.^[^ ^20^ ^]^ In catalyst **4**, the proline was attached to a naphthalenediimide (NDI) interfacer. π‐Acidic and popular in anion‐π catalysis,^[^ ^46^ ^]^ NDI interfacers were expected to π‐stack to the π‐basic graphite surfaces serving as cathodes. A leucine hexylamide tail R was attached to the other NDI imide to increase solubility.

Extensively optimized for decades, proline‐catalyzed Robinson annulation works best in DMSO, and is incompatible with more and less polar solvents because of various reasons, including solubility.^[^ ^8^, ^25^ ^]^ The EFC reactions were performed by pumping solutions of the substrate **3** and, from a separate syringe, catalyst **4**, both in DMSO containing the EDL components, such as pR **1** and SDS **2**, into the microfluidic capacitor under constant voltage. The formation of product **5** and the trace product **6** were monitored after one passage through the microfluidic capacitor, and their sum is reported as microfluidic yield *η*_m_. At constant flow rate and concentrations, reproducibility was reasonable (Figure 3b, gray curve). Without applied voltage but with 50 mol% catalyst **4** in DMSO as solvent, *η*_m_^0^ = 13% was recorded (Figure 3b, filled squares). With increasing voltage, yields increased with a half‐maximal effective voltage *V*_50_ = 55 mV to a maximal *η*_m_^max^ = 25% at saturation.

Without applied voltage, the presence of SDS **2** increased the yield to *η*_m_^0^ = 20% (Figure 3b, empty squares). This increase might relate to the formation of intrinsic EDLs even without the application of voltage. With voltage applied, yields increased with *V*_50_ = 10 mV to *η*_m_^max^ = 32%. This trend continued with pR **1** to *V*_50_ < 10 mV to *η*_m_^max^ = 38%. The correlation of increasing EFC with decreasing *V*_50_ suggested that the best‐performing supramolecular electrodes assemble already at very low voltage, whereas the solvent EDL needs higher voltage to form. The high activity of pR **1** was consistent with interfacing of the catalyst **4** with the cathode (Figure 3e). Compared to *η*_m_^0^ = 13% without voltage, the yield increased ≈3 times to *η*_m_^max^ = 38%. This compared favorably to the only ≈2‐fold increase to *η*_m_^max^ = 25% obtained with the intrinsic EDL from the polar solvent. Saturation reached already below *V* = 50 mV (Figure 3b, circles) calculated to AEF = *V*/*d* < 50 mV/250 µm < 200 nV nm^−1^ at maximal performance (Figure 2). Considering the theoretical onset of EFC at 1 V nm^−1^ and experimental EDL data above this threshold,^[^ ^17^ ^]^ this suggested that EEFs from engineered supramolecular EDL anodes exceed the AEF by more than 5 million.

To screen organocatalytic EDL architectures, EFC contributions to microfluidic systems were evaluated by comparing the microfluidic yields *η*_m_ at 0 and 50 mV (Figure 4b). Their difference Δ*η*_ef_ = *η*_m_^50^ – *η*_m_^0^ should represent the additional effect of the AEF to the intrinsic EDL formed at 0 mV (Figure 4a). For the solvent EDL, Δ*η*_ef_ = +5% was obtained, which increased up to Δ*η*_ef_ = +24% with a mixture of **1** and **2** (Figure 4a, bar 1, empty, vs bar 3, red). pR **1** without **2** was similar, whereas SDS **2** without **1** was much weaker. The linear naphthyl analog of substrate **3** gave Δ*η*_ef_ = +8% without and Δ*η*_ef_ = +33% with **1** and **2** (Figure S14). Already *η*_m_^0^ = 20% was 6% higher, which increased to *η*_m_^max^ = 53% with **1** and **2**.

**Figure 4:**
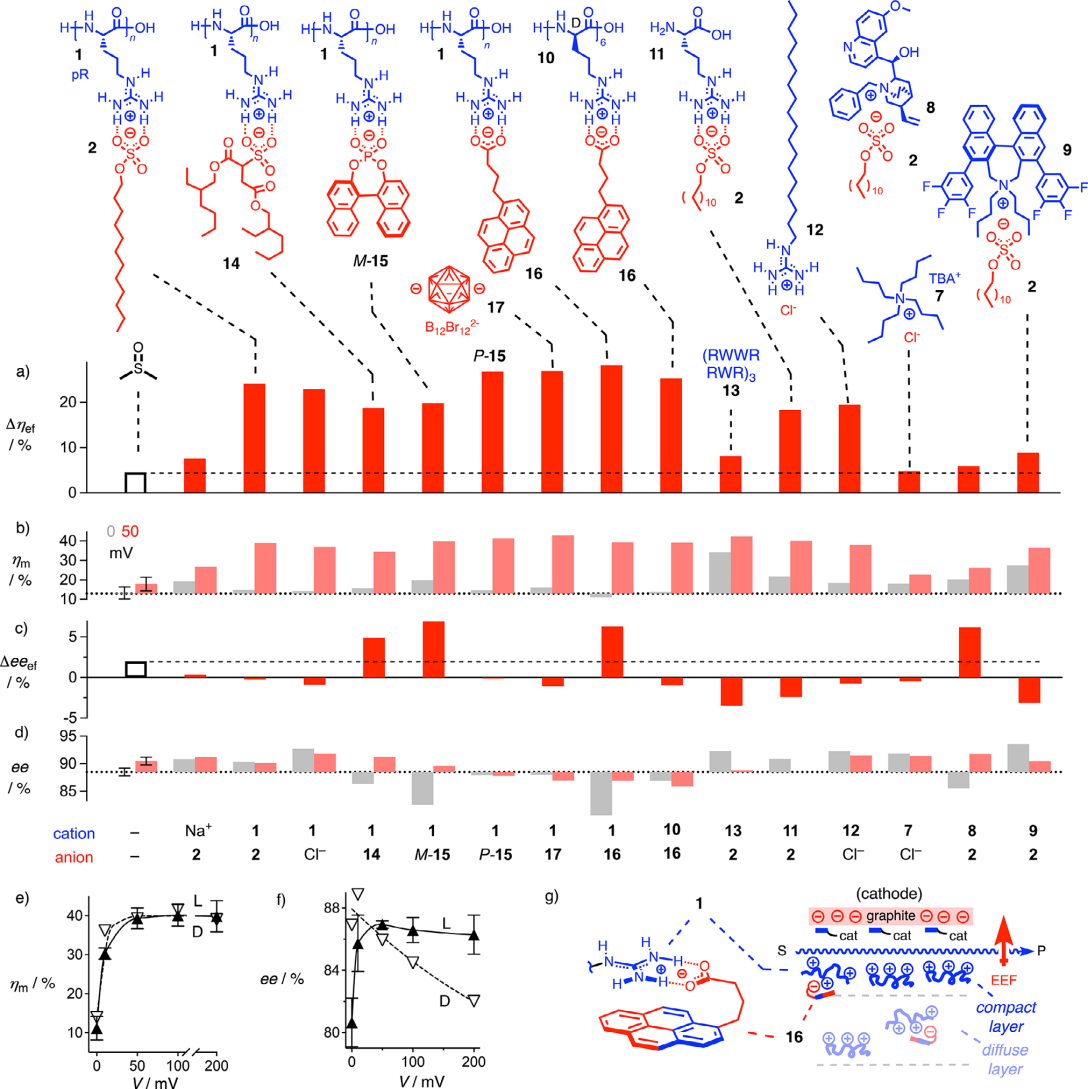
a) Increase in yield Δ*η*_ef_ per microfluidics passage from *η*_m_^0^ at *V* = 0 mV to *η*_m_^50^ at *V* = 50 mV for the aldolization of **3** (50 mM) with **4** (50 mol%) in the absence (empty) and the presence (red) of additional EDL components 1, 2, **7**–**17** (1 mM, monomer unit concentration) in DMSO. b) Microfluidic yield *η*_m_^0^ at *V* = 0 mV (gray) and *η*_m_^50^ at *V* = 50 mV (pale red). c) Changes of enantioselectivity Δ*ee*_ef_ from *ee*_m_^0^ at *V* = 0 mV to *ee*_m_^50^ at *V* = 50 mV. d) Enantioselectivity *ee*_m_^0^ at *V* = 0 mV (gray) and *ee*_m_^50^ at *V* = 50 mV (pale red). e) Microfluidic yield of the aldolization of **3** as a function of applied voltage in the presence of catalyst **4**, pyrenebutyrate **16** and either L‐R_n_ **1** (upward filled triangles) or D‐R_6_ **10** (downward empty triangles). f) Enantioselectivity of the EFC in e. g) Interpretation of asymmetric EFC originating from the achiral pyrenebutyrate **16** with ion pair‐π complexes between **1**/**10** and **16** at the interface between the compact and diffuse layer of the EDL.

Organic cations other than guanidinium, including tetrabutylammonium (TBA) cations as in TBACl **7**, were inferior (Figure 4a,b). Replacement of **7** with the alkylated quinine **8** ^[^ ^47^ ^]^ or the binaphthyl‐based Maruoka phase‐transfer catalyst **9** ^[^ ^48^ ^]^ did not afford very active EDL architectures either. In contrast, EFC with almost all variants **10**–**13** of guanidinium cations was excellent. Only the designed amphiphilic α helix **13** complemented with π‐basic tryptophanes gave a high *η*_m_^0^ = 34% (Figure 4b, gray), and thus a poor Δ*η*_ef_ = +8% response to AEF (Figure 4a). This finding implied that this peptide, perhaps assisted by π–π interactions between tryptophan residues and the graphite cathode surface, provides access to significant intrinsic EDL architectures. Enantiomeric D‐hexa‐arginine **10** caused only minor decreases, arginine monomers **11** paired with SDS **2** dropped to still important Δ*η*_ef_ = +18%. Promising for future use in practice, the minimalist octadecylguanidinium **12** paired with chloride gave important Δ*η*_ef_ = +20%. Considering the known oligomer effect on capacitance,^[^ ^49^ ^]^ this weak dependence on oligomer lengths implied that such effects are overcompensated by the impact of the supramolecular EDL architectures.

Paired with dioctyl sulfosuccinate **14** and binaphthyl phosphodiesters *M*‐ and *P*‐**15**, EFC with supramolecular pR **1** anodes remained around *η*_m_^50^ ≈ 40%, the Δ*η*_ef_ of binaphthyl *P*‐**15** was nearly top. As for cellular uptake,^[^ ^38^ ^]^ pyrenebutyrate **16** gave the best Δ*η*_ef_ = +28% with pR **1**. Compared to solvent EDLs, the relevant EFC contribution Δ*η*_ef_ increased 6 times. Contrary to cellular uptake,^[^ ^40^ ^]^ pairing with boron clusters **17** was slightly weaker.

Under standard conditions at 0 mV in DMSO without additives, proline **4** catalyzed the annulation of **3** with *ee*_m_^0^ = 89% (Figure 4d). EFC contributions from the AEF added to this high intrinsic enantioselectivity were isolated as Δ*ee*_ef_ (Figure 4c). The strongest increases originated from reduced *ee*_m_^0^ rather than increased *ee*_m_^50^. For binaphthyl anions paired with **1**, a record Δ*ee*_ef_ was observed for *M*‐**15** but not for *P*‐**15**, which had near record Δ*η*_ef_. The achiral pyrenebutyrate **16** gave top Δ*ee*_ef_ and Δ*η*_ef_ paired with L‐polyarginine **1**, whereas pairing with the D‐oligomer **10** canceled Δ*ee*_ef_ increases without changing Δ*η*_ef_ much. These trends suggested that the chiral environment provided by oligo/polyarginines complexed with **16** determines Δ*ee*_ef_. It disappears when the AEF drives the anions out of assembling compact cationic layer where EFC occurs.

The voltage dependence of *η*_m_ with **16** and either L‐polymer **1** or D‐oligomer **10** was the same, characterized by *V*_50_ ≈ 10 mV (Figure 4e). The voltage dependence of *ee*_m_ with L‐polymer **1** reproduced this curve (Figure 4f, filled symbols), whereas *ee*_m_ from the D‐oligomer **10** paired with **16** decreased with a much higher *V*_50_ > 70 mV (Figure 4f, empty symbols). This high effective voltage could hint at contributions from the diffuse layer of the EDL to EFC, here to disturb the chirality of the active Helmholtz layer formed by the polycations (Figure 4g).

Control experiments confirmed that EFC in microfluidic double‐layer capacitors is insensitive to the presence of one equivalent of TEMPO (Figure S15). This established test^[^ ^50^ ^]^ confirmed that contributions from electron transfer and radical redox chemistry are negligible. EFC with alternating current (AC, from 16 to 200 Hz, and 50 to 140 mV) was similar to EFC with direct current (DC, Figure S16). This result was consistent with the fast formation of supramolecular EDL architectures. While the basal conditions chosen for EDL development give *η*_m_^0^ = 13% and limit the maximum yield to *η*_m_^max^ < 50%, higher microfluidic yield can naturally be achieved by using lower flow rates (Figure S17) or higher catalyst concentrations, among others. Nevertheless, we noted the poor compatibility of our microfluidics system with kinetics studies, which remains a technical limitation. Microfluidic yields *η*_m_ refer to a specific reaction time given by the flow rate, and changes in flow rate affect parameters other than reaction time. Different electrodes gave similar results (Figure S18), and positive preliminary results were obtained for other reactions (not shown, ongoing studies).

While it is understood that a benchmark reaction that performs intrinsically above 80% *ee* leaves little scope to improve, it is important to respect that the same ambitious point of reference applies to conversion: EFC with the best supramolecular EDL architectures triples the yield of one of the most optimized reactions in proline organocatalysis. However, the objective of this study was neither record performances nor high numbers of reactions covered. The objective was to establish a method enabling scalable EFC and then develop it with one benchmark reaction as a readout to a level of understanding that can serve as a guide to elaborate on all the diverse perspectives that emerge. These include different reactions (at best, otherwise impossible ones), different catalysts and their positioning, different electrodes and their modifications, different flow reactors, different solvents and, most importantly, different organocatalytic supramolecular EDL architectures. While new methods to access scalable EFC have been predicted to impact organic chemistry broadly, enabling efforts to improve on sustainable industrial production and explore the origin of life, it is predictable that polyarginine and pyrenebutyrate will not remain the organocatalytic EDL architectures of choice for most applications. Depending on the topic of interest, their nature will vary within the rich molecular structural space that is opening up for exploration.

## Supporting Information

Experimental details.

## Conflict of Interests

The authors declare no conflict of interest.

## Supporting information



Supporting Information

## Data Availability

The data that support the findings of this study are openly available in zenodo at https://doi.org/10.5281/zenodo.17052869.
