# Supplementary material for: Organocatalytic Microfluidic Double‐Layer Capacitors
Source: Angew Chem Int Ed Engl. 2025 Sep 21;64(45):e202517078. doi: 10.1002/anie.202517078 (PMC12582001; doi:10.1002/anie.202517078)
Supplement: Supplementary file 1 — Supporting Information [file ANIE-64-e202517078-s001.pdf]

Supporting Information

Organocatalytic Microfluidic Double-Layer Capacitors

Shen-Yi Guo, Miguel Paraja, Augustina Jozeliūnaitė, Manuel Gallardo Villagran, Qing-Xia Zhang, Alenka Marsalek, Naomi Sakai, and Stefan Matile*[a,b]

[a]Department of Organic Chemistry, University of Geneva, Geneva, Switzerland

[b]National Centre of Competence in Research (NCCR) Molecular Systems Engineering, Basel, Switzerland

*E-mail: stefan.matile@unige.ch

Table of Contents

1. Material and Methods S2

2. Synthesis S3

   2.1. Synthesis of Substrates and Products S3

   2.2. Synthesis of the Catalyst S4

3. Product Analysis S7

   3.1. HPLC Resolution of the Reaction Mixtures S7

   3.2. HPLC Analysis Method S9

   3.3. Calibration Factors S11

4. Electric-Field Catalysis S14

   4.1. Electromicrofluidic Reactor Configuration S14

   4.2. General Procedure for Electric-field Catalysis S15

   4.3. EFC in Intrinsic EDL S18

   4.4. EFC in Engineered EDL S20

   4.5. TEMPO Control S28

   4.6. EFC with Alternating Current S30

   4.7. EFC with Different Flowrates S31

   4.8. EFC with Different Electrodes S33

5. Supplementary References S34

6. HPLC Chromatograms S35

   6.1. Reference Compounds S35

   6.2. Reaction Mixtures S42

7. NMR Spectra S67

1. Material and Methods

As in reference,[S1] reagents for synthesis were purchased from Sigma-Aldrich, TCI, and Acros. Polyarginine hydrochloride (MW 5000-15000) was purchased from Sigma-Aldrich. SDS was purchased from PanReac AppliChem. Hexa-D-arginine was purchased from TargetMol. A RW peptide [$(RWWRRWR)_3$] was custom synthesized by CASLO ApS. Boron cluster $Na_2[B_{12}Br_{12}]$ was purchased from Katchem spol. s.r.o. Unless otherwise specified, all chemicals were used as received. Flash column chromatography was performed on a Biotage Isolera™ system. Analytical and preparative TLCs were performed on silica gel 60 F^{254} (Merck) and silica gel (SiliCycle, 1000 µm), respectively. Room temperature (rt) stands for 20-25 °C. Melting points (Mp) were measured on a Melting Point M-565 (BUCHI). Alpha-D values were measured on a Polarimeter P-1030 (Jasco). IR spectra were recorded on a PerkinElmer FTIR spectrum two+ (ATR, Golden Gate) and are reported as wavenumbers (ν) in cm^{-1} with band intensities indicated as s (strong), m (medium), w (weak), and br (broad). ^1H and ^{13}C NMR were recorded on a Bruker 400 MHz spectrometer and are reported as chemical shifts (δ) in ppm relative to the residual solvent resonance ($CHCl_3$, δ = 7.26, $CDCl_3$, 77.2). Spin multiplicities are reported as a singlet (s), doublet (d), triplet (t) and quartet (q), with coupling constants (J) given in Hz, or multiplet (m). Broad peaks are marked as br. ESI-MS was measured using Advion expression CMS with Advion plate express TLC/CMS and reported as *m/z*. Accurate mass determinations using ESI (HR ESI-MS) were performed on Xevo G2-S Tof (Waters).

Abbreviations. AOT: Dioctyl Sulfosuccinate; Boc: *tert*-Butyloxycarbonyl; DIPEA: N,N-Diisopropylethylamine; DMAP: 4-Dimethylaminopyridine; DMF: Dimethylformamide; DMSO: Dimethyl sulfoxide; EDC·HCl: 1-Ethyl-3-(3-dimethylaminopropyl) carbodiimide hydrochloride; EDL: Electrical double layers; *ee*: Enantiomeric excess; EF: Electric field; EFC: Electric-field catalysis; EtOAc: Ethyl acetate; FEP: Fluorinated ethylene propylene; Gr:

Graphite electrode; OEEF: Oriented external electric field; pR: Poly-L-arginine; Pt: Platinum electrode; rt: Room temperature; SDS: Sodium dodecyl sulfate; TBA: tetrabutylammonium; TEMPO: 2,2,6,6-Tetramethylpiperidinyloxyl; TFA: Trifluoroacetic acid; TfOH: Trifluoromethanesulfonic acid.

2. Synthesis

2.1. Synthesis of Substrates and Products

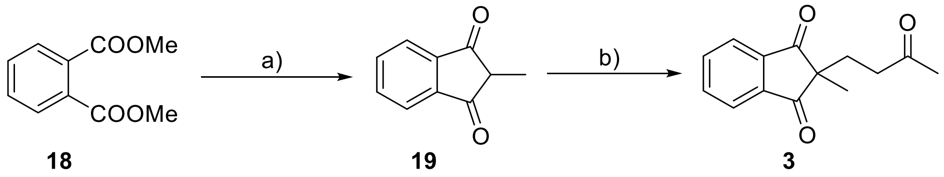

Scheme S1. Synthesis of substrate **3**. (a) 1. Methyl propionate, NaH, Bu_2O, reflux, 2 h; 2. NaH, Bu_2O, reflux, 14 h. (b) Methyl vinyl ketone, Et_3N, CH_2Cl_2, rt, 30 min, 68% in two steps.

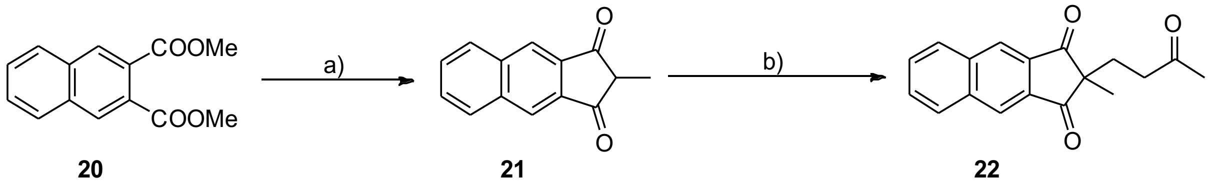

Scheme S2. Synthesis of substrate **22**. (a) 1. Methyl propionate, NaH, Bu_2O, reflux, 2 h; 2. NaH, Bu_2O, reflux, 48 h. (b) Methyl vinyl ketone, Et_3N, CH_2Cl_2, rt, 48 h, 30% in two steps.

Compounds 3 and 22 were prepared following the reported procedures.[S2]

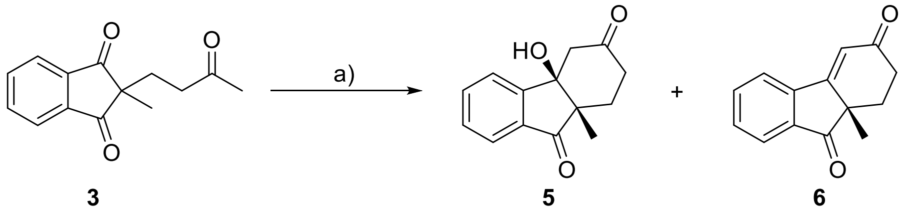

Scheme S3. Synthesis of products **5** and **6**. (a) *S*-proline (30 mol%), DMF, rt, 48 h, **5** (7%), **6** (48%).

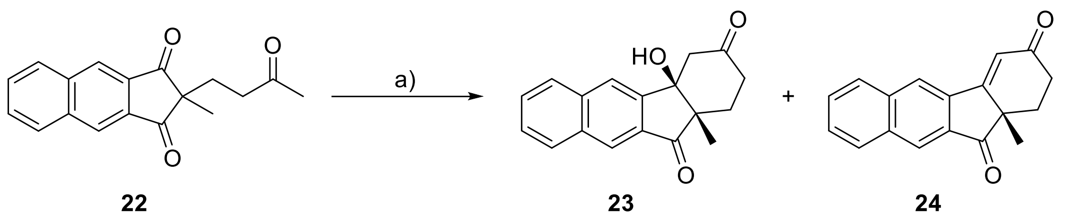

Scheme S4. Synthesis of products **23** and **24**. (a) *S*-proline (30 mol%), DMF, rt, 48 h, **23** (35%), **24** (26%).

Compounds 5, 6, 23, and 24 were prepared following the reported procedures.[S2]

The synthesized substrates **3** and **22**, as well as the products **5**, **6**, **23**, and **24**, were analysed by HPLC to determine their retention times and ensure peak resolution (Figures S20-S22, S25-S29), thereby enabling direct analysis of the reaction mixtures (see section 3.1).

2.2. Synthesis of the Catalyst

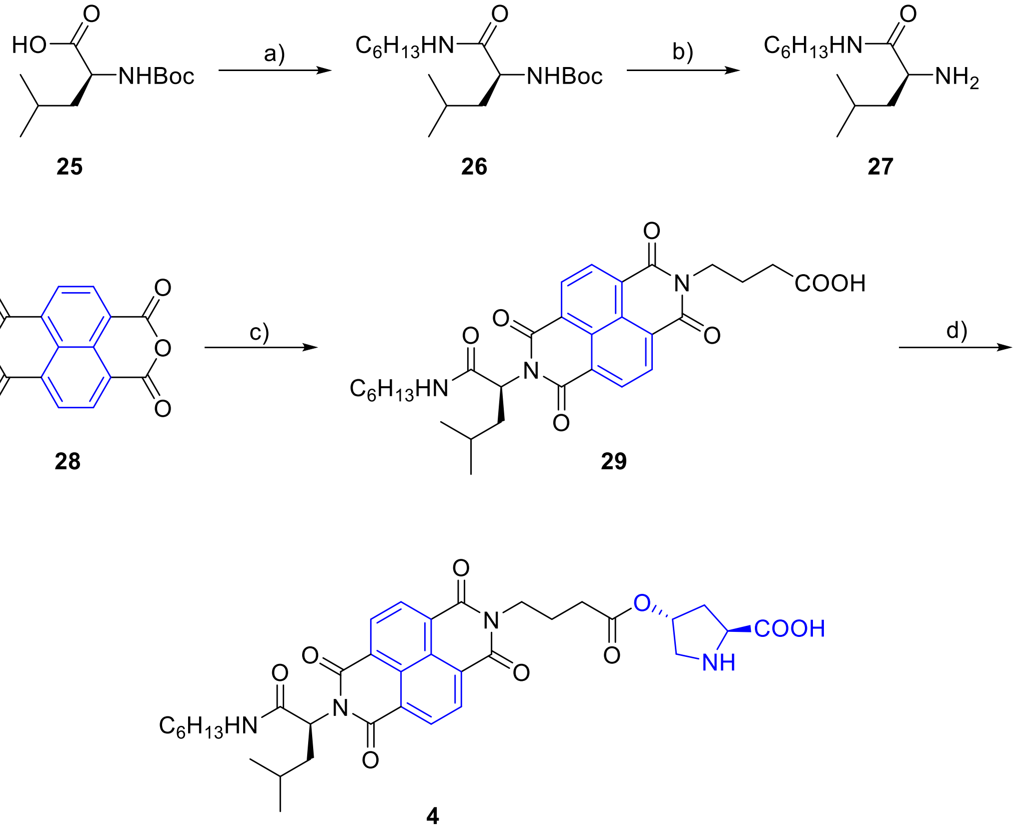

Scheme S5. Synthesis of catalysts **4**. (a) Hexylamine, DMAP, EDC·HCl, CH_2Cl_2, rt, 4 h, 93%. (b) 1. TFA, CH_2Cl_2, rt, 4 h; 2. $NaHCO_3$, H_2O, rt, 15 h, quat. (c) 1. **27**, KH_2PO_4/K_2HPO_4, H_2O,

pH=6.3, reflux, 72 h; 2. 4-Aminobutyric acid, DIPEA, DMF, 90 °C, 15 h, N_2, 63%. (d) 1. Cl_3CCN, PPh_3, $CHCl_3$, rt, 1 h, quat; 2. *trans*-4-Hydroxy-*S*-proline, TFA, TfOH, rt, 3 h; 3. Propylene oxide, MeOH, rt, 4 h, 54%.

Compound 27 was prepared following the reported procedures.[S3]

Compound 29. To a suspension of 1,4,5,8-naphthalene-tetracarboxylic dianhydride **30** (3.35 g, 12.5 mmol) in water (550 mL) was added 1 M KOH aqueous solution (70 mL). The solid dissolved and gave a yellow transparent solution. The pH was adjusted to 6.3 using 1 M H_3PO_4 aqueous solution, then a solution of **27** (2.68 g, 12.5 mmol) in DMF (10 mL) was added, and the pH was adjusted again to 6.3 using 1 M H_3PO_4 aqueous solution. After the solution was refluxed for 72 h, the reaction was cooled to room temperature and the pH was adjusted to 1~2 using 1 M HCl aqueous solution to give a yellow solid. The solid (5.12 g) were collected by filtration, washed with water, and dried under vacuum overnight. Then, the solid was dissolved in dry DMF (85 mL). 4-Aminobutanoic acid (2.06 g, 20.0 mmol) and DIPEA (3.54 mL, 20.0 mmol) were added to the solution, and the reaction mixture was stirred at 90 °C for 15 h under nitrogen atmosphere. Afterward, most of the solvent was evaporated in vacuo, and the residue was dissolved in MeOH (70 mL). Water (150 mL) was added to this solution, and then the pH was adjusted to 3 with 1 M HCl aqueous solution. The mixture was extracted with EtOAc (300 mL × 1, 50 mL × 3). The combined organic phases were dried over anhydrous Na_2SO_4. After filtration and removal of solvent, the residue was chromatographed on a silica gel column (EtOAc/MeOH 19:1) to give pure product **29** (4.35 g, 63%) as a yellow solid. R_f (EtOAc): 0.3; $[\alpha]_D^{20}$ -8.5 (*c* 1.0, $CHCl_3$); Mp: 140-141 °C; IR (neat): 3281 (br, O-H), 2932 (w, C-H), 2867 (w, C-H), 1707 (s, C=O), 1645 (s, C=O), 1581 (m, C=C), 1539 (m, C=C), 1453 (m, C=C), 1332 (s, C-N), 1246 (s, C-O), 1185 (m, C-O), 764 (m); ^1H NMR (400 MHz, $CDCl_3$): 8.76 (d, $^3J_{H-H}$ = 7.7 Hz, 2H), 8.75 (d, $^3J_{H-H}$ = 7.7 Hz, 2H), 5.85 (t, $^3J_{H-H}$ = 5.4 Hz, 1H), 5.72 (dd, $^3J_{H-H}$ = 10.0 Hz, $^3J_{H-H}$ = 5.1 Hz, 1H), 4.26 (t, $^3J_{H-H}$ = 7.2 Hz, 2H), 3.30 (dt, $^3J_{H-H}$ = 6.8 Hz, $^3J_{H-}$

$_{H}$ = 5.4 Hz, 2H), 2.47 (t, $^{3}J_{H\text{-}H}$ = 7.3 Hz, 1H), 2.34 (ddd, $^{2}J_{H\text{-}H}$ = 13.6 Hz, $^{3}J_{H\text{-}H}$ = 10.0 Hz, $^{3}J_{H\text{-}H}$ = 4.6 Hz, 1H), 2.07 (tt, $^{3}J_{H\text{-}H}$ = 7.3 Hz, $^{3}J_{H\text{-}H}$ = 7.2 Hz, 2H), 1.92 (ddd, $^{2}J_{H\text{-}H}$ = 13.6 Hz, $^{3}J_{H\text{-}H}$ = 9.2 Hz, $^{3}J_{H\text{-}H}$ = 5.1 Hz, 1H), 1.58 – 1.46 (m, 3H), 1.37 – 1.24 (m, 6H), 1.01 (d, $^{3}J_{H\text{-}H}$ = 6.5 Hz, 3H), 0.95 (d, $^{3}J_{H\text{-}H}$ = 6.5 Hz, 3H), 0.87 (t, $^{3}J_{H\text{-}H}$ = 7.0 Hz, 3H); ^{13}C NMR (101 MHz, $CDCl_3$): 175.8 (CO), 169.2 (CO), 163.1 (2CO), 163.0 (2CO), 131.5 (2CH), 131.2 (2CH), 127.1 (C), 126.8 (C), 126.7 (4C), 54.1 (CH), 40.3 (CH_2), 40.1 (CH_2), 37.9 (CH_2), 31.6 (CH_2), 31.2 (CH_2), 29.6 (CH_2), 26.7 (CH_2), 25.9 (CH), 23.5 (CH_3), 23.2 (CH_2), 22.7 (CH_2), 22.2 (CH_3), 14.2 (CH_3); MS (ESI): 550 ($C_{30}H_{36}N_3O_7$, $[M+H]^+$).

Compound 4. This procedure was inspired by literature[S4] and with modification. To a dry round bottom flask, **29** (1.10 g, 2.00 mmol) and triphenylphosphine (1.57 g, 6.00 mmol) were dissolved in dry chloroform (40 mL). Trichloroacetonitrile (0.30 mL, 3.0 mmol) was then added dropwise to this solution. After the mixture was stirred at room temperature for 1 hour, the solvent was evaporated under reduced pressure. To the residue was added dropwise a solution of *trans*-4-hydroxy-*S*-proline (0.525 g, 4.00 mmol) in TFA (3.60 mL) and TfOH (0.40 mL). The mixture was stirred at room temperature for another 3 hours, and then water (50 mL) and saturated $NaHCO_3$ aqueous solution (20 mL) was slowly added to this solution under an ice bath. The aqueous phase was extracted with CH_2Cl_2 (50 mL × 2). The combined organic phases were concentrated and dissolved in MeOH (50 mL). Propylene oxide (0.42 mL, 6.00 mmol) was added to this solution to remove the residual acid. After the solution was stirred for 4 hours, the solvent was evaporated under reduced pressure and residue was chromatographed on a reverse-phase C18e-HP column (H_2O/CH_3CN 1:1) to give compound **4** (712 mg, 54%) as a colorless solid. Mp: decomposed >143 °C; $[\alpha]_D^{20}$ -28 (*c* 1.0, $CHCl_3$); IR (neat): 3327 (br, O-H), 2933 (w, C-H), 2866 (w, C-H), 1707 (s, C=O), 1662 (s, C=O), 1581 (m, C=C), 1536 (m, C=C), 1452 (m, C=C), 1334 (s, C-N), 1247 (s, C-O), 1183 (m, C-O), 1164, (m, C-O), 770 (m); ^{1}H NMR (400 MHz, $CDCl_3$): 8.67 (s, 4H), 6.12 (t, $^{3}J_{H\text{-}H}$ = 6.1 Hz, 1H), 5.58 (dd, $^{3}J_{H\text{-}H}$ = 10.0

Hz, $^3J_{H-H}$ = 4.9 Hz, 1H), 5.33 (br s, 1H), 4.26 – 4.08 (m, 3H), 3.77 – 3.42 (m, 2H), 3.24 (dt, $^3J_{H-H}$ = 6.2 Hz, $^3J_{H-H}$ = 6.1 Hz, 2H), 2.55 – 2.40 (m, 3H), 2.34 – 2.16 (m, 2H), 2.03 (tt, $^3J_{H-H}$ = 6.9 Hz, $^3J_{H-H}$ = 6.9 Hz, 2H), 1.92 (ddd, $^2J_{H-H}$ = 13.8 Hz, $^3J_{H-H}$ = 9.0 Hz, $^3J_{H-H}$ = 4.8 Hz, 1H), 1.54 – 1.39 (m, 3H), 1.33 – 1.23 (m, 6H), 0.94 (d, $^3J_{H-H}$ = 6.6 Hz, 3H), 0.89 (d, $^3J_{H-H}$ = 6.6 Hz, 3H), 0.86 (t, $^3J_{H-H}$ = 7.0 Hz, 3H); ^{13}C NMR (101 MHz, $CDCl_3$): 173.2 (CO), 172.3 (CO), 169.1 (CO), 162.9 (4CO), 131.4 (2CH), 131.2 (2CH), 126.9 (C), 126.7 (2C), 126.6 (C), 126.5 (2C), 73.2 (C), 60.2 (C), 54.0 (CH), 50.3 (CH_2), 40.2 (CH_2), 40.0 (CH_2), 37.8 (CH_2), 35.7 (CH_2), 31.6 (CH_2), 31.6 (CH_2), 29.6 (CH_2), 26.7 (CH_2), 25.8 (CH), 23.5 (CH_3), 23.1 (CH_2), 22.7 (CH_2), 22.2 (CH_3), 14.2 (CH_3); HRMS (ESI): calcd. for $C_{35}H_{42}N_4O_9$ ($[M+H]^+$): 663.3025, found: 663.2995.

3. Product Analysis

3.1. HPLC Resolution of the Reaction Mixtures

1,3-Di-tert-butyl-2-methoxybenzene **30** was chosen as an optimal internal standard based on three attributes: (i) steric hindrance from the tert-butyl groups that effectively prevents π-stacking interactions with the graphite surface, (ii) excellent chemical stability under the experimental conditions, and (iii) a strong absorption signature for reliable detection.

The HPLC method was optimized to resolve all the peaks, enabling direct analysis of the reaction mixture.

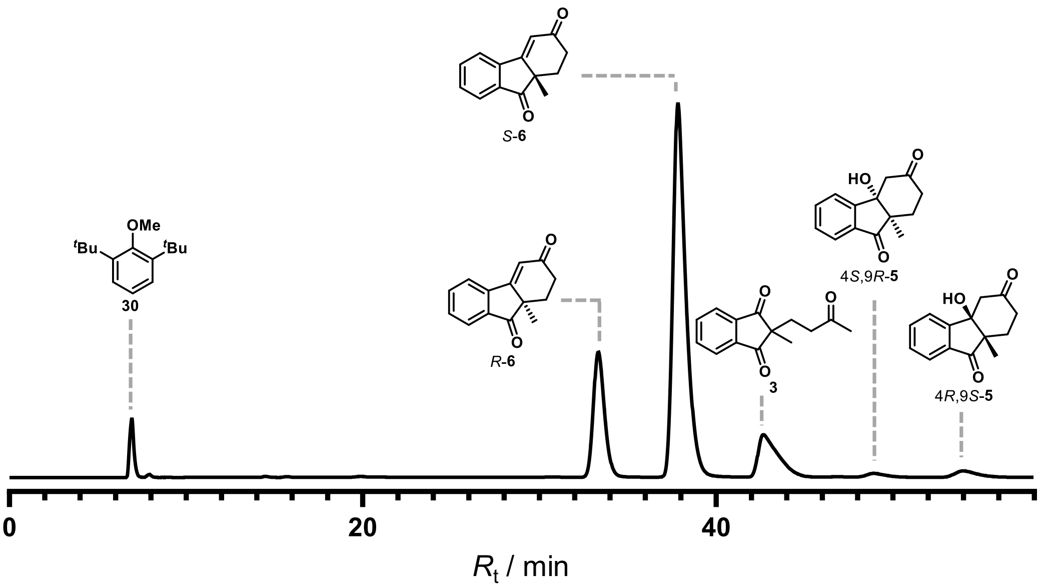

Figure S1. Representative HPLC profile showing the resolution of substrate **3** and products **5**, **6**. HPLC method: CHIRALPAK® ID (4.6 mm Ø x 25.0 cm *l*), 25 ºC, 0.5 mL/min flow rate, Hexane/^{i}PrOH/EtOAc 18:1:1, detection at λ_{abs} = 270 nm. The mixture was used as one of the samples for the calibration curve (Figures S3-S5). The complete HPLC profile of the calibration samples and the molar ratios of the substrate and products relative to the internal standard are shown in Figure S28.

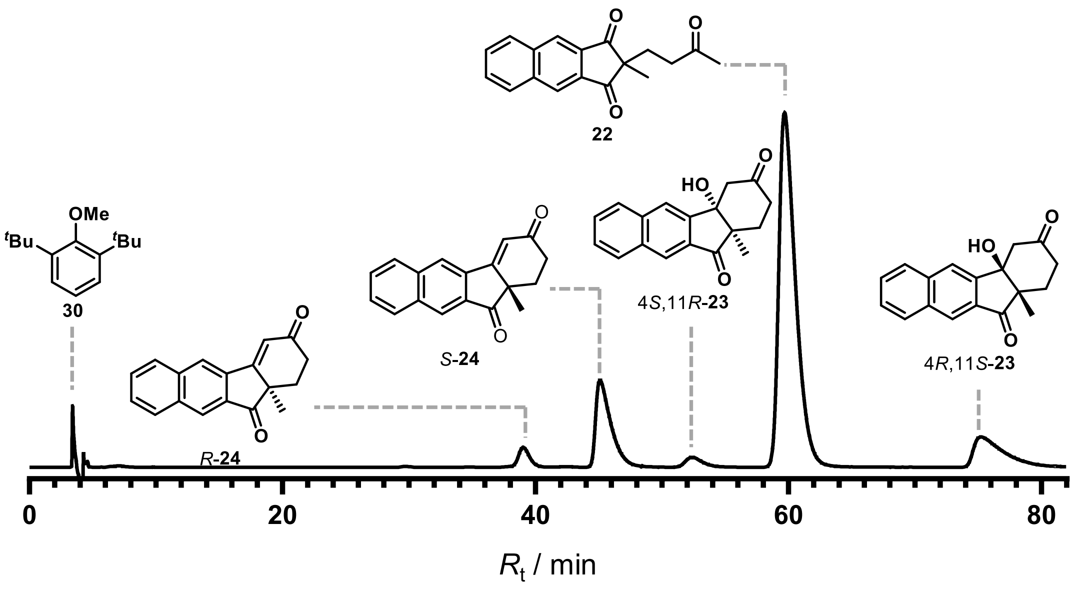

Figure S2. Representative HPLC profile showing the resolution of substrate **22** and product **23**, **24**. HPLC method: CHIRALPAK® ID (4.6 mm Ø x 25.0 cm *l*), 25 °C, 1.0 mL/min flow rate, Hexane/^{i}PrOH/EtOAc 188:7:5, detection at λ_{abs} = 257 nm. The mixture was used as one of the samples for the calibration curve (Figures S6-S8). The complete HPLC profile of the calibration samples and the molar ratios of the substrate and products relative to the internal standard are shown in Figure S29.

3.2. HPLC Analysis Method

Calibration lines were generated from five calibration mixtures, each containing similar amount of internal standard **30** along with varying amounts of substrates and products (Figures S3-S8). Corresponding peak area ratios were determined from HPLC profile. (Figures S28 and S29) By performing a linear regression analysis constrained through the origin, a plot of the ratio of the substance concentrations (c_x/c_{IS}) versus the ratio of the area (A_x/A_{IS}) yields a straight line whose slope gives the calibration factor, as shown in equation S1:

$$\frac{c_x}{c_{IS}} = CF \cdot \frac{A_x}{A_{IS}} \quad \text{(S1)}$$

Where CF is the calibration factor, c_{x} is the analyte concentration, c_{IS} is the concentration of the internal standard **30**, A_{x} is the area of the analyte, and A_{IS} is the area of the internal standard **30**.

The conversion was calculated based on equations S2-4:

$$c_{sub} = CF \cdot \frac{A_{sub}}{A_{\mathrm{IS}}} c_{\mathrm{IS}} \tag{S2}$$

$$c_{sub,0} = \frac{c_{\mathrm{IS}}}{Eq_{\mathrm{IS}}} \tag{S3}$$

Where $c_{\mathrm{sub,0}}$ is the initial substrate concentration, and Eq_{IS} is the equivalent of the internal standard.

$$conversion = 1 - \frac{c_{sub}}{c_{sub,0}} = 1 - CF \cdot Eq_{\mathrm{IS}} \cdot \frac{A_{sub}}{A_{\mathrm{IS}}} \tag{S4}$$

The yield was calculated based on equation S5-6:

$$c_x = CF \cdot \frac{A_x}{A_{\mathrm{IS}}} c_{\mathrm{IS}} \tag{S5}$$

$$yield_x = \frac{c_x}{c_{sub,0}} = CF \cdot Eq_{\mathrm{IS}} \cdot \frac{A_x}{A_{\mathrm{IS}}} \tag{S6}$$

Since product **6** is formed through dehydration of product **5**, the combined yield η_{m} will be presented for brevity.

$$\eta_m = yield_5 + yield_6 \tag{S7}$$

The *ee* values of each product was calculated based on equation S8:

$$ee = \frac{A_{major} - A_{minor}}{A_{major} + A_{minor}} \tag{S8}$$

In this work, the major enantiomers are the (4*R*,9*S*)-isomer of **5**, the (*S*)-isomer of **6**, the (4*R*,11*S*)-isomer of **23**, and the (*S*)-isomer of **24**. Only the *syn* enantiomeric pair was observed, with no detection of the *anti* products.

Since (*S*)-**6** is formed by dehydration of (4*R*,9*S*)-**5** and (*R*)-**6** is formed by dehydration of (4*S*,9*R*)-**5**, the combined *ee* for the reaction of **3** is calculated based on equation S9:

$$combined\ ee = \frac{yield_{5,major} + yield_{6,major} - yield_{5,minor} - yield_{6,minor}}{yield_{5,major} + yield_{6,major} + yield_{5,minor} + yield_{6,minor}} \quad \text{(S9)}$$

As for reaction of **22**, the combined yield and *ee* were calculated in the same way.

3.3. Calibration Factors

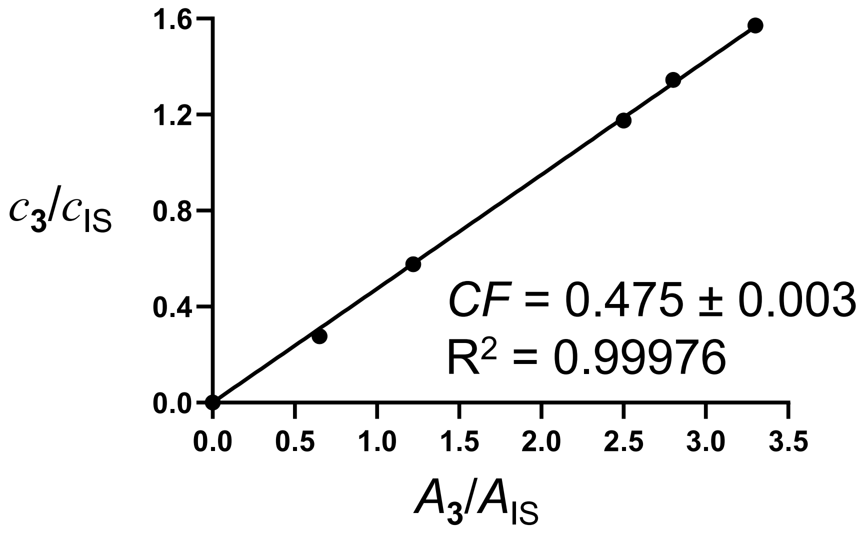

Figure S3. Calibration curve of substrate **3**. HPLC traces are shown in Figure S28.

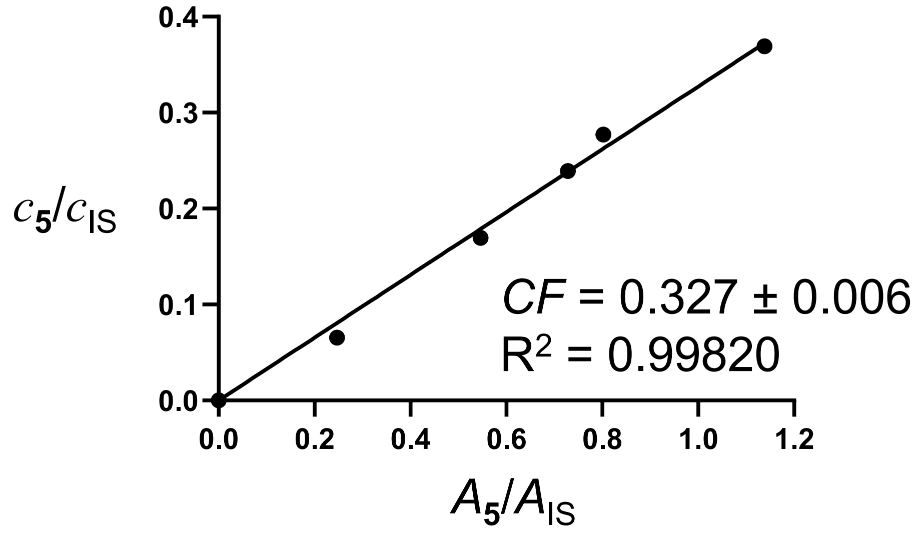

Figure S4. Calibration curve of product **5**. HPLC traces are shown in Figure S28.

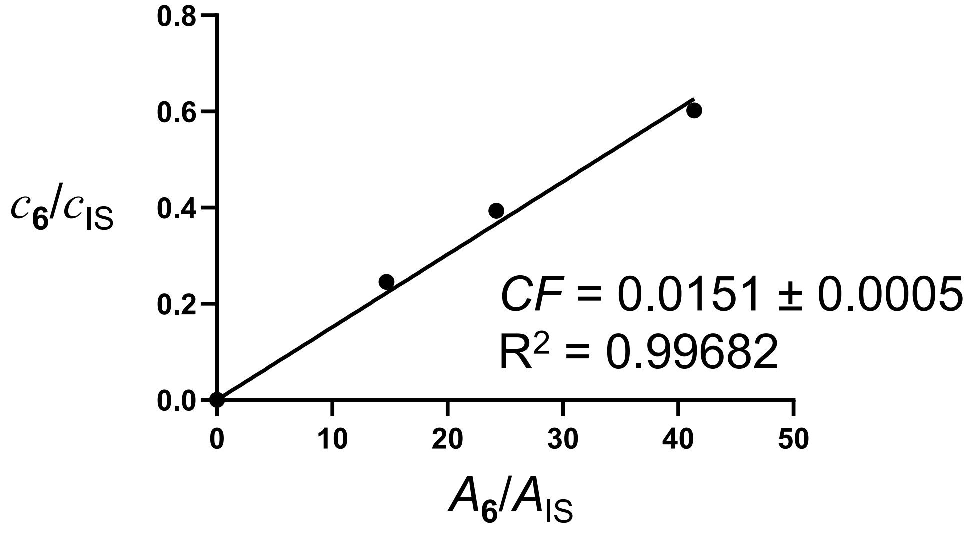

Figure S5. Calibration curve of product **6**. The peaks of *S*-**6** exceeded the detector's upper limit in the last two standard mixtures, so these two data points were excluded from the calibration curve. HPLC traces are shown in Figure S28.

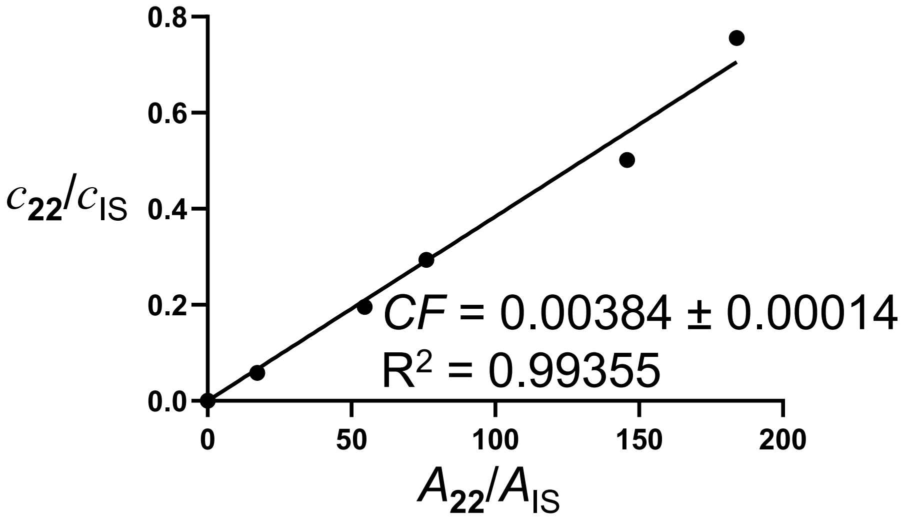

Figure S6. Calibration curve of substrate **22**. HPLC traces are shown in Figure S29.

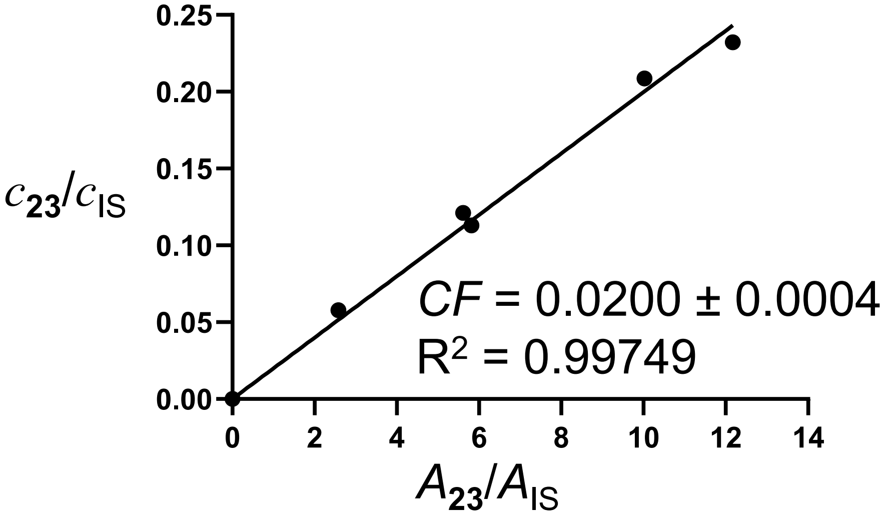

Figure S7. Calibration curve of product **23**. HPLC traces are shown in Figure S29.

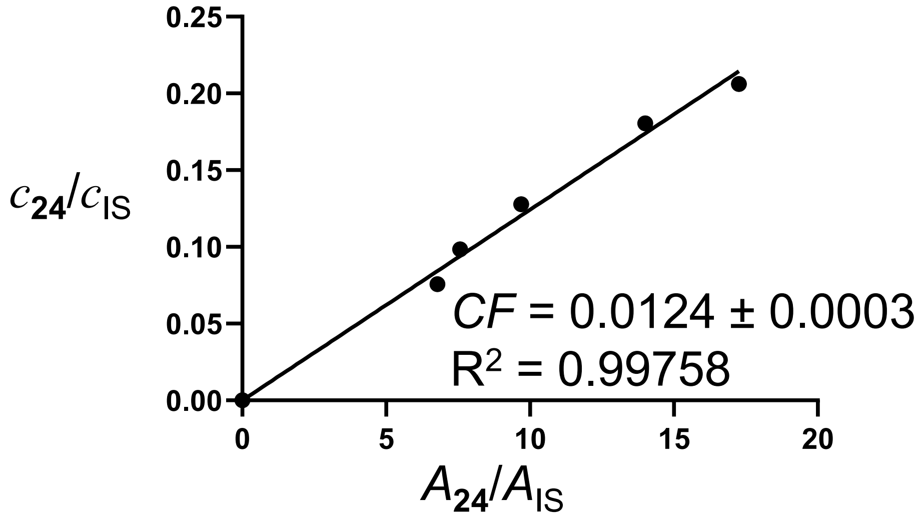

Figure S8. Calibration curve of product **24**. HPLC traces are shown in Figure S29.

4. Electric-Field Catalysis

4.1. Electromicrofluidic Reactor Configuration

Flow electrochemical experiments were performed using a setup similar to those described in reference[S1] using a stand-alone Vapourtec Ion Electrochemical Reactor, with an Aim-TTi EX354RD Dual Power Supply from Thurlbym Thandar Instruments Ltd. Chemyx Fusion 100 Touch Syringe Pumps were used in the flow set-ups. Electrode materials employed were rigid graphite foil (Gr, Goodfellow, 99.95%, 1.0 mm thickness) and platinum foil (Pt, Goodfellow, 99.99%, 0.1 mm thickness). The electrodes (5 x 5 cm^2) were separated by a 0.25 mm FEP spacer, resulting in a reactor volume of 0.3 mL, with an exposed electrode surface area of 12 cm^2.

The graphite plates were polished using a Presi Le Cube polishing machine, in sequential steps: 5 minutes with 6 μm abrasive paper and diamond suspension, followed by 3 minutes with 3 μm, 3 minutes with 1 μm, and finally 5 minutes with 1/4 μm particles.

After a series of reactions were completed, the system was flushed with DMSO (5.0 mL) followed by MeOH (5.0 mL) at a flow rate of 0.1 mL/min. The electrodes and FEP spacer were then cleaned with acetone. The graphite electrodes were further immersed in DMSO

overnight, followed by sonication in DMSO and DMF for 30 minutes each. Prior to reuse, the electrodes were repolished to restore surface quality.

4.2. General Procedure for Electric-field Catalysis

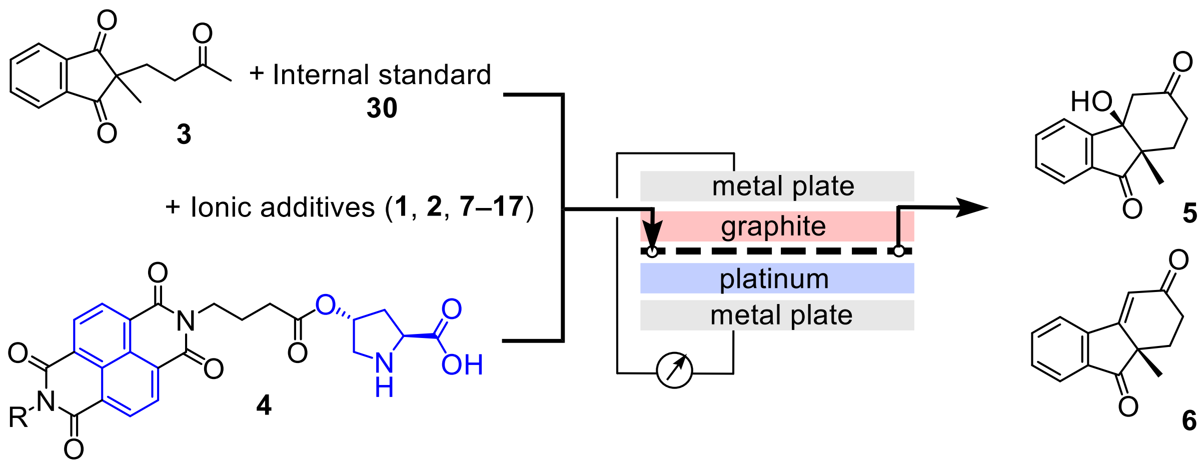

Scheme S6. Microfluidic electric-field catalysis of Robinson annulation

General procedure of EFC. Electrolyte solutions in DMSO were prepared as described below and used as solvents. A solution containing substrate **3** (100 mM) and the internal standard **30** (100 mM) was loaded in one syringe, and a solution of catalyst **4** (50 mM) was loaded in a separate syringe. The two solutions were mixed using a T-mixer and infused at 5 μL/min into the electrochemical reactor under constant voltage. The first one and a half reactor volume (0.45 mL) was disposed to ensure that a steady state of the system had been reached. After collecting for a defined period, the reaction mixture (0.1 mL) was diluted in EtOAc (0.3 mL), passed through a mini silica gel column to remove polar components including the catalyst and electrolytes, and then washed with brine (0.5 mL). The organic phase was collected and analyzed by HPLC.

Procedures for preparing electrolyte solutions.

Na/**2**: SDS (2.88 mg, 0.0100 mmol) was suspended in DMSO (10.0 mL) and dissolved upon sonication for 10 min. The concentration was 1.00 mM.

1/**2**: **1**·Cl (1.93 mg) was suspended in DMSO (9.80 mL) and dissolved upon sonication for 10 min. A solution of SDS (50.0 mM, 0.20 mL) in DMSO was added. The final concentrations of each component were as follows: 1.00 mM **1** (based on monomer unit concentration), 1.00 mM **2**.

1/Cl: **1**·Cl (1.93 mg) was dissolved in DMSO (10.0 mL). The concentration, calculated based on the arginine monomer units, is 1.00 mM.

1/**14**: The solution was prepared following the same procedure as that of the **1**/**2** solution, with SDS replaced by AOT.

1/*M*-**15**: *M*-**15** was used as sodium salts, prepared by neutralizing *M*-BINOL phosphoric acid (34.8 mg, 0.100 mmol) in DMSO (2.00 mL) with 1 M NaOH (0.10 mL), followed by lyophilization of the diluted solution (with 5 mL water). The sodium salt (3.70 mg, 0.100 mmol) was dissolved in DMSO (50 mL). **1**·Cl (1.93 mg) was suspended in DMSO (5.00 mL), and a solution of *M*-**15** sodium salt (5.00 mL) was added. The suspension was dissolved after sonication for 10 min. The final concentrations of each component were as follows: 1.00 mM **1** (based on monomer unit concentration), 1.00 mM *M*-**15**.

1/*P*-**15**: The solution was prepared following the same procedure as that of the **1**/*M*-**15** solution, with *M*-BINOL phosphoric acid replaced by *P*-BINOL phosphoric acid.

1/**17**: The solution was prepared following the same procedure as that of the **1**/**2** solution, with SDS replaced by $Na_2B_{12}Br_{12}$. The concentration of **17** was 0.500 mM to account for its divalent anionic nature.

1/**16**: The solution was prepared following the same procedure as that of the **1**/*M*-**15** solution, with *M*-BINOL phosphoric acid replaced by 1-pyrenebutyric acid.

10/**16**: The solution was prepared following the same procedure as that of the **1**/*M*-**15** solution, with *M*-BINOL phosphoric acid replaced by 1-pyrenebutyric acid and pR replaced by hexa-D-arginine. The concentration of **10** was 0.167 mM, corresponding to its six arginine residues per molecule.

13/**2**: The solution was prepared following the same procedure as that of the **1**/**2** solution, with pR replaced by a RW peptide [$(RWWRRWR)_3$]. The concentration of **13** was 0.083 mM, corresponding to its twelve arginine residues per molecule.

11/**2**: The solution was prepared following the same procedure as that of the **1**/**2** solution, with **1**·Cl replaced by **11**·Cl.

12/Cl: The solution was prepared following the same procedure as that of the **1**/Cl solution, with **1**·Cl replaced by **12**·Cl.

7/Cl: TBACl (2.78 mg, 0.0100 mmol) was dissolved in DMSO (10.0 mL). The concentration was 1.00 mM.

8/**2**: **8**·Cl (4.51 mg, 0.0100 mmol) was dissolved in DMSO (9.80 mL). A solution of SDS (50.0 mM, 0.20 mL) in DMSO was added. The final concentrations of each component were as follows: 1.00 mM **8**, 1.00 mM **2**.

9/**2**: The solution was prepared following the same procedure as that of the **8**/**2** solution, with **8** replaced by **9**.

4.3. EFC in Intrinsic EDL

Table S1. OEEF catalyzed Robinson annulation of **3** in DMSO[a]

Entry	V [mV][b]	Conv. [%][c]	Yield [%][d]		*ee* [%][e]		η_m [%][f]	ee_m [%][g]
			**5**	**6**	**5**	**6**		
1	0	13	11	0.2	89	86	11	89
2	+10	15	12	0.2	91	87	12	91
3	+50	19	15	0.3	91	87	15	91
4	+100	30	21	0.5	93	87	22	93
5	+200	32	24	0.8	90	88	25	90
6	0	18	15	0.4	88	89	15	88
7	+10	21	17	0.4	88	89	17	88
8	+50	22	20	0.4	90	90	20	90
9	+100	30	25	0.9	91	93	26	91
10	+200	33	25	0.7	91	93	25	91
11	0	13	12	0.2	90	84	12	90
12	-10	19	15	0.2	90	78	16	90
13	-50	20	17	0.2	92	94	17	92
14	-100	22	16	0.2	93	91	17	93
15	-200	20	17	0.2	93	83	17	93

[a] Conditions: 5 μL·min^{-1} flowrate, DMSO, 50 mM, rt, catalyst **4** (50 mol%). [b] Potentials applied to the Pt electrode, relative to the Gr electrode. +: Gr cathode, Pt anode; –: Gr anode, Pt cathode. Throughout the entire experiment, the current reading remained at 0.00 A. [c] Conversion after one passage through the electromicrofluidic reactor. [d] Yields of each product after one passage through the electromicrofluidic reactor. [e] The *ee* value of each product after one passage through the electromicrofluidic reactor. [f] The combined yield after one passage through the electromicrofluidic reactor. [g] The combined *ee* value after one passage through the electromicrofluidic reactor.

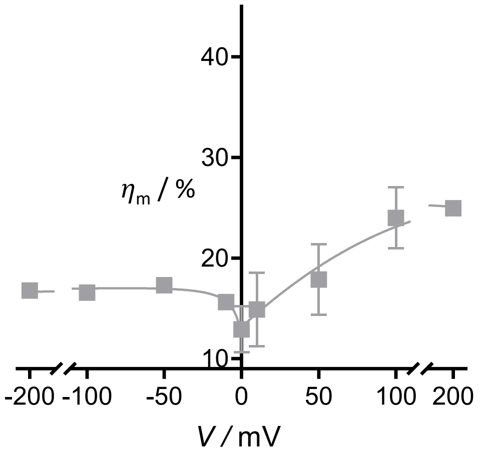

Figure S9. Yield (η_m) of products as a function of the applied voltage passing once through the electromicrofluidic reactor under electric fields. The error bars show the standard deviation of yields obtained from two independent measurements. Results are reported in Table S1 and HPLC traces in Figures S30-S32.

4.4. EFC in Engineered EDL

Table S2. OEEF catalyzed Robinson annulation of **3** in eEDL: control groups[a]

Entry	Additives	V [mV][b]	Conv. [%][c]	Yield [%][d]		*ee* [%][e]		η_m [%][f]	ee_m [%][g]
				**5**	**6**	**5**	**6**		
1	Na/**2**	0	28	19	0.4	91	95	19	91
2	Na/**2**	10	24	24	0.6	91	88	25	91
3	Na/**2**	50	29	26	0.5	91	88	27	91
4	Na/**2**	100	39	31	2.5	92	88	34	91
5	Na/**2**	200	41	32	2.4	89	89	34	89
6	**1**/Cl	0	16	14	0.6	93	83	14	93
7	**1**/Cl	10	43	31	1.2	93	81	33	92
8	**1**/Cl	50	52	36	1.5	92	83	37	92
9	**1**/Cl	100	50	36	2.0	90	82	38	90
10	**1**/Cl	200	50	35	2.0	89	81	37	89
11	**1/2**	0	22	14	0.7	92	59	15	90
12	**1/2**	10	57	31	1.8	93	78	33	92
13	**1/2**	50	53	37	2.3	91	79	39	90
14	**1/2**	100	53	36	2.4	91	79	38	90
15	**1/2**	200	48	34	2.6	90	80	37	89

[a] Conditions: Gr cathode, Pt anode, 5 $\mu L \cdot min^{-1}$ flowrate, DMSO, 50 mM, rt, catalyst **4** (50 mol%). The concentration of additives is 1.00 mM. For pR, the concentration is reported based on the number of monomer units, corresponding to the number of positive charges. [b] Throughout the entire experiment, the current reading remained at 0.00 A. [c] Conversion after one passage through the electromicrofluidic reactor. [d] Yields of products after one passage through the electromicrofluidic reactor. [e] The *ee* value of each product after one passage through the electromicrofluidic reactor. [f] The combined yield after one passage through the electromicrofluidic reactor. [g] The combined *ee* value after one passage through the electromicrofluidic reactor.

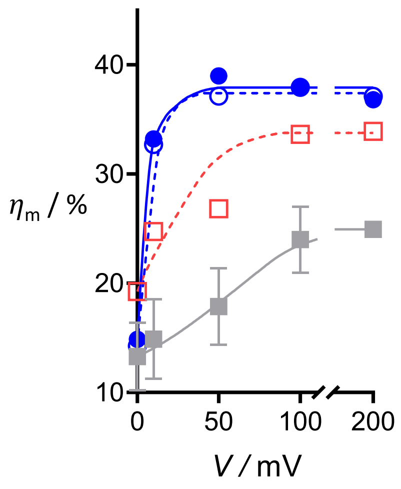

Figure S10. Yield (η_m) of products in intrinsic EDL (grey filled squares), in EDL formed by **2** (red empty squares), in EDL formed by **1** (blue empty circles), and in EDL formed by **1** and **2** (blue filled circles) as a function of the applied voltage passing once through the electromicrofluidic reactor. Results are reported in Table S2 and HPLC traces in Figures S30-S31, S33-S35.

Table S3. OEEF catalyzed Robinson annulation of **3** in eEDL: pR with various anions[a]

Entry	Additives	*V* [mV][b]	Conv. [%][c]	Yield [%][d] **5**	Yield [%][d] **6**	*ee* [%][e] **5**	*ee* [%][e] **6**	η_m [%][f]	ee_m [%][g]
1	**1/14**	0	21	15	0.7	87	79	16	86
2	**1/14**	10	39	28	1.1	92	84	30	92
3	**1/14**	50	45	33	1.7	92	87	35	91
4	**1/14**	100	46	33	2.2	90	86	35	90
5	**1/14**	200	36	33	2.6	88	87	36	88
6	**1/*M*-15**[h]	0	27	19	0.5	83	82	20	83
7	**1/*M*-15**	10	44	32	1.0	88	78	33	88
8	**1/*M*-15**	50	52	38	2.3	90	84	40	90
9	**1/*M*-15**	100	53	38	2.6	86	83	40	86
10	**1/*M*-15**	200	55	38	3.1	86	85	41	86
11	**1/*P*-15**[h]	0	17	14	0.5	88	88	15	88
12	**1/*P*-15**	10	47	37	1.7	91	87	39	90
13	**1/*P*-15**	50	50	39	2.7	88	86	42	88

14	**1**/*P*-**15**	100	49	38	2.5	89	86	40	89
15	**1**/*P*-**15**	200	49	37	2.7	88	87	40	88
16	**1/17**	0	19	16	0.4	88	90	16	88
17	**1/17**	10	30	29	0.7	89	94	29	89
18	**1/17**	50	49	40	3.1	87	91	43	87
19	**1/17**	100	54	40	2.9	86	87	43	86
20	**1/17**	200	54	40	3.8	84	87	44	85
21	**1/16**[h]	0	14	13	0.6	80	72	14	79
22	**1/16**	10	36	29	1.0	84	77	30	84
23	**1/16**	50	53	40	2.5	87	83	42	87
24	**1/16**	100	54	40	2.9	88	83	43	87
25	**1/16**	200	54	42	2.7	88	83	44	87
26	**1/16**	0	16	11	0.1	82	89	12	82
27	**1/16**	10	38	29	0.4	87	90	29	87
28	**1/16**	50	54	36	1.2	87	89	37	87
29	**1/16**	100	56	36	1.6	86	86	38	86
30	**1/16**	200	56	36	2.0	85	86	38	85
31	**1/16**	0	11	8	0.1	80	75	8	80
32	**1/16**	10	40	31	0.6	87	92	32	87
33	**1/16**	50	55	38	1.5	87	87	39	87
34	**1/16**	100	56	38	1.8	87	86	40	87
35	**1/16**	200	56	35	1.9	86	86	37	86

[a] Conditions: Gr cathode, Pt anode, 5 μL·min^{-1} flowrate, DMSO, 50 mM, rt, catalyst **4** (50 mol%). The concentration of additives is 1.00 mM. For polymers or multivalent ions, the concentration is reported based on the number of charges. [b] Throughout the entire experiment, the current reading remained at 0.00 A. [c] Conversion after one passage through the electromicrofluidic reactor. [d] Yields of products after one passage through the electromicrofluidic reactor. [e] The *ee* value of each product after one passage through the electromicrofluidic reactor. [f] The combined yield after one passage through the electromicrofluidic reactor. [g] The combined *ee* value after one passage through the

electromicrofluidic reactor. [h] *M*-**15**, *P*-**15**, and **16** were used as sodium salts as described above.

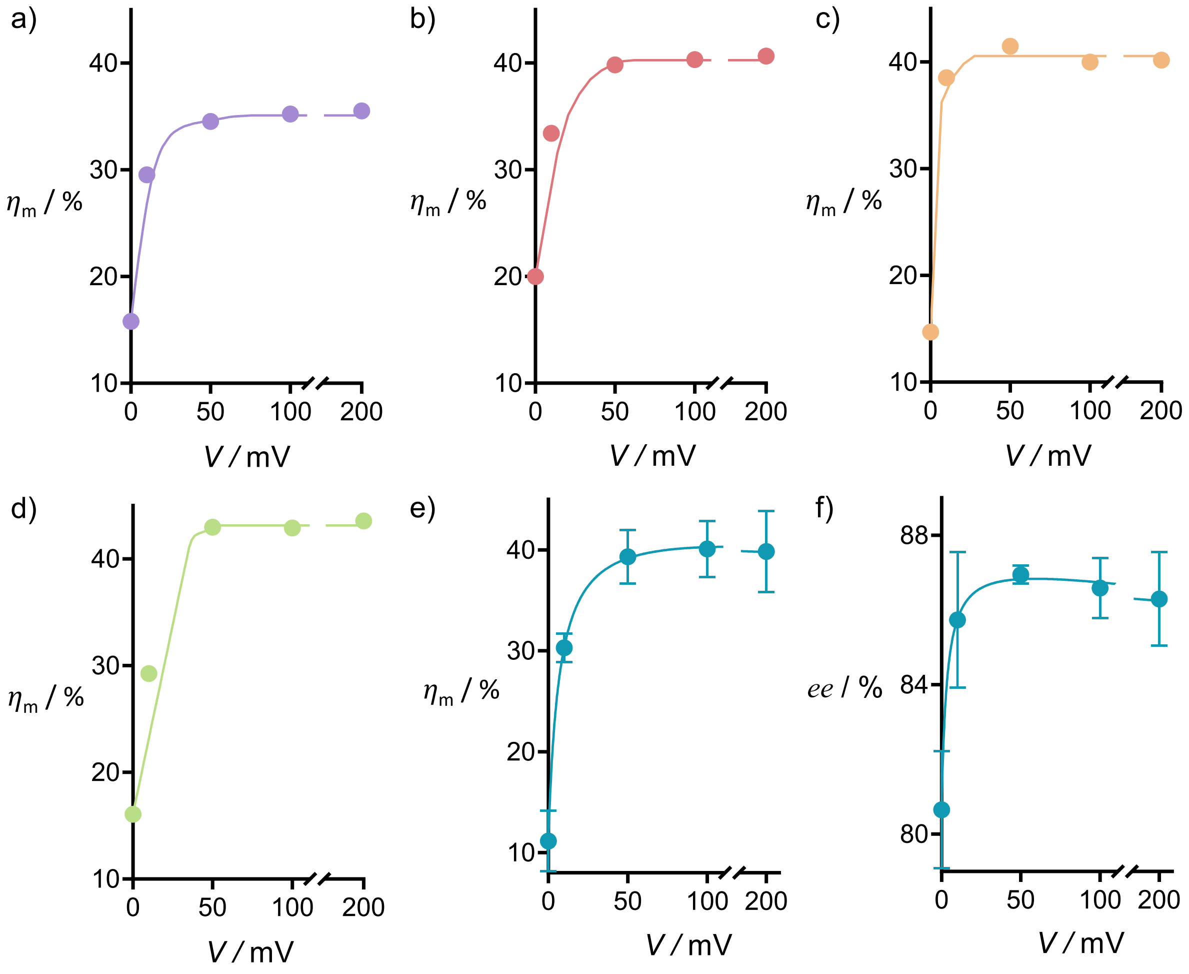

Figure S11. a-e) Yield (η_m) of products in EDL formed by a) **1**/**14**; b) **1**/*M*-**15**; c) **1**/*P*-**15**; d) **1**/**17**; e) **1**/**16** as a function of the applied voltage passing once through the electromicrofluidic reactor. f) The *ee* values of products in EDL formed **1**/**16** as a function of the applied voltage passing once through the electromicrofluidic reactor. The error bars show the standard deviation of yields obtained from three independent measurements. Results are reported in Table S3 and HPLC traces in Figures S36-S42.

Table S4. OEEF catalyzed Robinson annulation of **3** in eEDL: other guanidinium type cations[a]

Entry	Additives	V [mV][b]	Conv. [%][c]	Yield [%][d] **5**	Yield [%][d] **6**	*ee* [%][e] **5**	*ee* [%][e] **6**	η_{m} [%][f]	ee_{m} [%][g]
1	**10/16**[h]	0	15	13	0.4	87	89	14	87
2	**10/16**	10	45	34	2.1	89	92	36	89
3	**10/16**	50	51	37	2.6	86	89	39	86
4	**10/16**	100	55	38	3.3	84	87	42	84
5	**10/16**	200	59	36	3.5	82	87	39	82
6	**13/2**	0	34	32	2.0	92	96	34	92
7	**13/2**	10	46	39	2.9	92	90	42	91
8	**13/2**	50	48	39	3.6	89	89	42	89
9	**13/2**	100	48	39	3.9	87	87	43	87
10	**13/2**	200	48	37	3.5	87	87	41	87
11	**11/2**	0	24	21	0.9	91	80	22	91
12	**11/2**	10	48	34	2.0	91	73	36	90
13	**11/2**	50	53	37	2.7	89	76	40	88
14	**11/2**	100	54	40	3.2	89	77	43	89
15	**11/2**	200	54	38	3.4	88	79	42	87
16	**12**/Cl	0	21	18	0.4	92	82	19	92
17	**12**/Cl	10	31	28	0.6	92	87	28	92
18	**12**/Cl	50	48	36	1.6	92	89	38	92
19	**12**/Cl	100	49	36	1.4	90	87	37	90
20	**12**/Cl	200	48	37	1.9	90	87	39	90

[a] Conditions: Gr cathode, Pt anode, 5 μL·min^{-1} flowrate, DMSO, 50 mM, rt, catalyst **4** (50 mol%). The concentration of additives is 1.00 mM. For polymers or multivalent ions, the concentration is reported based on the number of charges. [b] Throughout the entire experiment, the current reading remained at 0.00 A. [c] Conversion after one passage through the electromicrofluidic reactor. [d] Yields of products after one passage through the electromicrofluidic reactor. [e] The *ee* value of each product after one passage through the

electromicrofluidic reactor. [f] The combined yield after one passage through the electromicrofluidic reactor. [g] The combined *ee* value after one passage through the electromicrofluidic reactor. [h] **16** was used as a sodium salt as described above.

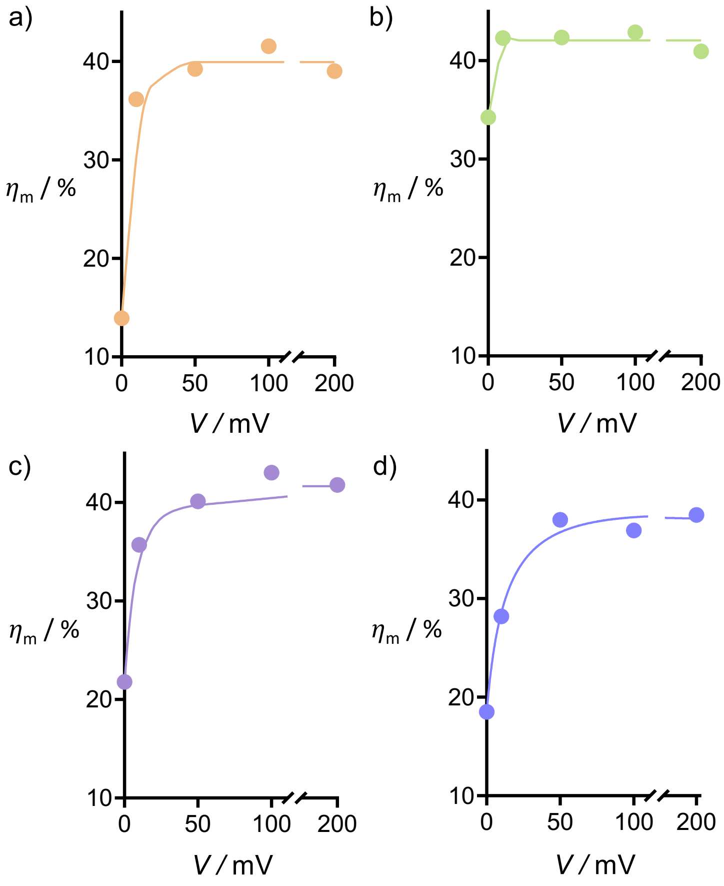

Figure S12. Yield (η_m) of products in EDL formed by a) **10**/**16**; b) **13**/**2**; c) **11**/**2**; d) **12**/Cl as a function of the applied voltage passing once through the electromicrofluidic reactor. Results are reported in Table S4 and HPLC traces in Figures S43-S46.

Table S5. OEEF catalyzed Robinson annulation of **3** in eEDL: SDS with various cations[a]

Entry	Additives	V [mV][b]	Conv. [%][c]	Yield [%][d]		*ee* [%][e]		η_m [%][f]	ee_m [%][g]
				**5**	**6**	**5**	**6**		
1	**7**/Cl	0	21	18	0.3	92	74	18	92
2	**7**/Cl	10	31	21	0.7	93	73	22	92
3	**7**/Cl	50	31	22	0.9	92	73	23	91
4	**7**/Cl	100	32	22	0.9	92	86	23	92
5	**7**/Cl	200	30	22	1.0	91	79	23	90
6	**8**/**2**	0	21	19	1.1	87	64	20	86
7	**8**/**2**	10	30	24	1.2	93	71	25	92
8	**8**/**2**	50	29	25	1.2	93	71	26	92
9	**8**/**2**	100	33	24	1.2	91	72	25	90
10	**8**/**2**	200	34	25	1.3	93	72	27	92
11	**9**/**2**	0	30	27	1.1	94	81	28	94
12	**9**/**2**	10	40	34	1.7	93	79	35	92
13	**9**/**2**	50	42	35	2.0	91	80	36	90
14	**9**/**2**	100	44	34	1.9	91	80	36	90
15	**9**/**2**	200	45	33	1.9	90	82	35	90

[a] Conditions: Gr cathode, Pt anode, 5 $\mu L\cdot min^{-1}$ flowrate, DMSO, 50 mM, rt, catalyst **4** (50 mol%). The concentration of additives is 1.00 mM. [b] Throughout the entire experiment, the current reading remained at 0.00 A. [c] Conversion after one passage through the electromicrofluidic reactor. [d] Yields of products after one passage through the electromicrofluidic reactor. [e] The *ee* value of each product after one passage through the electromicrofluidic reactor. [f] The combined yield after one passage through the electromicrofluidic reactor. [g] The combined *ee* value after one passage through the electromicrofluidic reactor.

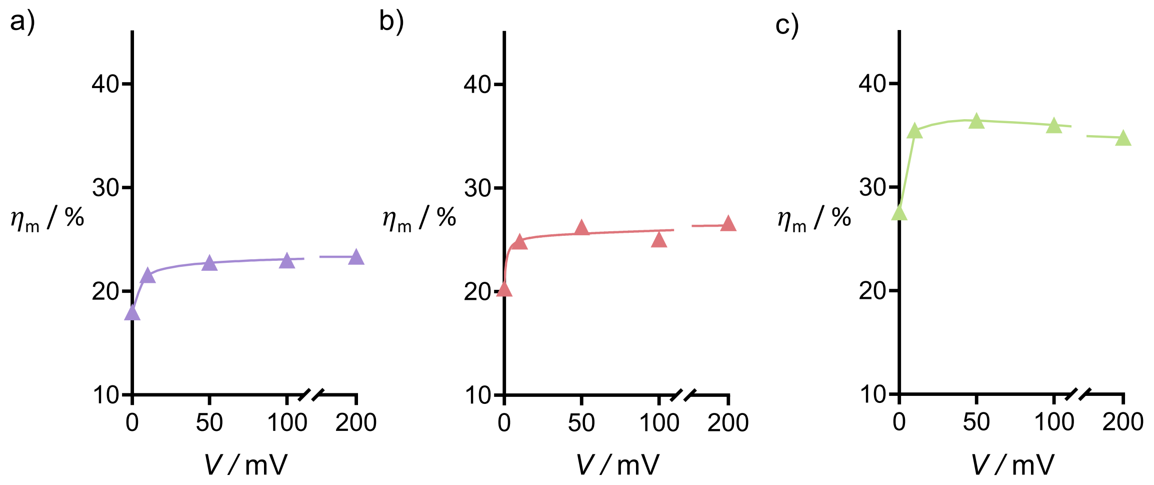

Figure S13. Yield (η_m) of products in EDL formed by a) **7**/Cl; b) **8**/**2**; c) **9**/**2** as a function of the applied voltage passing once through the electromicrofluidic reactor. Results are reported in Table S5 and HPLC traces in Figures S47-S49.

Table S6. OEEF catalyzed Robinson annulation of **22** in DMSO and eEDL [a]

Entry	Additives	*V* [mV][b]	Conv. [%][c]	Yield [%][d]		*ee* [%][e]		η_m [%][f]	ee_m [%][g]
				**23**	**24**	**23**	**24**		
1	-	0	22	20	0.2	96	80	20	96
2	-	10	28	24	0.3	96	93	24	96
3	-	50	30	28	0.3	96	83	28	96
4	-	100	33	30	0.4	96	84	31	96
5	-	200	34	27	0.3	96	80	28	96
6	**1**/**2**	0	20	19	0.5	96	-5	20	93
7	**1**/**2**	10	45	41	1.1	96	45	42	94
8	**1**/**2**	50	70	51	2.6	93	71	53	92
9	**1**/**2**	100	68	51	2.4	94	68	53	93
10	**1**/**2**	200	73	47	2.4	93	73	50	92

[a] Conditions: Gr cathode, Pt anode, 5 µL·min^{-1} flowrate, DMSO, 50 mM, rt, catalyst **4** (50 mol%). The concentration of additives is 1.00 mM. For pR **1**, the concentration is reported based on the number of monomer units, corresponding to the number of positive charges. [b] Throughout the entire experiment, the current reading remained at 0.00 A. [c] Conversion after

one passage through the electromicrofluidic reactor. [d] Yields of products after one passage through the electromicrofluidic reactor. [e] The *ee* value of each product after one passage through the electromicrofluidic reactor. [f] The combined yield after one passage through the electromicrofluidic reactor. [g] The combined *ee* value after one passage through the electromicrofluidic reactor.

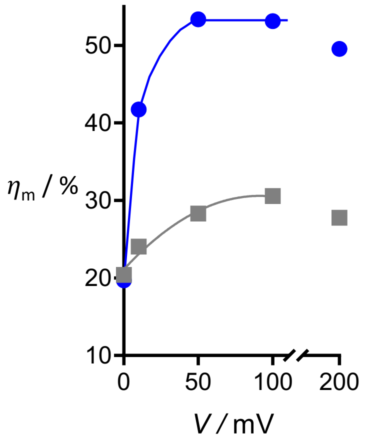

Figure S14. Yield (η_m) of products in intrinsic EDL (grey squares) and EDL formed by **1**/**2** (blue circles) as a function of the applied voltage passing once through the electromicrofluidic reactor. Results are reported in Table S6 and HPLC traces in Figures S50 and S51.

4.5. TEMPO Control

A DMSO solution of **1**/**2** (1 mM each) was used as a solvent. A solution of substrate **3** (100 mM), the internal standard **30** (100 mM), and TEMPO (100 mM) was loaded in one syringe, and a solution of catalyst **4** (50 mM) was loaded in a separate syringe. The flow electrochemical experiment was performed following the general procedure.

Table S7. OEEF catalyzed Robinson annulation of **3** in eEDL: TEMPO control[a]

Entry	Additives	V [mV][b]	Conv. [%][c]	Yield [%][d] **5**	Yield [%][d] **6**	*ee* [%][e] **5**	*ee* [%][e] **6**	η_m [%][f]	ee_m [%][g]
1	**1/2**, TEMPO	0	16	9	0.4	90	94	9	90
2	**1/2**, TEMPO	10	59	36	1.7	88	86	38	88
3	**1/2**, TEMPO	50	55	39	2.0	87	88	41	87
4	**1/2**, TEMPO	100	57	38	1.8	88	87	40	88
5	**1/2**, TEMPO	200	57	37	1.9	88	86	39	88

[a] Conditions: Gr cathode, Pt anode, 5 µL·min^{-1} flowrate, DMSO, 50 mM, rt, catalyst **4** (50 mol%), and 1 equivalent of TEMPO. The concentration of **1** and **2** is 1.00 mM. For pR **1**, the concentration is reported based on the number of monomer units, corresponding to the number of positive charges. [b] Throughout the entire experiment, the current reading remained at 0.00 A. [c] Conversion after one passage through the electromicrofluidic reactor. [d] Yields of products after one passage through the electromicrofluidic reactor. [e] The *ee* value of each product after one passage through the electromicrofluidic reactor. [e] The combined yield after one passage through the electromicrofluidic reactor. [f] The combined *ee* value after one passage through the electromicrofluidic reactor.

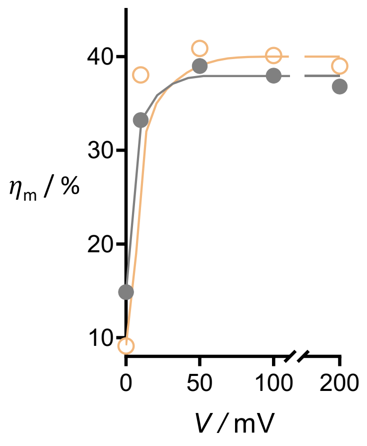

Figure S15. Yield (η_m) of products in EDL formed by **1/2** with (orange empty circles) or without (grey filled circles) 1 equivalent of TEMPO as a function of the applied voltage passing

once through the electromicrofluidic reactor. Results are reported in Tables S2 and S7 and HPLC traces in Figures S35 and S52.

4.6. EFC with Alternating Current

The alternating current experiments were performed using a TDK·Lambda GAC-PRO-03 AC+DC Power Source. Except for the use of this power supply, all other aspects of the procedure were identical to those described in the general procedure.

Table S8. OEEF catalyzed Robinson annulation of **3** in eEDL: alternative current mode[a]

Entry	*f* [Hz]	*V* [mV]	*I* [A]	Conv. [%][b]	Yield [%][c] **5**	Yield [%][c] **6**	*ee* [%][d] **5**	*ee* [%][d] **6**	η_m [%][e]	ee_m [%][f]
1	DC[g]	50	0.00	53	37	2.2	91	79	39	90
2	16	50	0.00	48	35	1.7	87	94	36	87
3	200	140[h]	±0.04	49	35	2.2	86	93	37	86

[a] Conditions: Gr cathode, Pt anode, 5 μL·min^{-1} flowrate, DMSO, 50 mM, rt, catalyst **4** (50 mol%), and 1.00 mM **1**/**2** were applied. For pR, the concentration is reported based on the number of monomer units, corresponding to the number of positive charges. [b] Conversion after one passage through the electromicrofluidic reactor. [c] Yields of products after one passage through the electromicrofluidic reactor. [d] The *ee* value of each product after one passage through the electromicrofluidic reactor. [e] The combined yield after one passage through the electromicrofluidic reactor. [f] The combined *ee* value after one passage through the electromicrofluidic reactor. [g] Directing current mode, see Table S3, entry 13. [h] The minimum voltage that can be set when the frequency was 200 Hz.

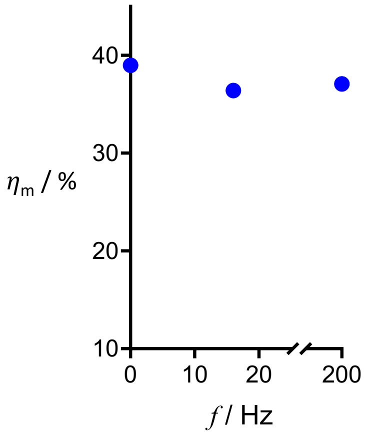

Figure S16. Yield (η_m) of products in EDL formed by **1/2** as a function of the applied voltage frequency passing once through the electromicrofluidic reactor. Results are reported in Tables S2 and S8 and HPLC traces in Figures S35 and S53.

4.7. EFC with Different Flowrates

A DMSO solution containing substrate **22** (100 mM), the internal standard **30** (100 mM), and electrolytes **1**/**16** (1.00 mM) was loaded in one syringe, while a DMSO solution of catalyst **4** (30 mM) and electrolytes **1**/**16** (1.00 mM) was loaded in a separate syringe. The two solutions were mixed using a T-mixer and infused at different flowrates into the electrochemical reactor under constant voltage (50 mV). The first one and a half reactor volume (0.45 mL) was disposed to ensure that a steady state of the system had been reached. After collecting for a defined period, the reaction mixture (0.1 mL) was diluted in EtOAc (0.3 mL), passed through a mini silica gel column to remove polar components including the catalyst and electrolytes, and then washed with brine (0.5 mL). The organic phase was collected and analyzed by HPLC.

Table S9. OEEF catalyzed Robinson annulation of **22** in eEDL: different flowrate[a]

Entry	Flowrate [μL/min]	V [mV][b]	Conv. [%][c]	Yield [%][d] **23**	Yield [%][d] **24**	*ee* [%][e] **23**	*ee* [%][e] **24**	η_m [%][f]	ee_m [%][g]
1	10.0	50	38	30	0.4	94	82	30	94
2	5.0	50	48	43	1.3	94	88	44	94
3	2.0	50	62	54	2.0	93	81	56	93
4	1.0	50	73	53	3.3	94	78	56	93
5	0.5	50	79	53	3.7	94	74	57	93

[a] Conditions: Gr cathode, Pt anode, DMSO, 50 mM, rt, catalyst **4** (30 mol%), and 1.00 mM **1/16** were applied. For pR, the concentration is reported based on the number of monomer units, corresponding to the number of positive charges. [b] Throughout the entire experiment, the current reading remained at 0.00 A. [c] Conversion after one passage through the electromicrofluidic reactor. [d] Yields of products after one passage through the electromicrofluidic reactor. [e] The *ee* value of each product after one passage through the electromicrofluidic reactor. [f] The combined yield after one passage through the electromicrofluidic reactor. [g] The combined *ee* value after one passage through the electromicrofluidic reactor.

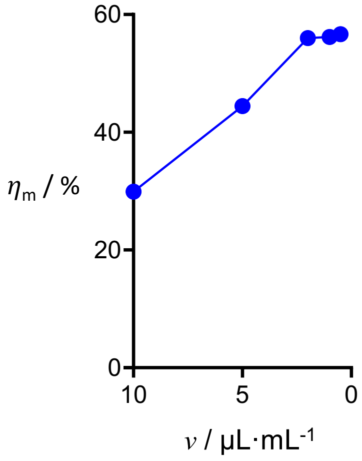

Figure S17. Yield (η_m) of products in EDL formed by **1/16** as a function of the flowrate passing once through the electromicrofluidic reactor using using **4** (30 mol%) as the catalyst. Results are reported in Table S9 and HPLC traces in Figure S54.

4.8. EFC with Different Electrodes

These experiments were performed using two graphite plates as electrodes. Except for the electrodes, all other aspects of the procedure were identical to those described in the general procedure.

Table S10. OEEF catalyzed Robinson annulation of **3** in eEDL: different electrodes[a]

Entry	*V* [mV]	Conv. [%][b]	Yield [%][c]		*ee* [%][d]		η_m [%][e]	ee_m [%][f]
			**5**	**6**	**5**	**6**		
1	0	24	15	0.3	90	88	16	90
2	10	30	24	0.4	93	89	24	93
3	50	37	33	0.8	92	79	34	91
4	100	34	33	0.8	93	83	33	93
5	200	40	35	1.5	92	87	37	92

[a] Conditions: Gr cathode, Gr anode, 5 $\mu L \cdot min^{-1}$ flowrate, DMSO, 50 mM, rt, catalyst **4** (50 mol%), and 1.00 mM **1**/**2** were applied. For pR, the concentration is reported based on the number of monomer units, corresponding to the number of positive charges. [b] Conversion after one passage through the electromicrofluidic reactor. [c] Yields of products after one passage through the electromicrofluidic reactor. [d] The *ee* value of each product after one passage through the electromicrofluidic reactor. [e] The combined yield after one passage through the electromicrofluidic reactor. [f] The combined *ee* value after one passage through the electromicrofluidic reactor.

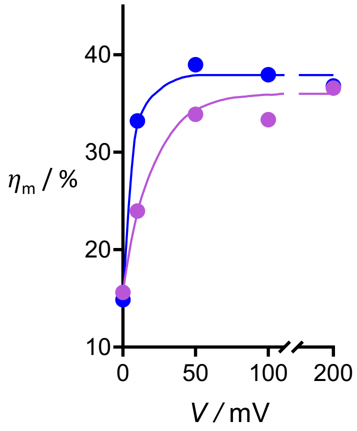

Figure S18. Yield (η_m) of products in EDL formed by **1/2** with Gr cathode and Pt anode (blue) or Gr cathode and Gr anode (violet), as a function of the applied voltage frequency passing once through the electromicrofluidic reactor. Results are reported in Tables S2 and S10 and HPLC traces in Figures S35 and S55.

5. Supplementary References

S1 M. Á. Gutiérrez López, R. Ali, M.-L.Tan, N. Sakai, T. Wirth, S. Matile, *Sci. Adv.,* 2023, **9**, eadj5502.

S2 K. Mori, T. Katoh, T. Suzuki, T. Noji, M. Yamanaka, T. Akiyama, *Angew. Chem. Int. Ed*. **2009**, *48*, 9652-9654.

S3 S. Lee, Y. Hua, A. H. Flood, *J. Org. Chem.* **2014**, *79*, 8383-8396.

S4 T. E. Kristensen, F. K. Hansen, T. Hansen, *Eur. J. Org. Chem.* **2009**, 387-395.

The original data can be found at: https://doi.org/10.5281/zenodo.17052869.

6. HPLC Chromatograms

6.1. Reference Compounds

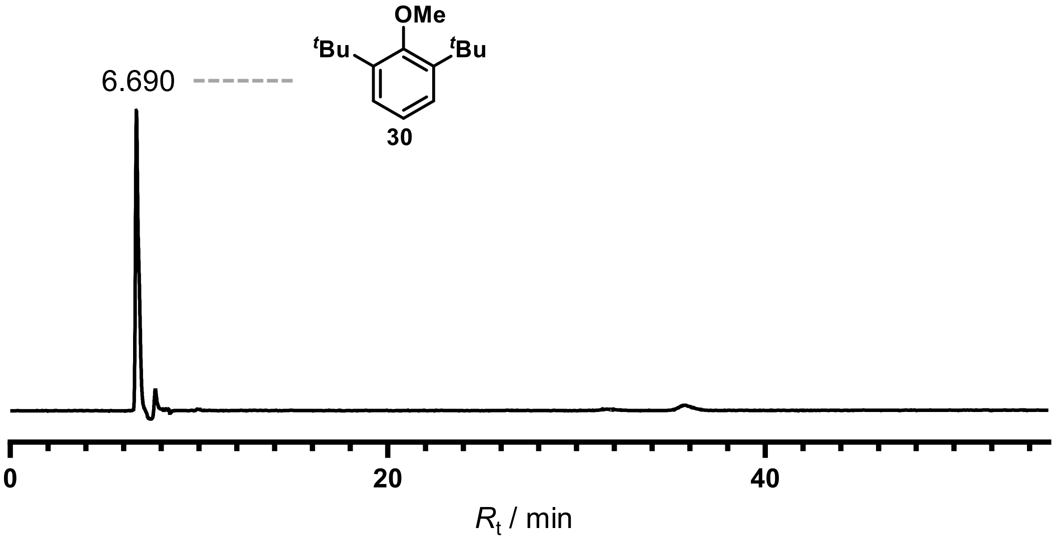

Figure S19. HPLC profile of the internal standard **30**. HPLC method: CHIRALPAK® ID (4.6 mm Ø x 25.0 cm *l*), 25 °C, 0.5 mL/min flow rate, Hexane/^{i}PrOH/EtOAc 18:1:1, detection at λ_{abs} = 270 nm.

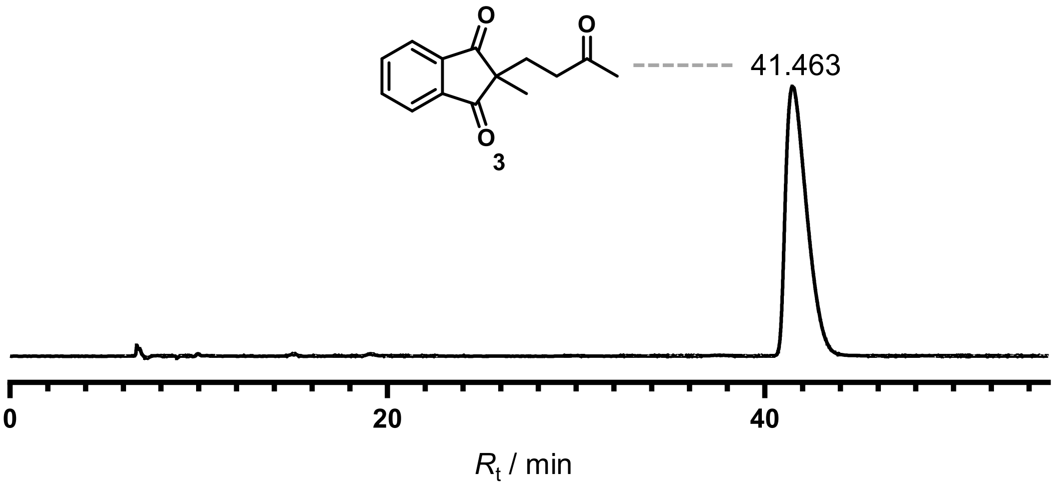

Figure S20. HPLC profile of substrate **3**. HPLC method: CHIRALPAK® ID (4.6 mm Ø x 25.0 cm *l*), 25 °C, 0.5 mL/min flow rate, Hexane/^{i}PrOH/EtOAc 18:1:1, detection at λ_{abs} = 270 nm.

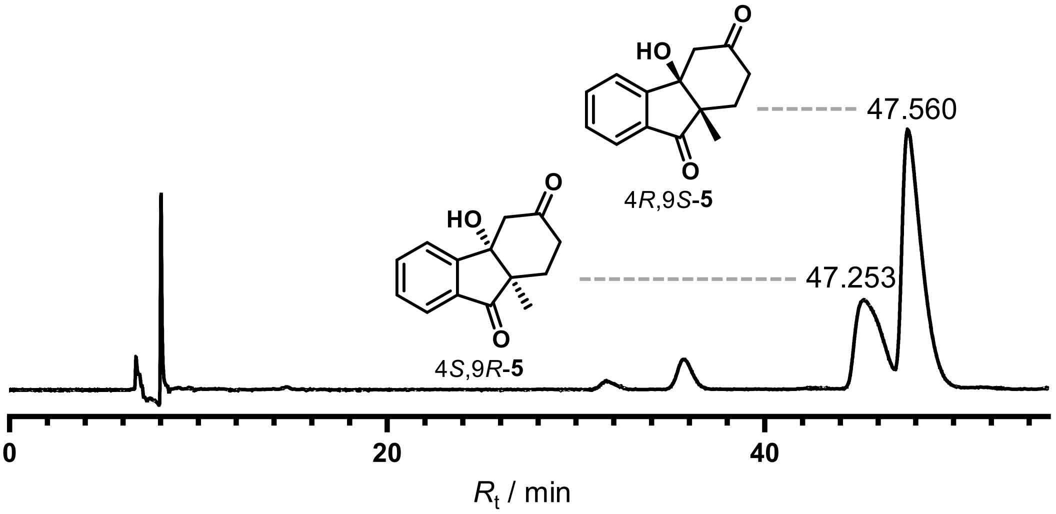

Figure S21. HPLC profile of product **5**, with product **6** (0.3%) as an impurity. HPLC method: CHIRALPAK® ID (4.6 mm Ø x 25.0 cm *l*), 25 °C, 0.5 mL/min flow rate, Hexane/^{i}PrOH/EtOAc 18:1:1, detection at λ_{abs} = 270 nm. The enantiomers were assigned by comparing the relative peak intensities with those reported in the literature.[S2]

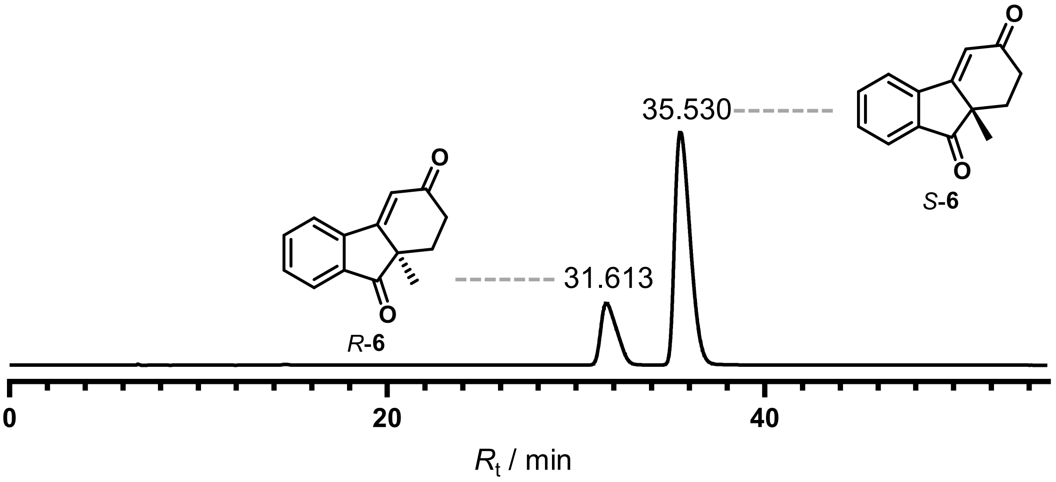

Figure S22. HPLC profile of product **6**. HPLC method: CHIRALPAK® ID (4.6 mm Ø x 25.0 cm *l*), 25 °C, 0.5 mL/min flow rate, Hexane/^{i}PrOH/EtOAc 18:1:1, detection at λ_{abs} = 270 nm. The enantiomers were assigned by comparing the relative peak intensities with those reported in the literature.[S2]

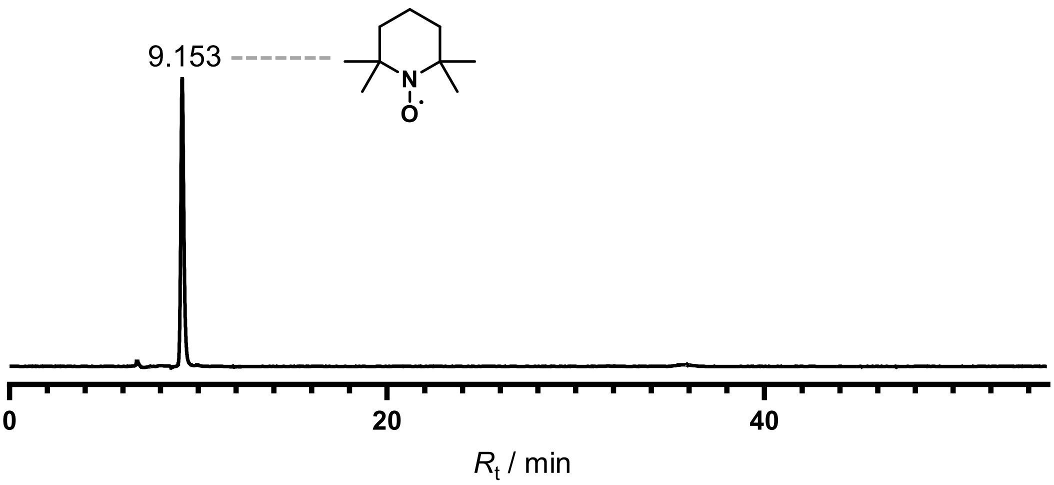

Figure S23. HPLC profile of TEMPO. HPLC method: CHIRALPAK® ID (4.6 mm Ø x 25.0 cm *l*), 25 °C, 0.5 mL/min flow rate, Hexane/^{i}PrOH/EtOAc 18:1:1, detection at λ_{abs} = 270 nm.

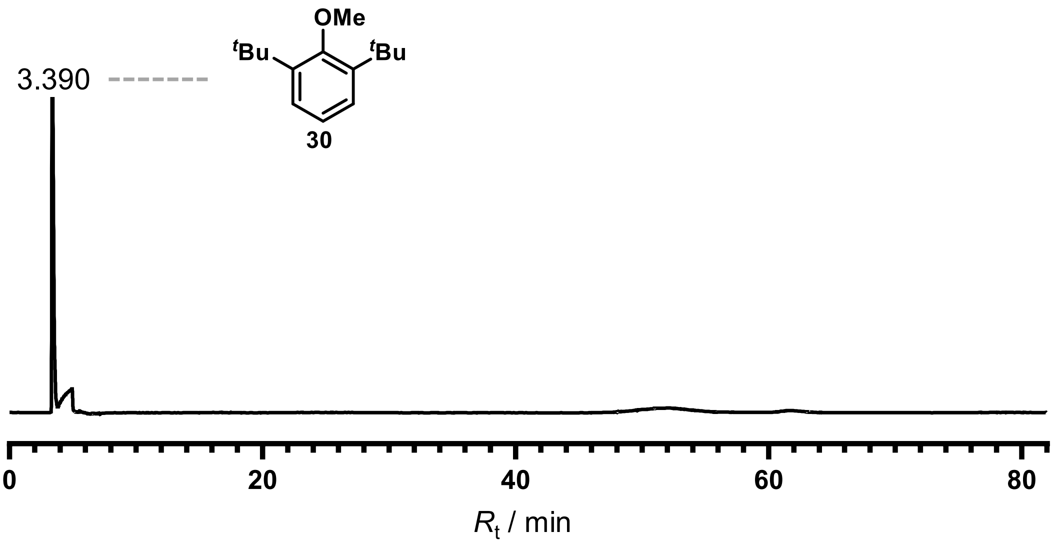

Figure S24. HPLC profile of the internal standard **30**. HPLC method: CHIRALPAK® ID (4.6 mm Ø x 25.0 cm *l*), 25 °C, 1.0 mL/min flow rate, Hexane/^{i}PrOH/EtOAc 188:7:5, detection at λ_{abs} = 257 nm.

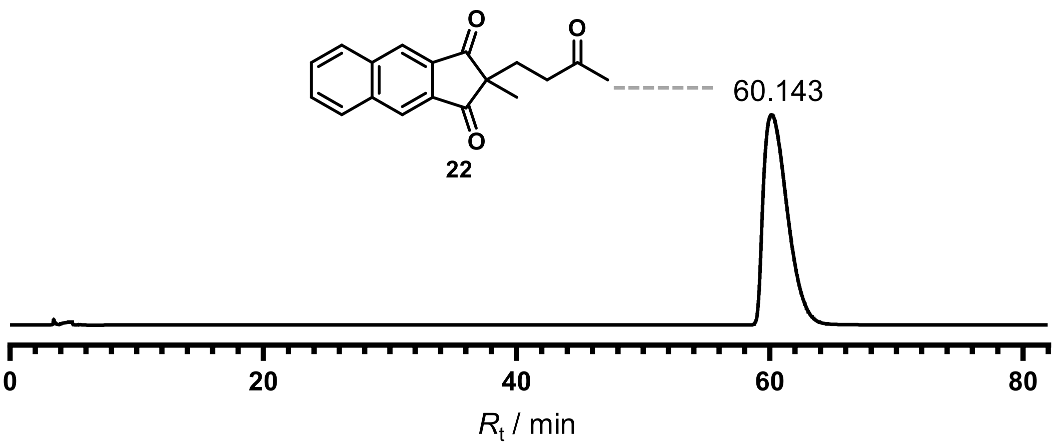

Figure S25. HPLC profile of substrate **22**. HPLC method: CHIRALPAK® ID (4.6 mm Ø x 25.0 cm *l*), 25 °C, 1.0 mL/min flow rate, Hexane/^{i}PrOH/EtOAc 188:7:5, detection at λ_{abs} = 257 nm.

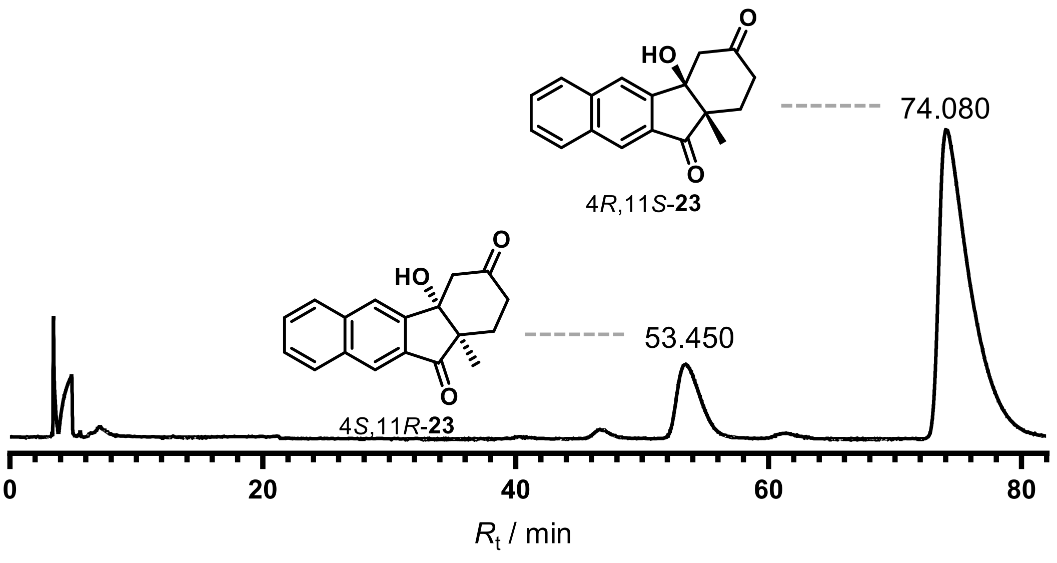

Figure S26. HPLC profile of product **23**, with substrate **22** (0.2%) and product **24** (1%) as impurities. HPLC method: CHIRALPAK® ID (4.6 mm Ø x 25.0 cm *l*), 25 °C, 1.0 mL/min flow rate, Hexane/^{i}PrOH/EtOAc 188:7:5, detection at λ_{abs} = 257 nm. The enantiomers were assigned by comparing the relative peak intensities with those reported in the literature.[S2]

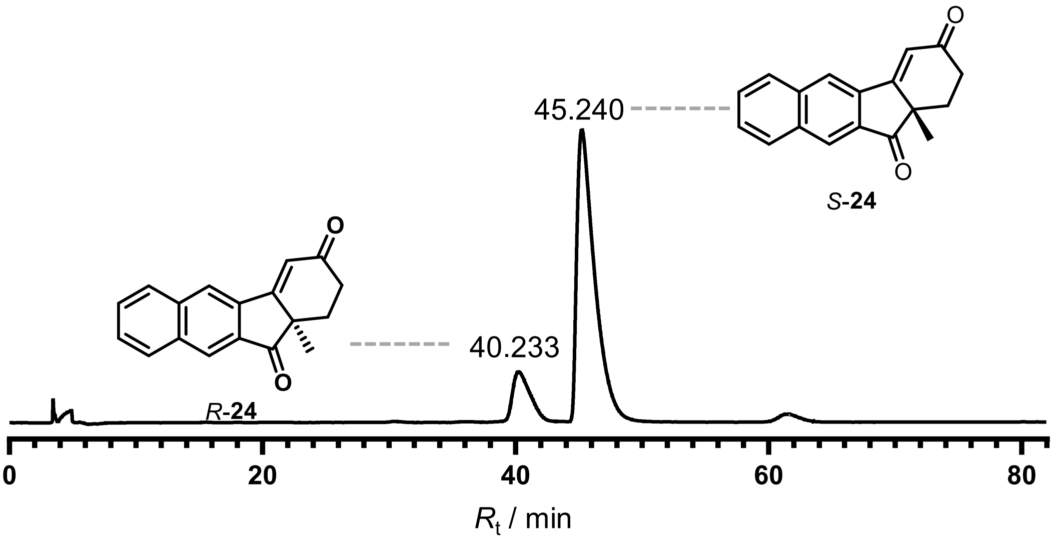

Figure S27. HPLC profile of product **24**, with substrate **22** (0.8%) as an impurity. HPLC method: CHIRALPAK® ID (4.6 mm Ø x 25.0 cm *l*), 25 °C, 1.0 mL/min flow rate, Hexane/^{i}PrOH/EtOAc 188:7:5, detection at λ_{abs} = 257 nm. The enantiomers were assigned by comparing the relative peak intensities with those reported in the literature.[S2]

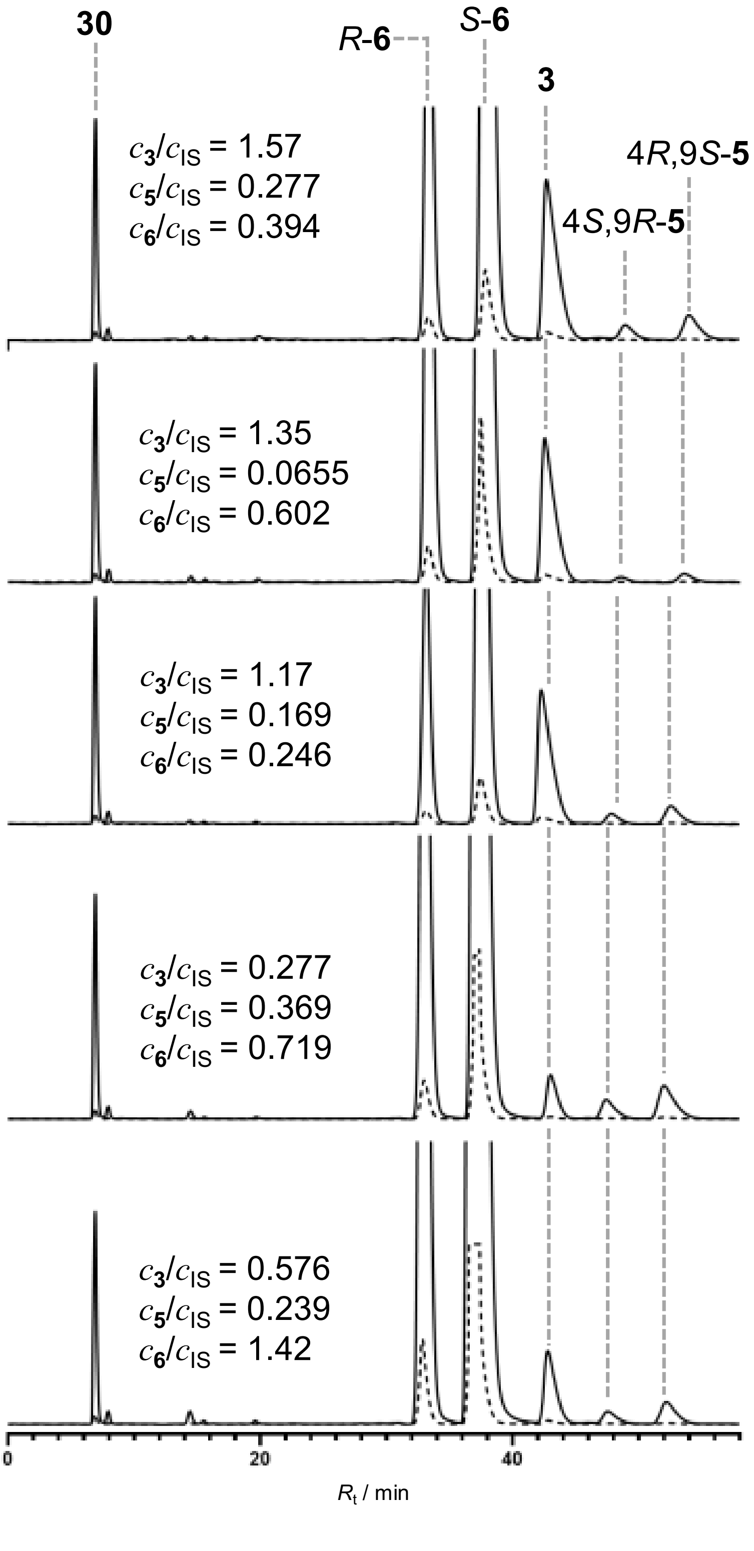

Figure S28. HPLC profiles showing the calibration of **3**, **5**, and **6** to the internal standard **30**. HPLC method was described in section 3.1. The absorption of **6** is significantly stronger than other components, so the intensities of the spectra were amplified by 20-fold (solid lines) to show all the peaks, while the original traces were shown as dotted lines.

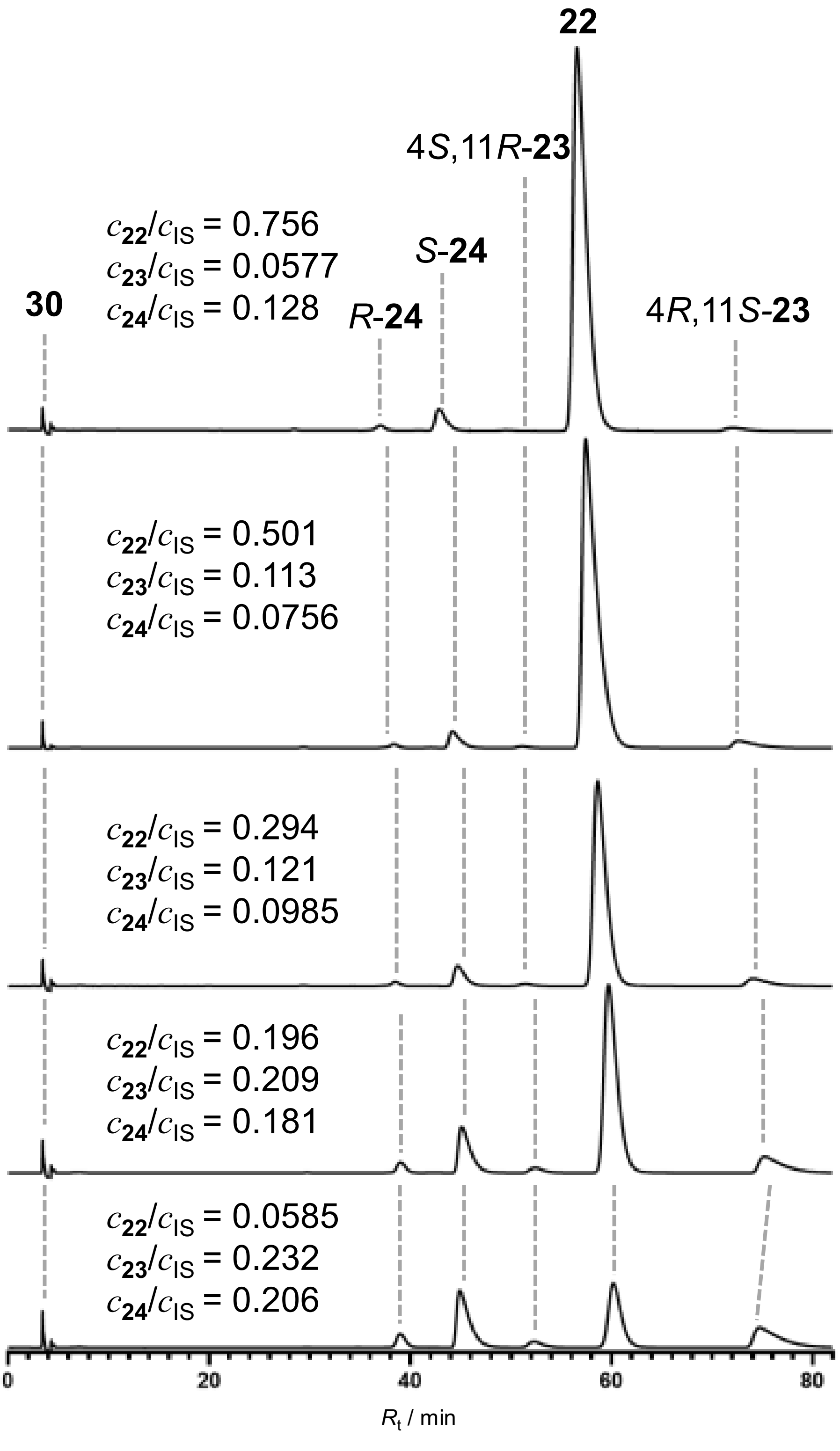

Figure S29. HPLC profiles showing the calibration of **22**, **23**, and **24** to the internal standard **30**. HPLC method was described in section 3.1.

6.2. Reaction Mixtures

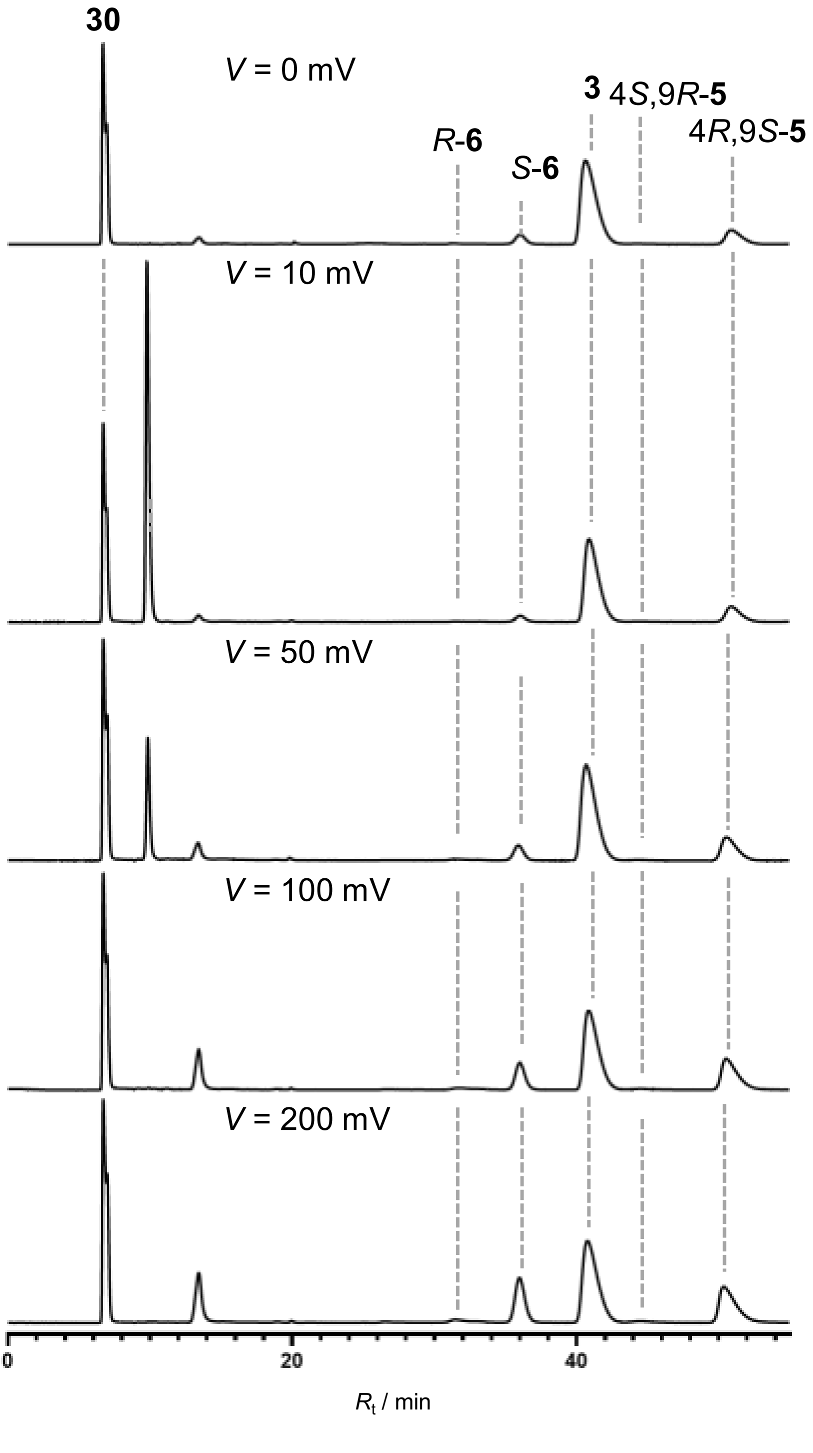

Figure S30. HPLC profiles showing the Robinson annulation products of **3** (50 mM) in DMSO (with **30** 58 mM) using Gr as cathode and Pt as anode. Results are reported in Table S1 and Figures S9-S10.

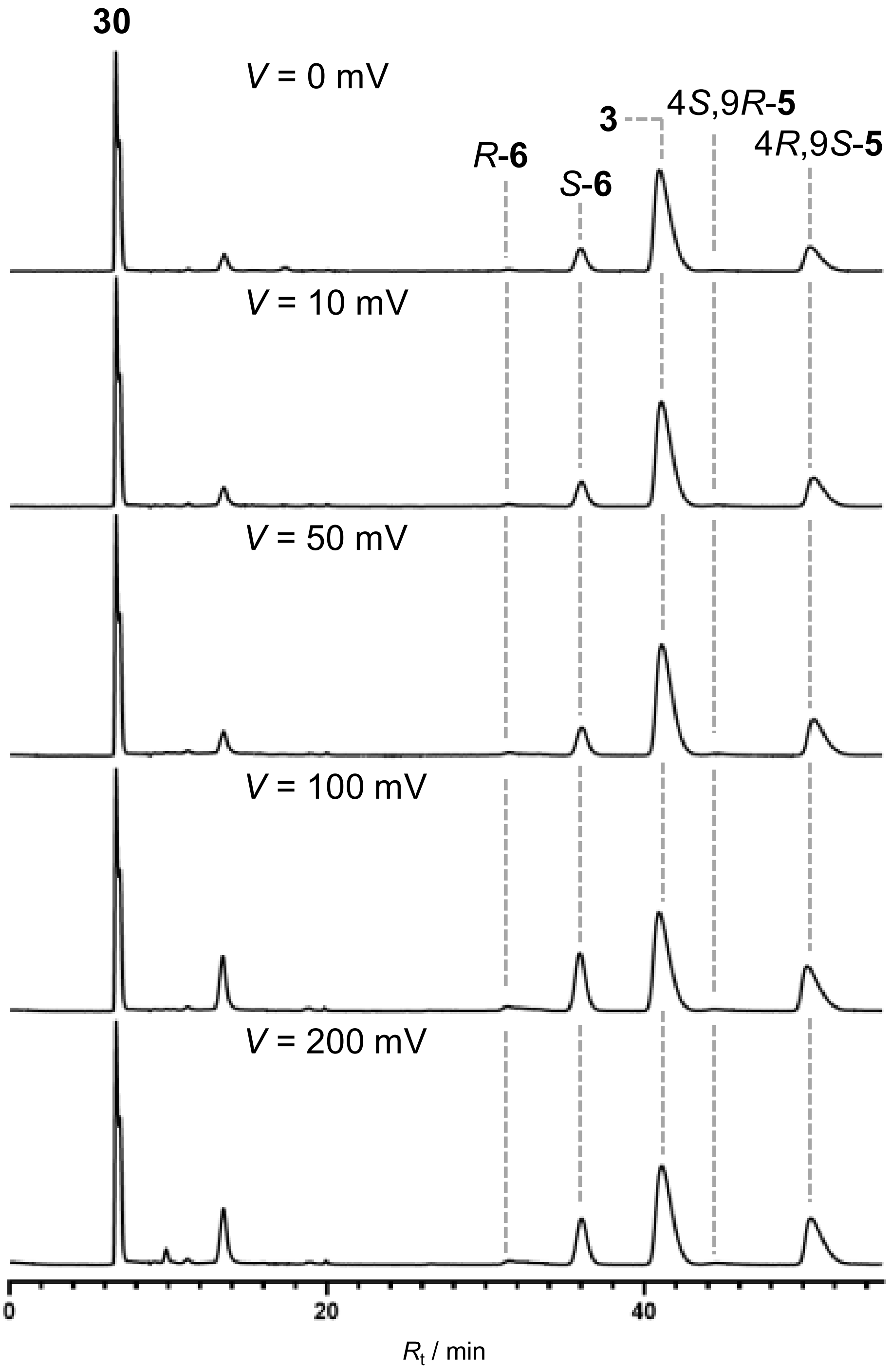

Figure S31. HPLC profiles showing the Robinson annulation products of **3** (50 mM) in DMSO (with **30** 50 mM) using Gr as cathode and Pt as anode. Results are reported in Table S1 and Figures S9-S10.

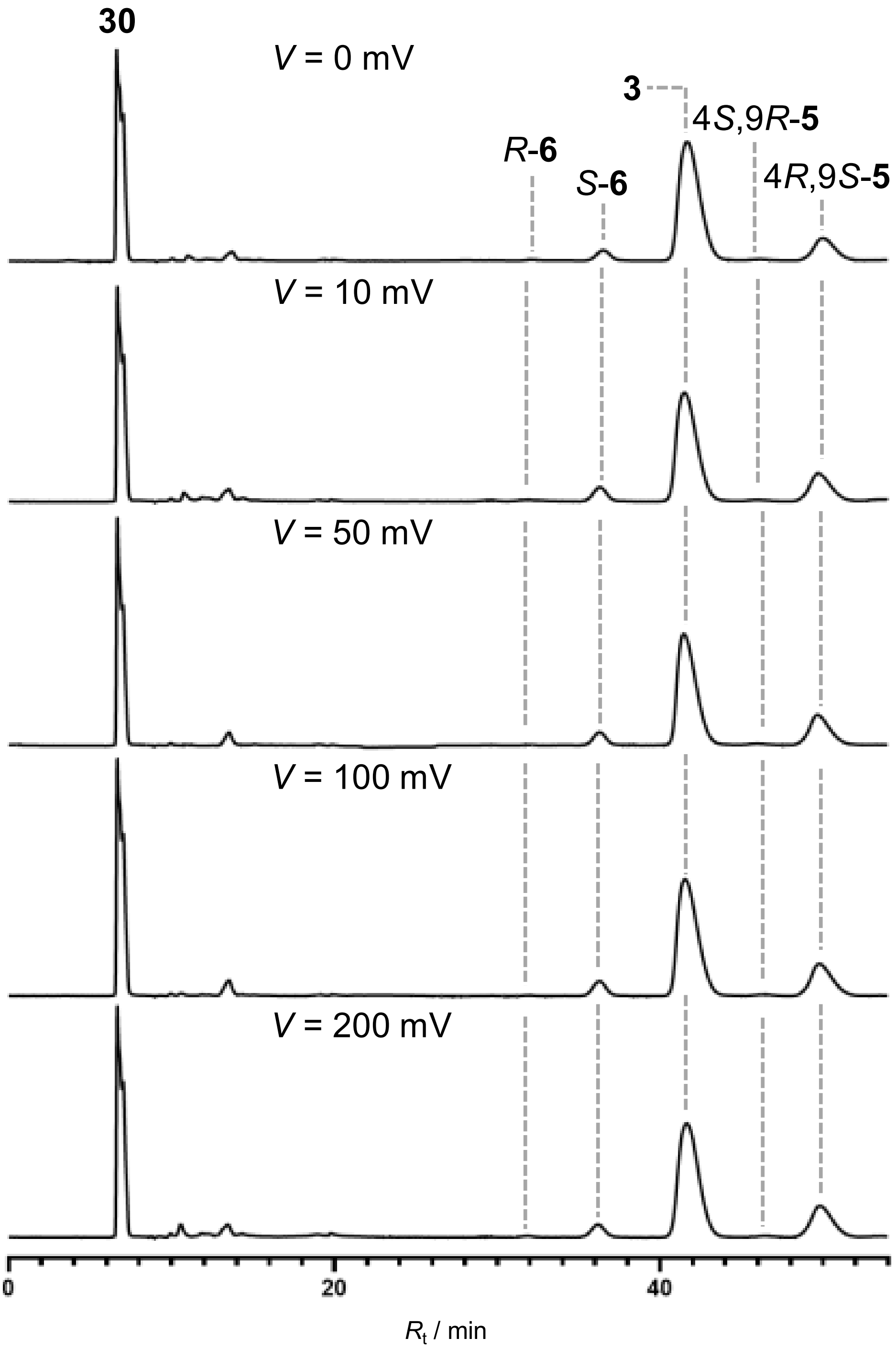

Figure S32. HPLC profiles showing the Robinson annulation products of **3** (50 mM) in DMSO (with **30** 52 mM) using Gr as anode and Pt as cathode. Results are reported in Table S1 and Figure S9.

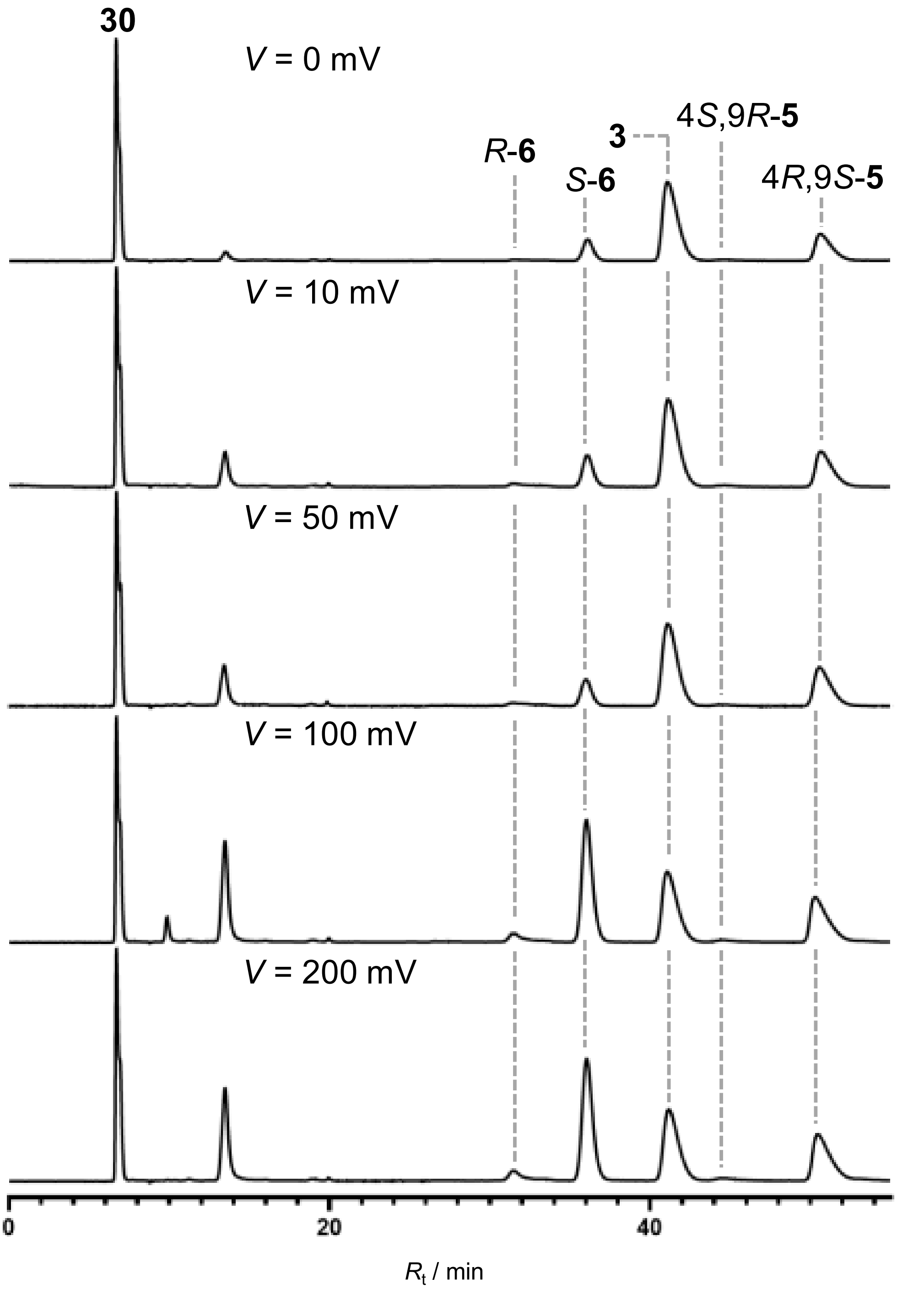

Figure S33. HPLC profiles showing the Robinson annulation products of **3** (50 mM) in DMSO (with **30** 55 mM, Na/**2** 1.00 mM) using Gr as cathode and Pt as anode. Results are reported in Table S2 and Figure S10.

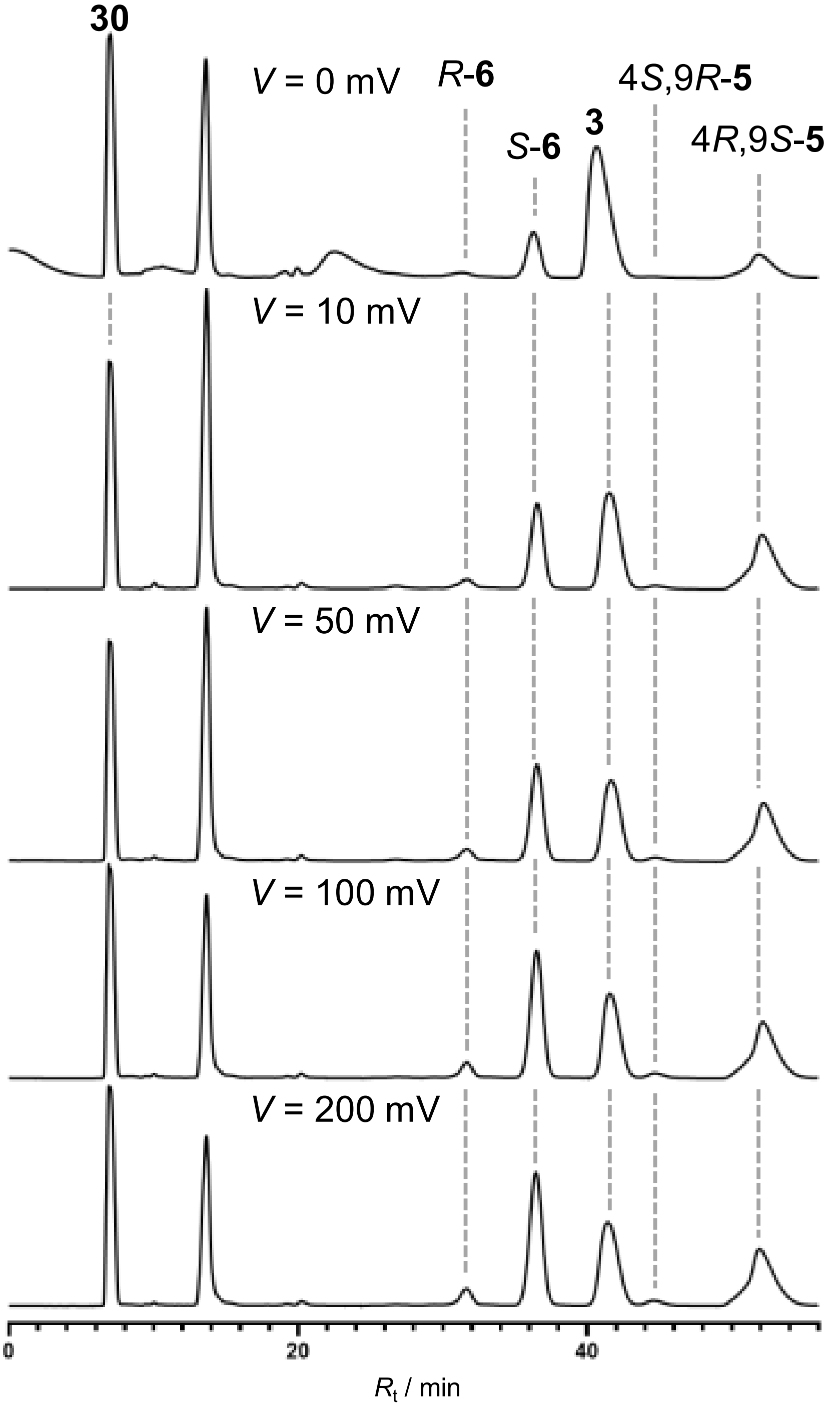

Figure S34. HPLC profiles showing the Robinson annulation products of **3** (50 mM) in DMSO (with **30** 58 mM, **1**/Cl 1.00 mM, calculated based on the number of charged residues) using Gr as cathode and Pt as anode. Results are reported in Table S2 and Figure S10. The baseline of the first chromatogram was unstable, which was due to residual impurities in the chromatography column.

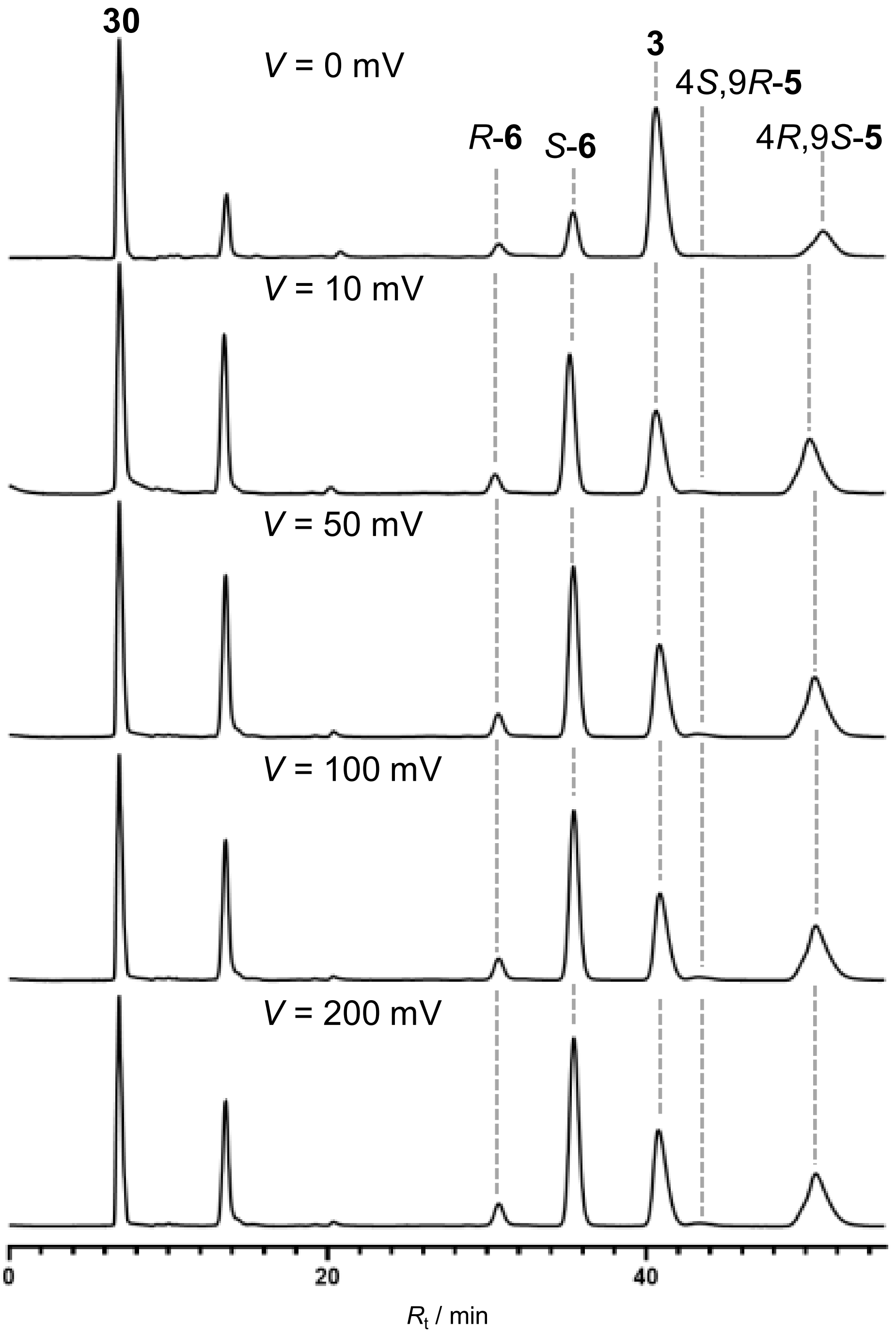

Figure S35. HPLC profiles showing the Robinson annulation products of **3** (50 mM) in DMSO (with **30** 50 mM, **1**/**2** 1.00 mM, calculated based on the number of charged residues) using Gr as cathode and Pt as anode. Results are reported in Table S2 and Figure S10.

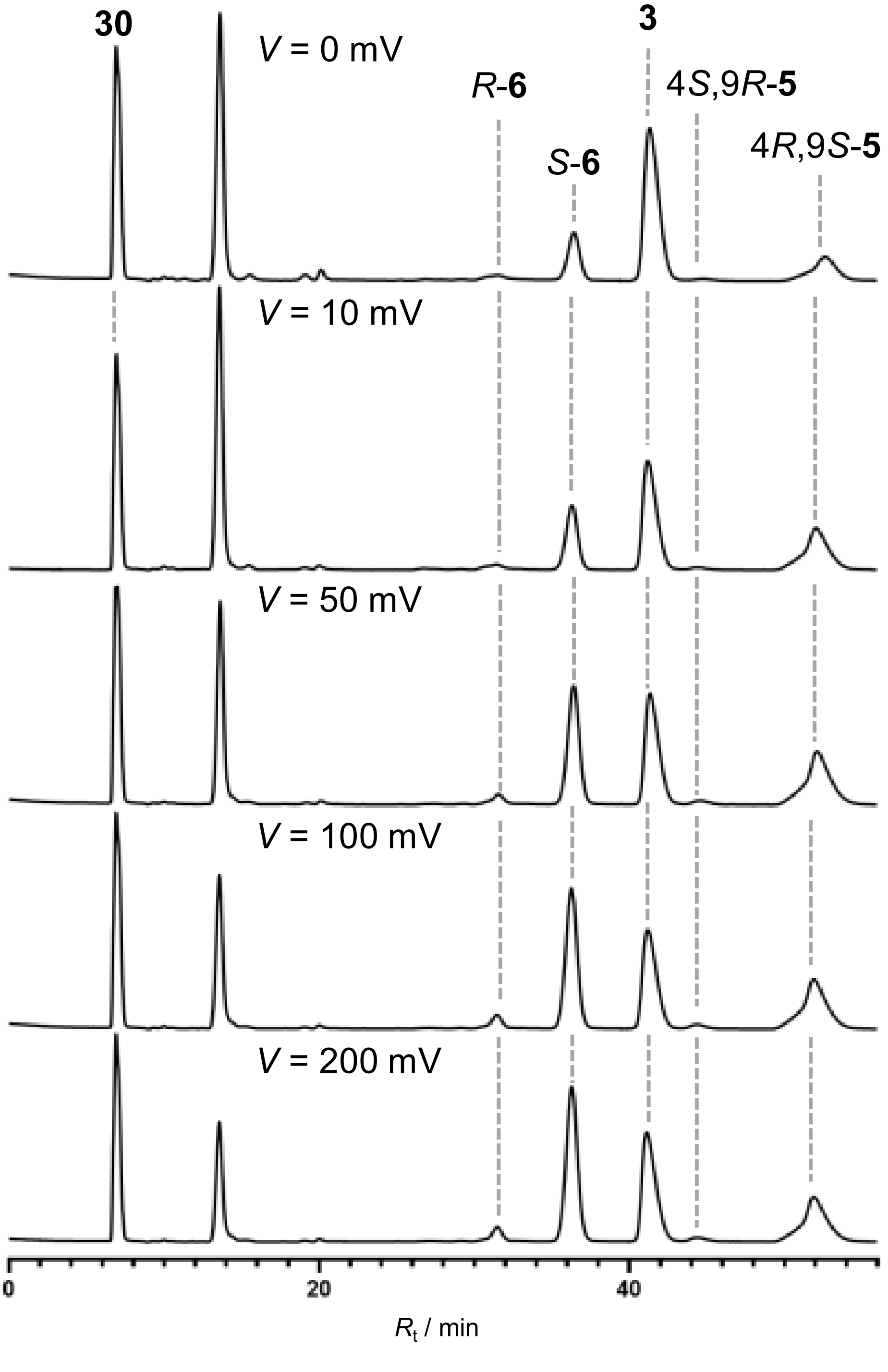

Figure S36. HPLC profiles showing the Robinson annulation products of **3** (50 mM) in DMSO (with **30** 56 mM, **1**/**14** 1.00 mM, calculated based on the number of charged residues) using Gr as cathode and Pt as anode. Results are reported in Table S3 and Figure S11.

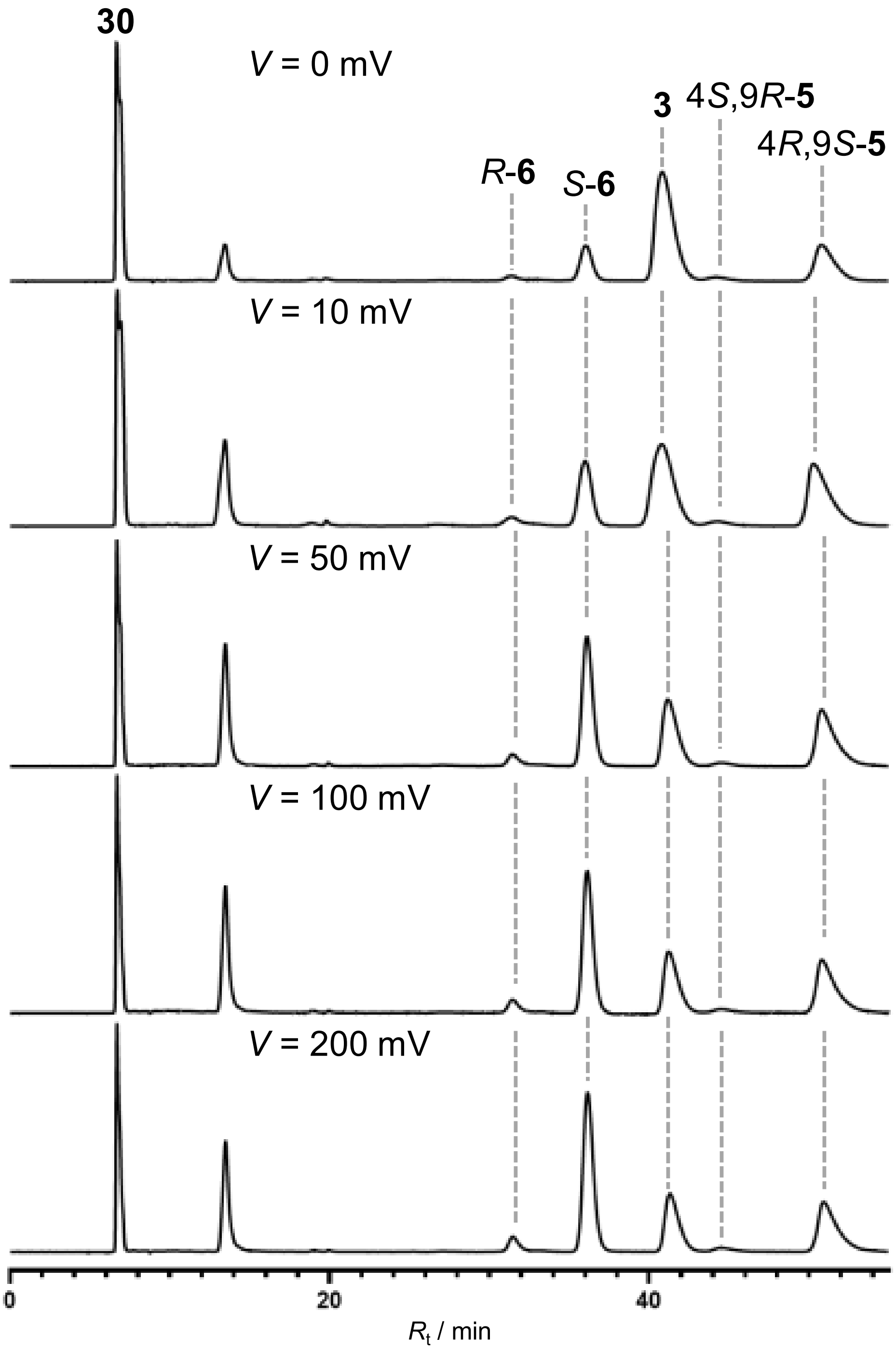

Figure S37. HPLC profiles showing the Robinson annulation products of **3** (50 mM) in DMSO (with **30** 52 mM, **1**/*M*-**15** 1.00 mM, calculated based on the number of charged residues) using Gr as cathode and Pt as anode. Results are reported in Table S3 and Figure S11.

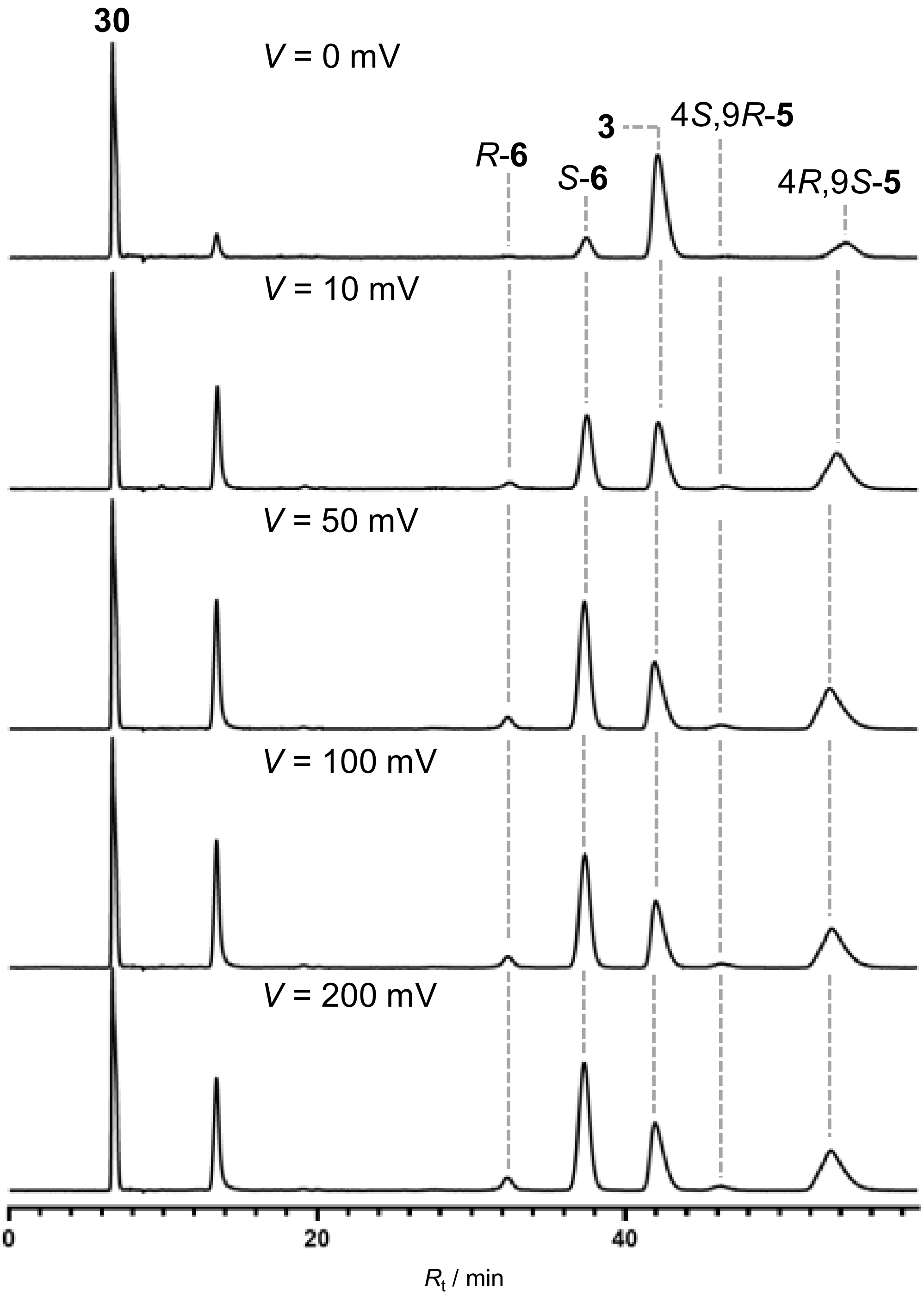

Figure S38. HPLC profiles showing the Robinson annulation products of **3** (50 mM) in DMSO (with **30** 52 mM, **1**/*P*-**15** 1.00 mM, calculated based on the number of charged residues) using Gr as cathode and Pt as anode. Results are reported in Table S3 and Figure S11.

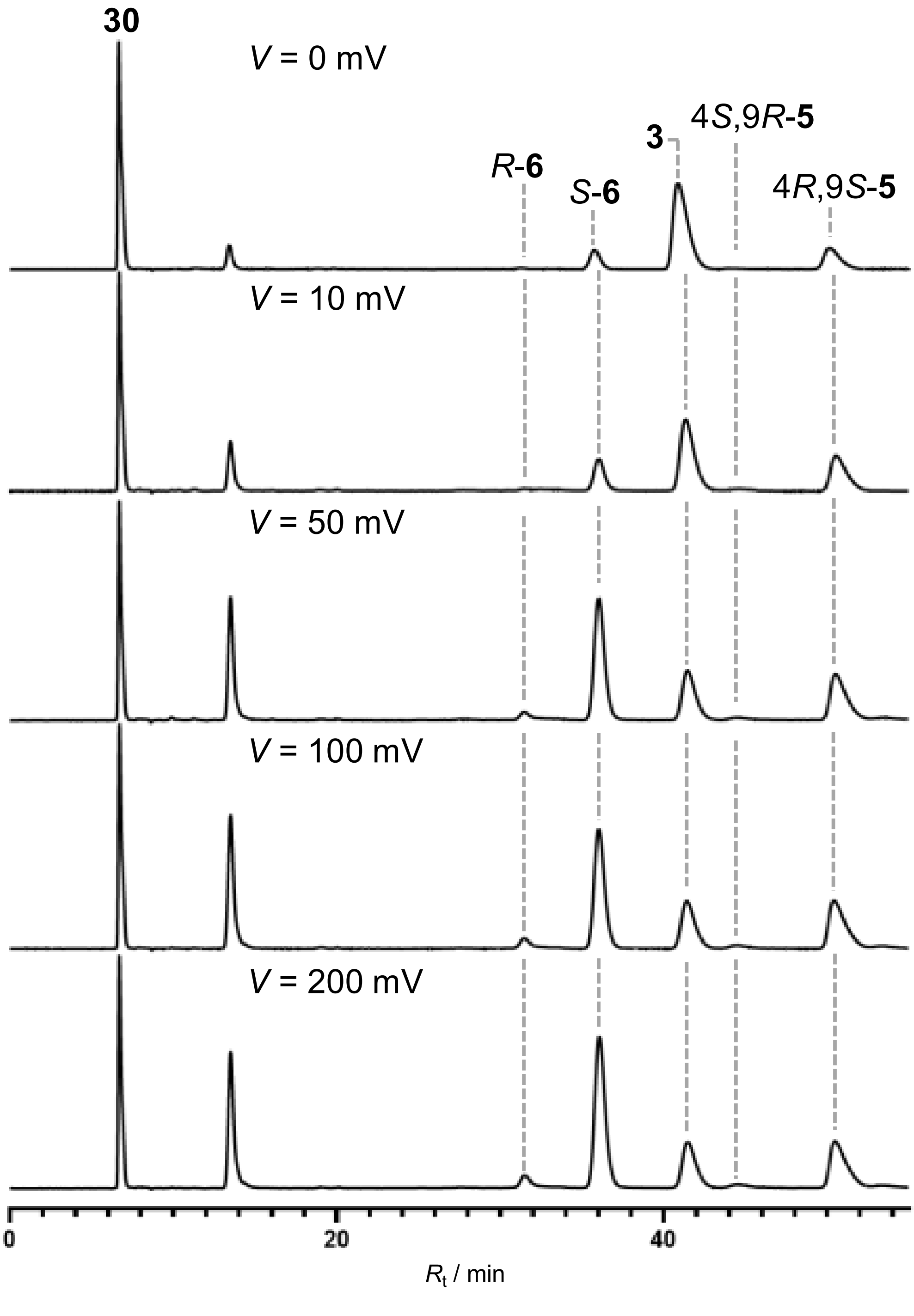

Figure S39. HPLC profiles showing the Robinson annulation products of **3** (50 mM) in DMSO (with **30** 55 mM, **1**/**17** 1.00 mM, calculated based on the number of charged residues) using Gr as cathode and Pt as anode. Results are reported in Table S3 and Figure S11.

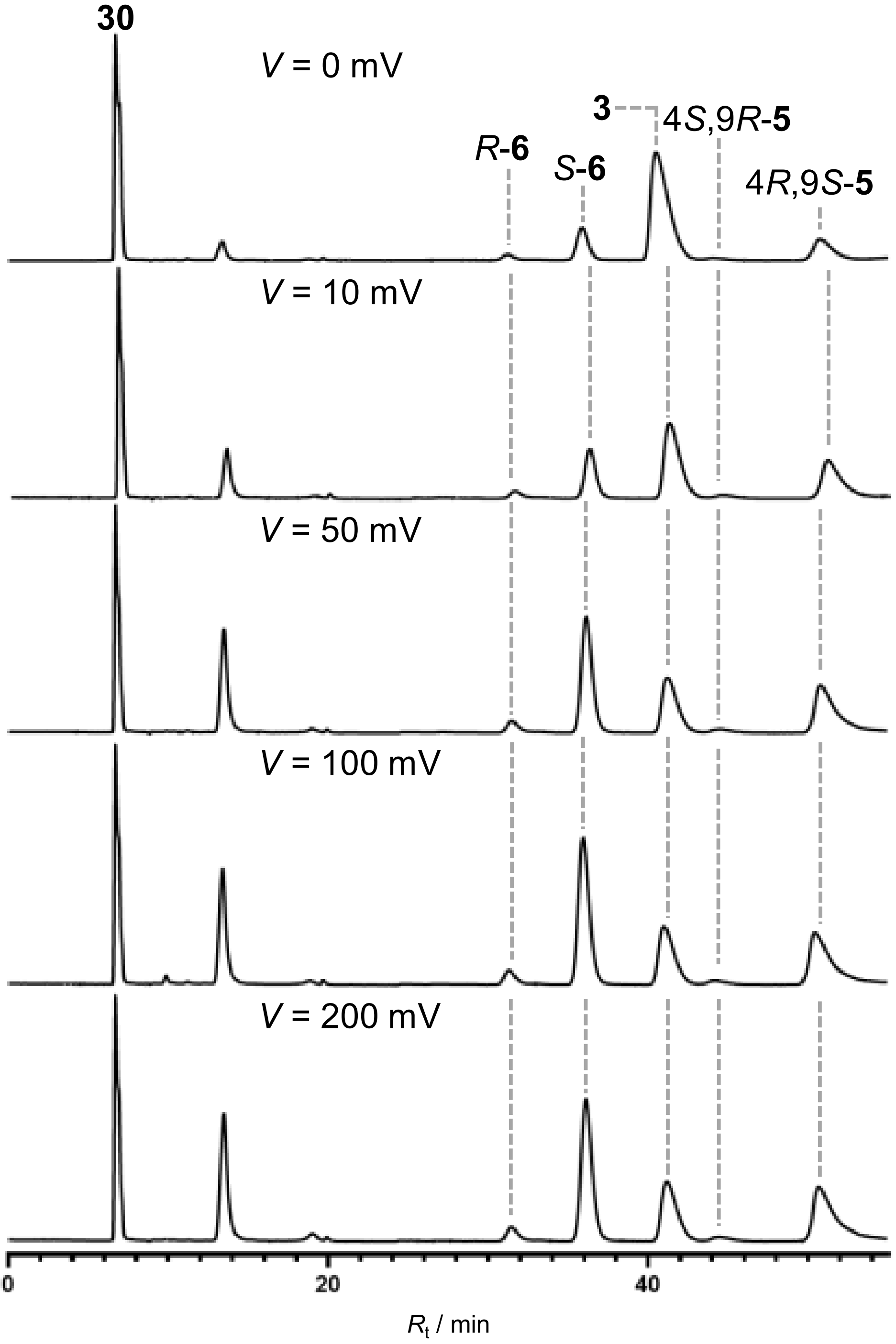

Figure S40. HPLC profiles showing the Robinson annulation products of **3** (50 mM) in DMSO (with **30** 54 mM, **1**/**16** 1.00 mM, calculated based on the number of charged residues) using Gr as cathode and Pt as anode. Results are reported in Table S3 and Figure S11.

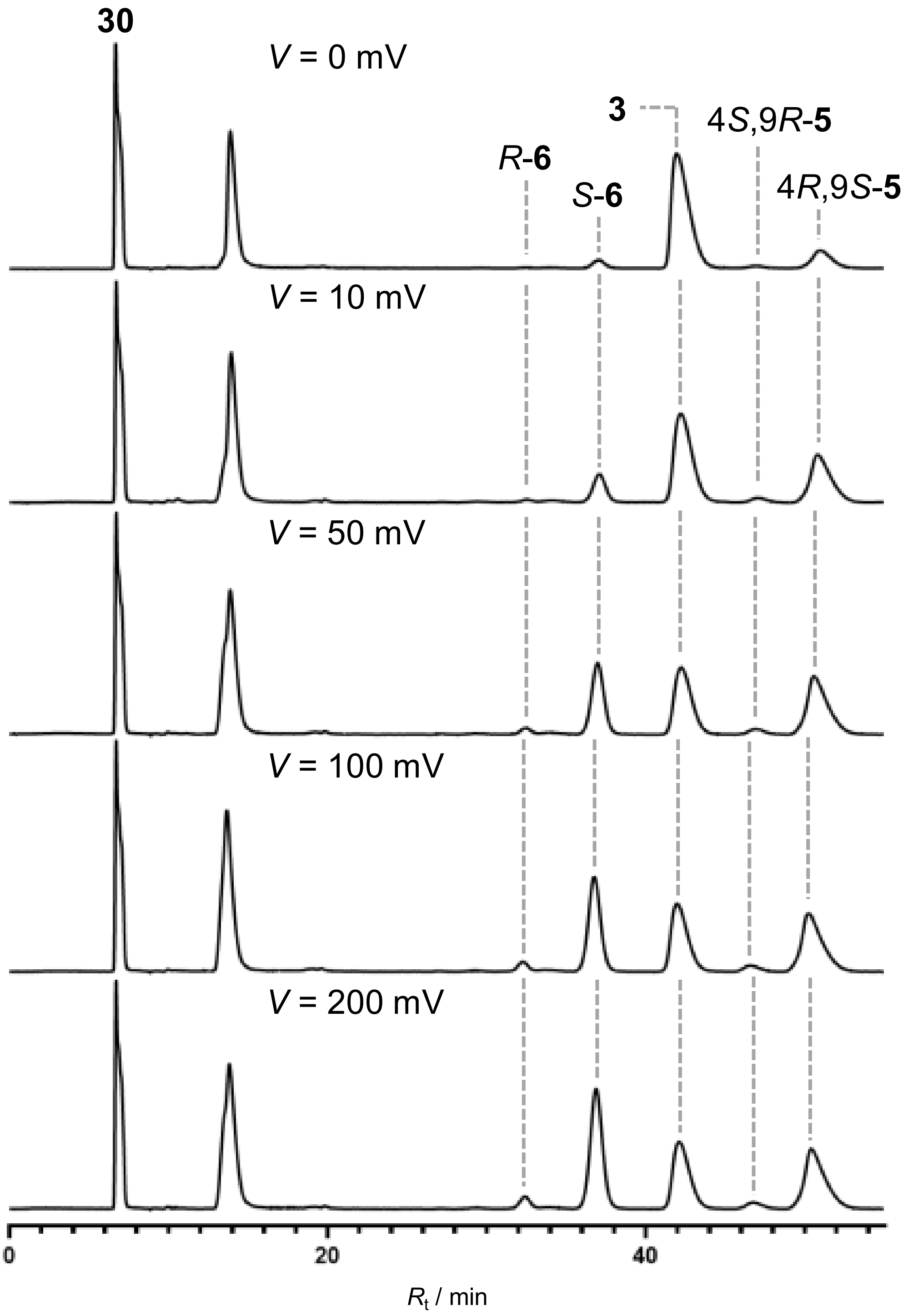

Figure S41. HPLC profiles showing the Robinson annulation products of **3** (50 mM) in DMSO (with **30** 51 mM, **1**/**16** 1.00 mM, calculated based on the number of charged residues) using Gr as cathode and Pt as anode. Results are reported in Table S3 and Figure S11.

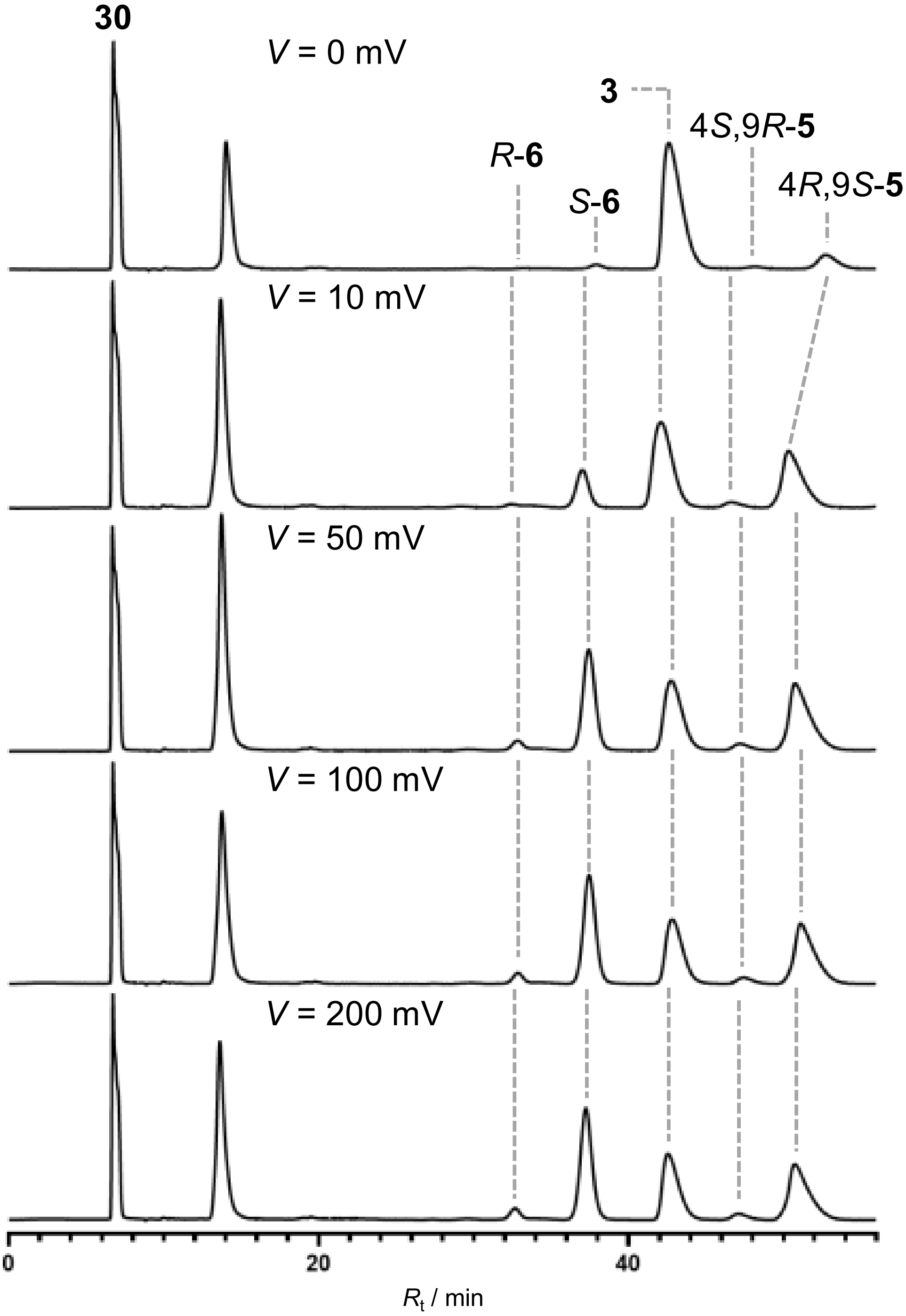

Figure S42. HPLC profiles showing the Robinson annulation products of **3** (50 mM) in DMSO (with **30** 52 mM, **1**/**16** 1.00 mM, calculated based on the number of charged residues) using Gr as cathode and Pt as anode. Results are reported in Table S3 and Figure S11.

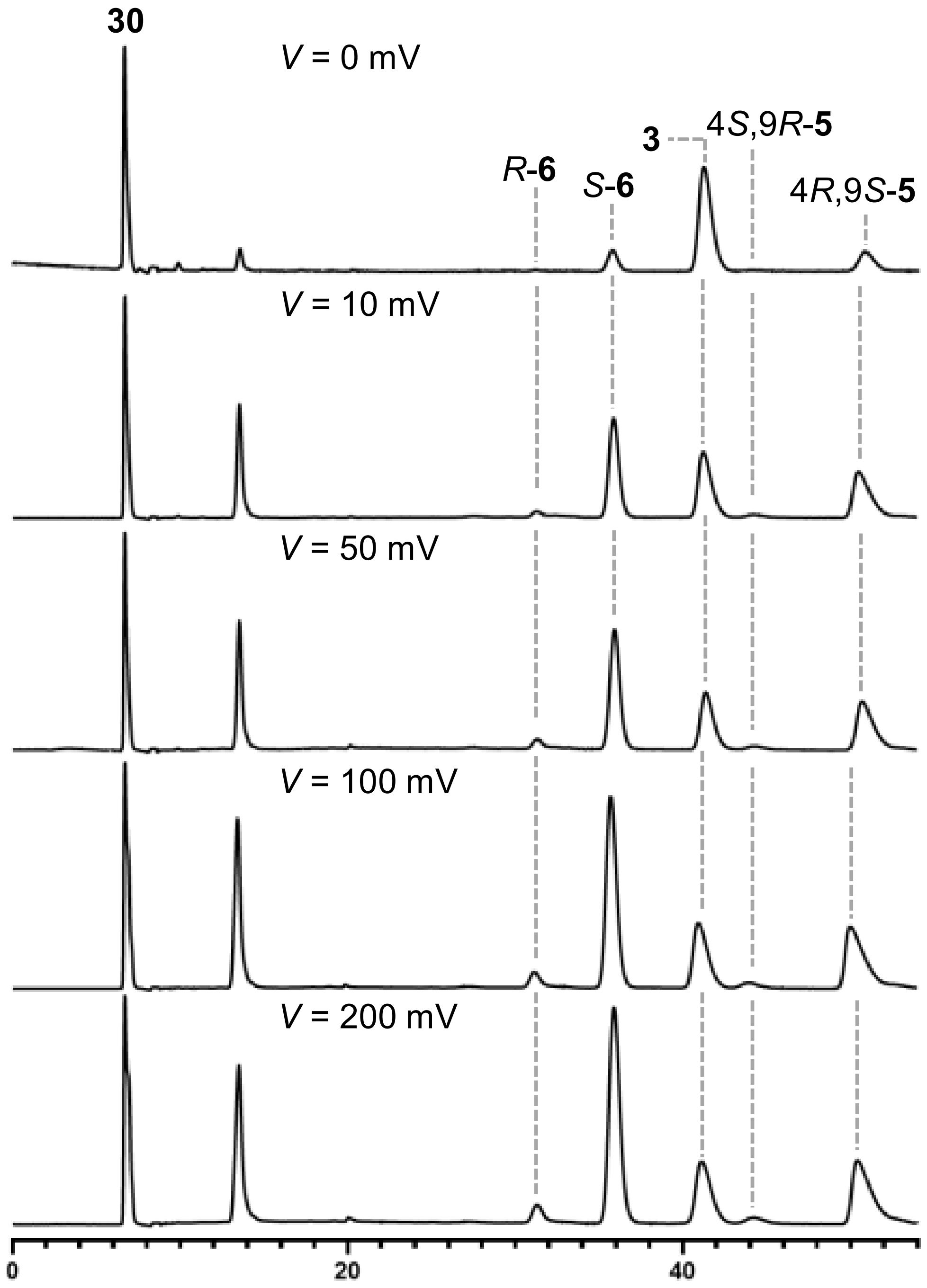

Figure S43. HPLC profiles showing the Robinson annulation products of **3** (50 mM) in DMSO (with **30** 50 mM, **10**/**16** 1.00 mM, calculated based on the number of charged residues) using Gr as cathode and Pt as anode. Results are reported in Table S4 and Figure S12.

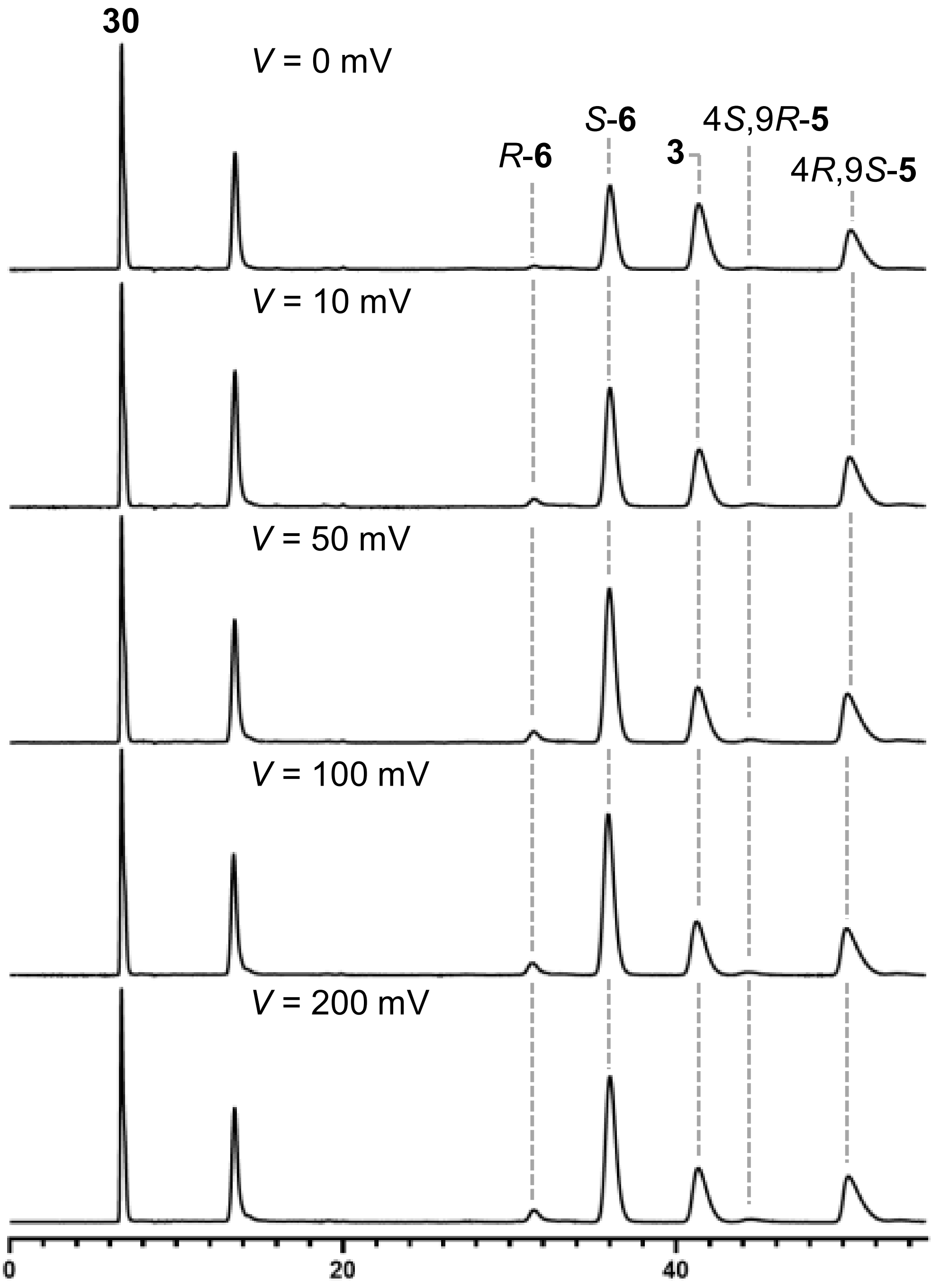

Figure S44. HPLC profiles showing the Robinson annulation products of **3** (50 mM) in DMSO (with **30** 57 mM, **13**/**2** 1.00 mM, calculated based on the number of charged residues) using Gr as cathode and Pt as anode. Results are reported in Table S4 and Figure S12.

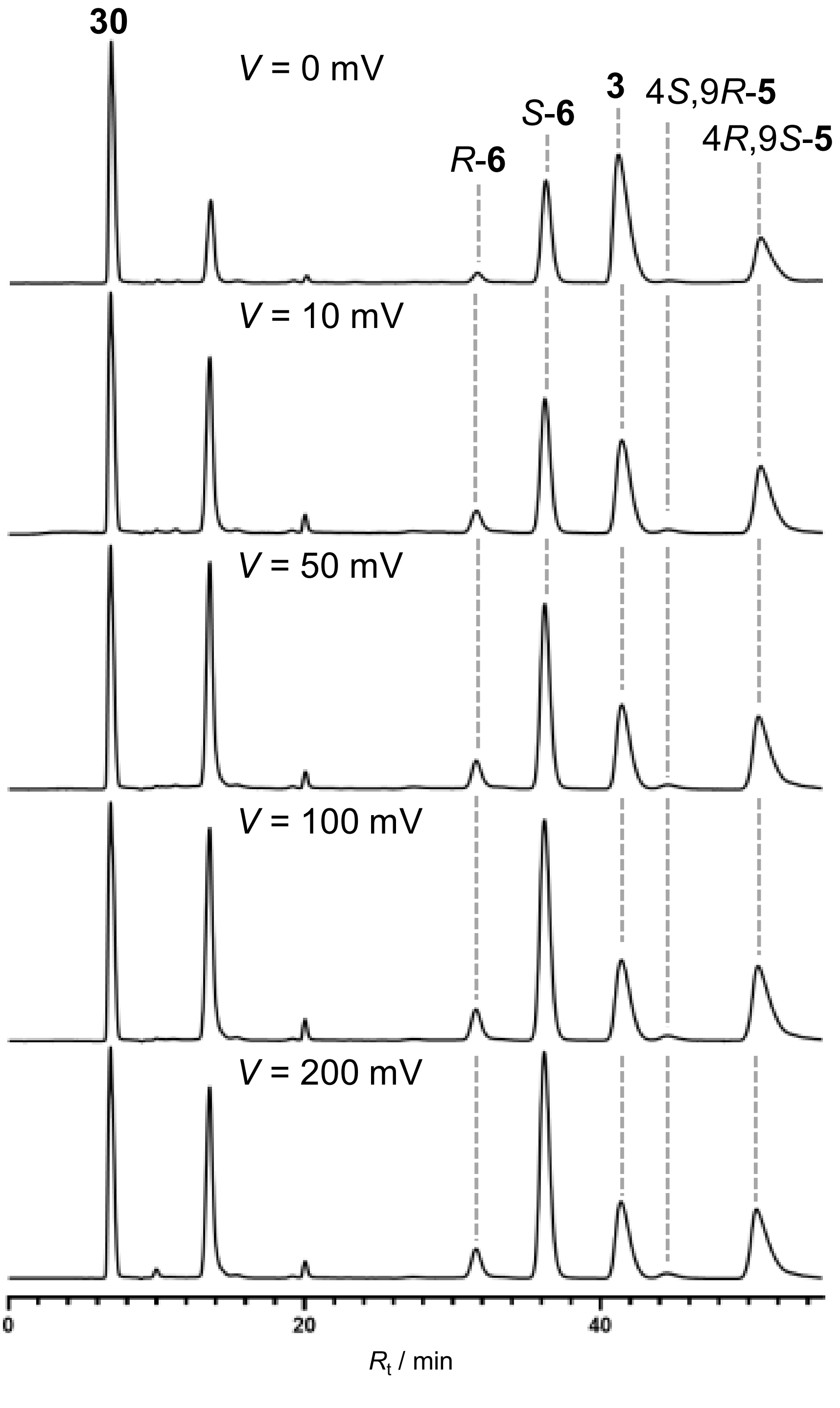

Figure S45. HPLC profiles showing the Robinson annulation products of **3** (50 mM) in DMSO (with **30** 55 mM, **11**/**2** 1.00 mM, calculated based on the number of charged residues) using Gr as cathode and Pt as anode. Results are reported in Table S4 and Figure S12.

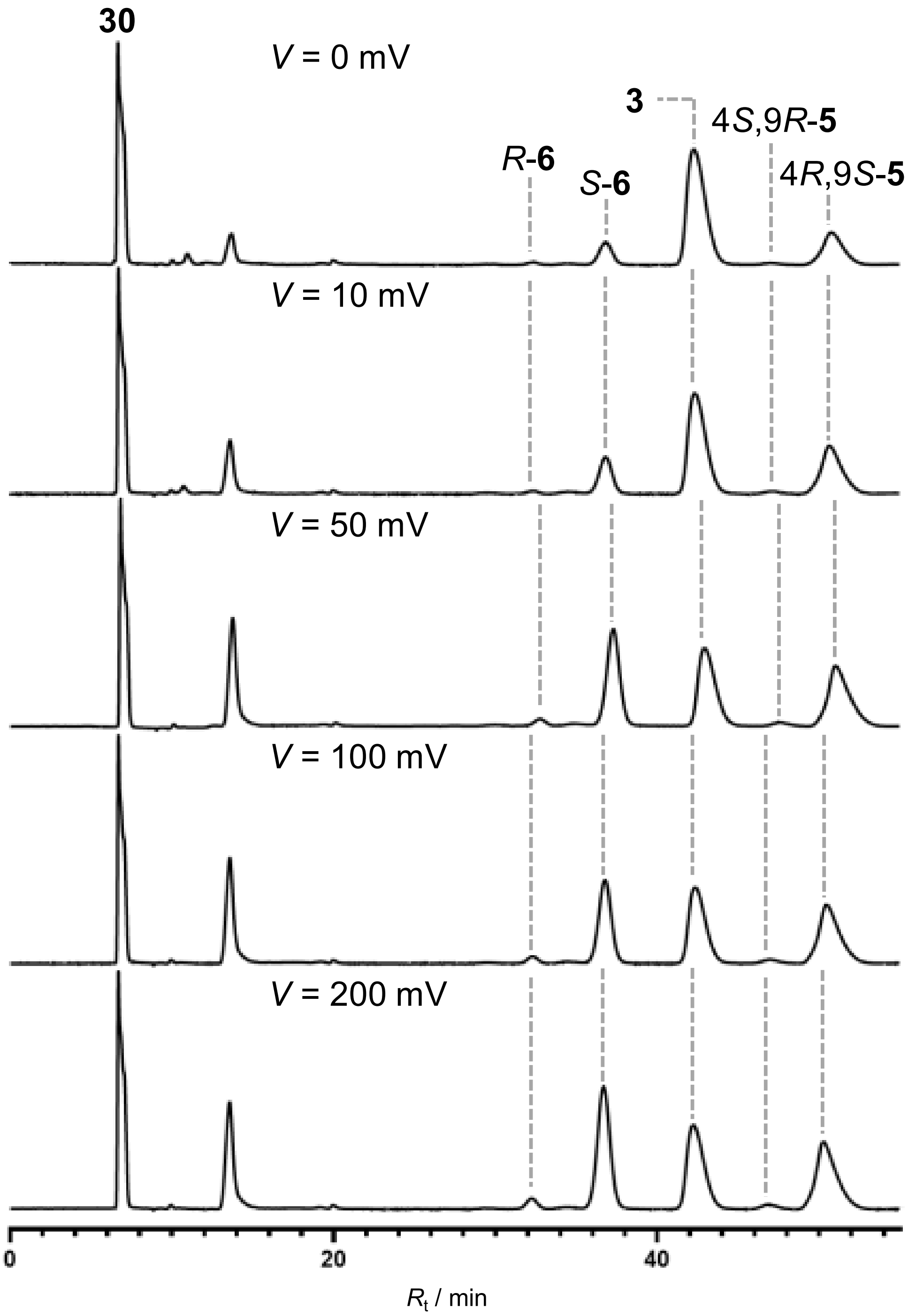

Figure S46. HPLC profiles showing the Robinson annulation products of **3** (50 mM) in DMSO (with **30** 50 mM, **12**/Cl 1.00 mM) using Gr as cathode and Pt as anode. Results are reported in Table S4 and Figure S12.

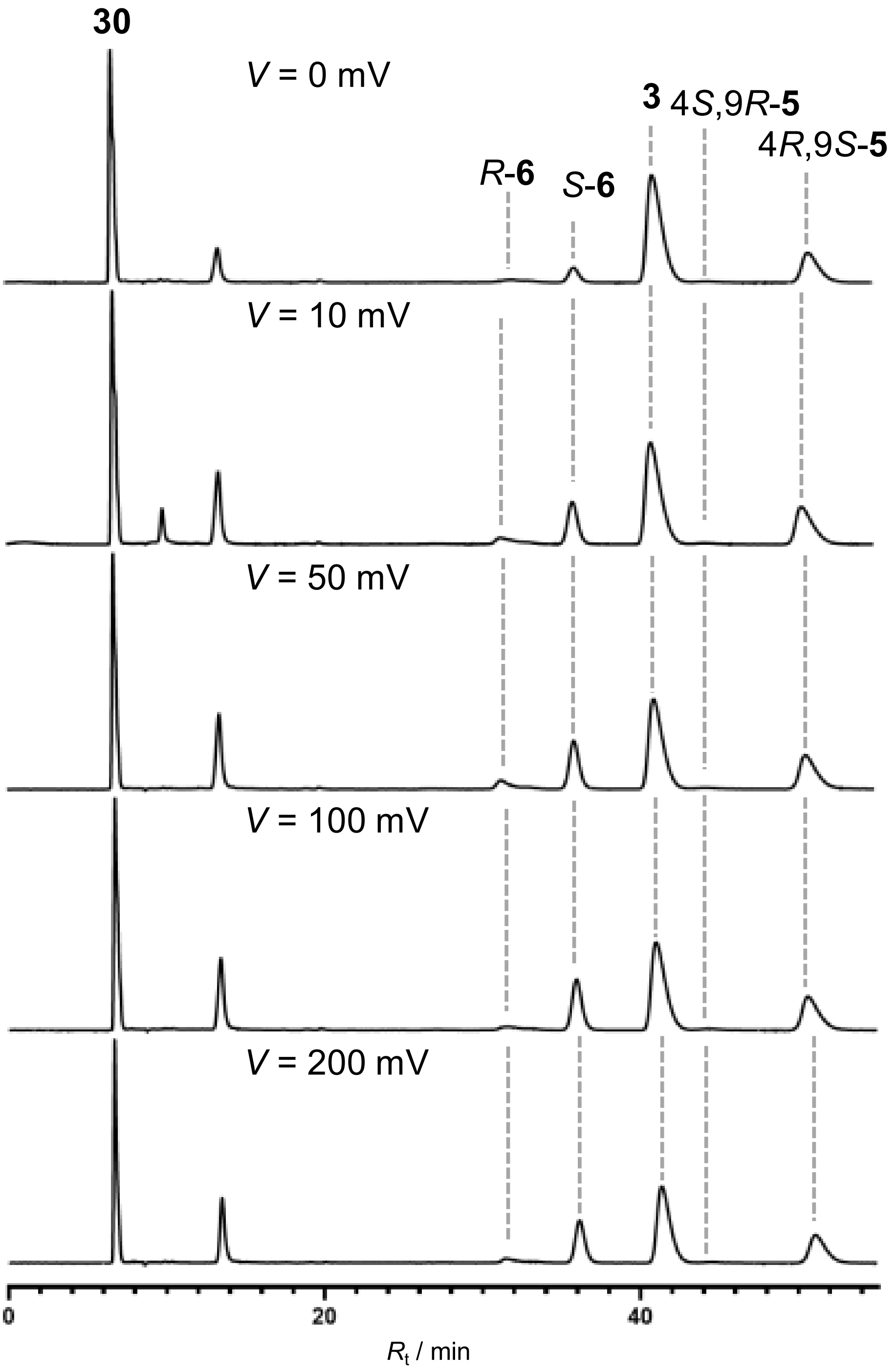

Figure S47. HPLC profiles showing the Robinson annulation products of **3** (50 mM) in DMSO (with **30** 50 mM, **7**/Cl 1.00 mM) using Gr as cathode and Pt as anode. Results are reported in Table S5 and Figure S13.

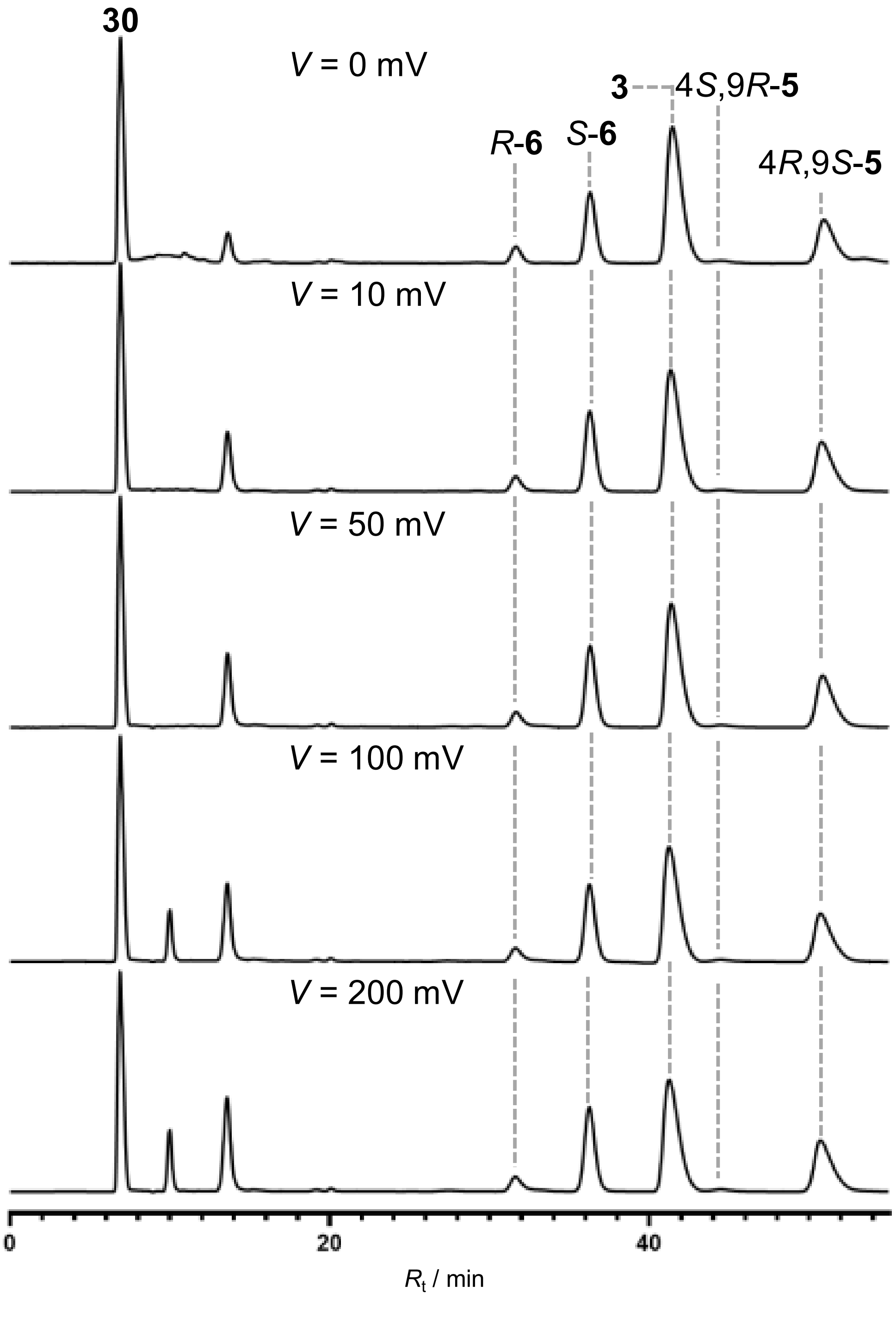

Figure S48. HPLC profiles showing the Robinson annulation products of **3** (50 mM) in DMSO (with **30** 55 mM, **8**/**2** 1.00 mM) using Gr as cathode and Pt as anode. Results are reported in Table S5 and Figure S13.

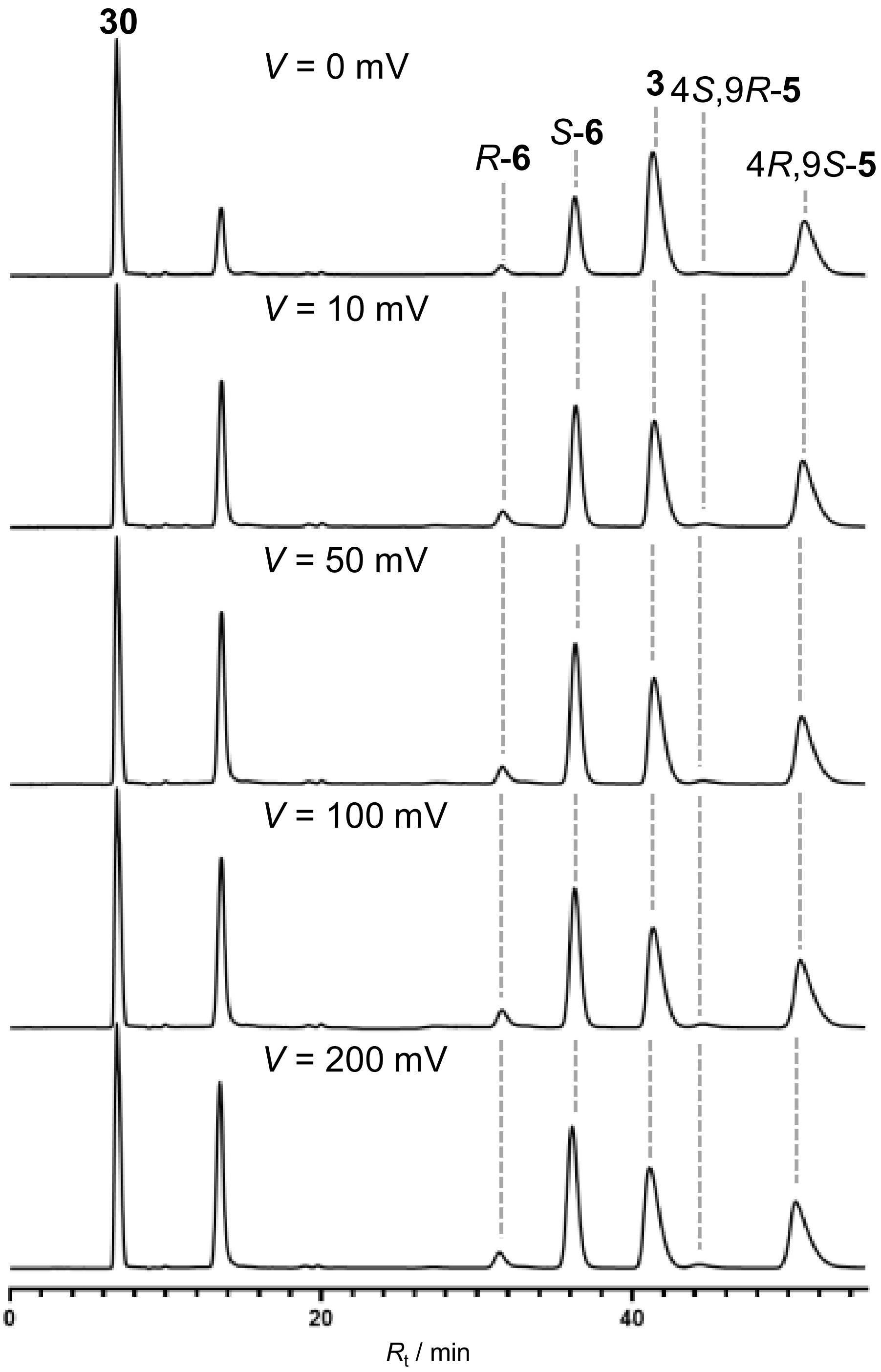

Figure S49. HPLC profiles showing the Robinson annulation products of **3** (50 mM) in DMSO (with **30** 52 mM, **9**/**2** 1.00 mM) using Gr as cathode and Pt as anode. Results are reported in Table S5 and Figure S13.

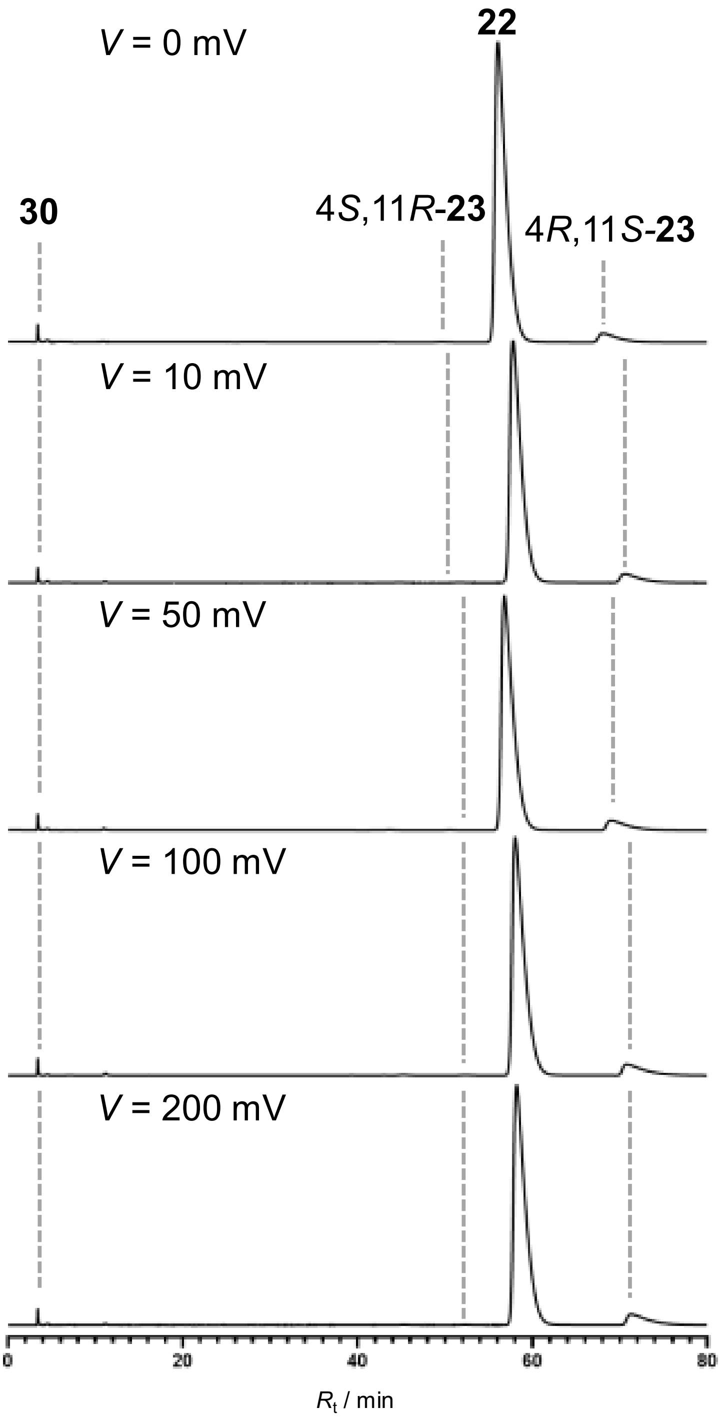

Figure S50. HPLC profiles showing the Robinson annulation products of **22** (50 mM) in DMSO (with **30** 42 mM) using Gr as cathode and Pt as anode. Results are reported in Table S6 and Figure S14.

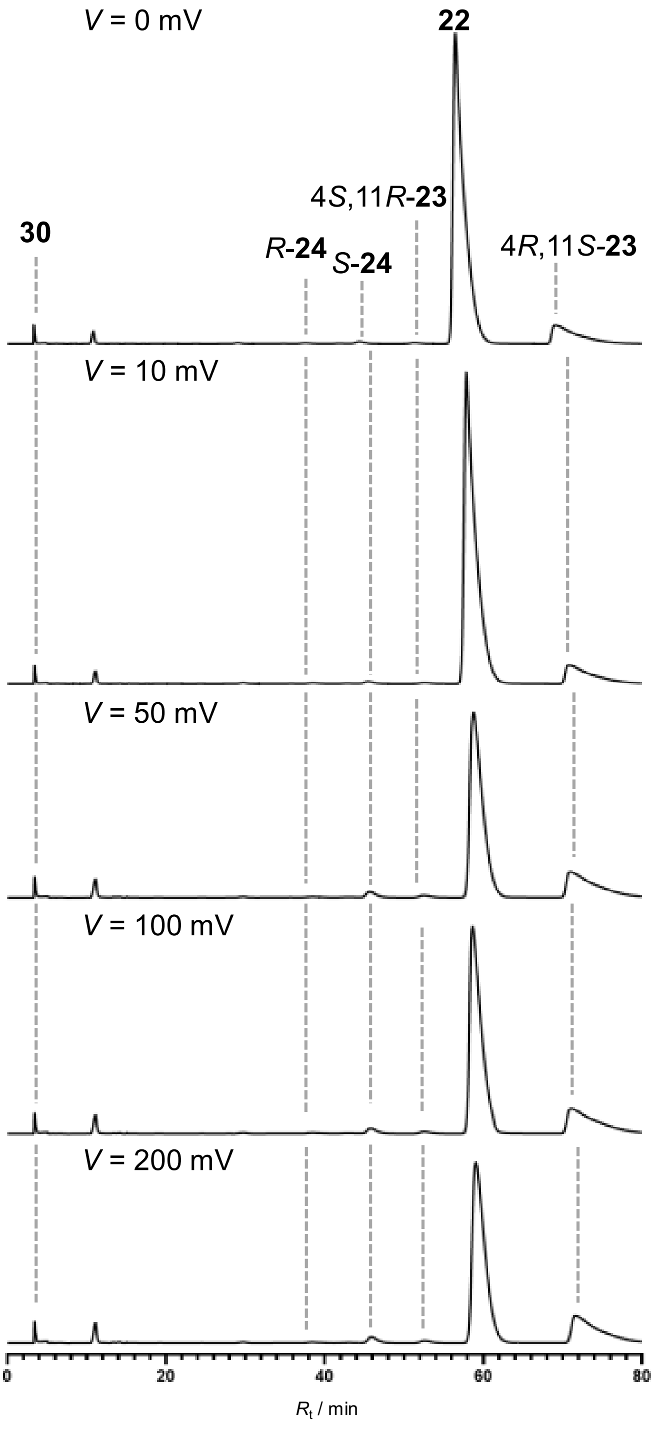

Figure S51. HPLC profiles showing the Robinson annulation products of **22** (50 mM) in DMSO (with **30** 44 mM, **1**/**2** 1.00 mM, calculated based on the number of charged residues) using Gr as cathode and Pt as anode. Results are reported in Table S6 and Figure S14.

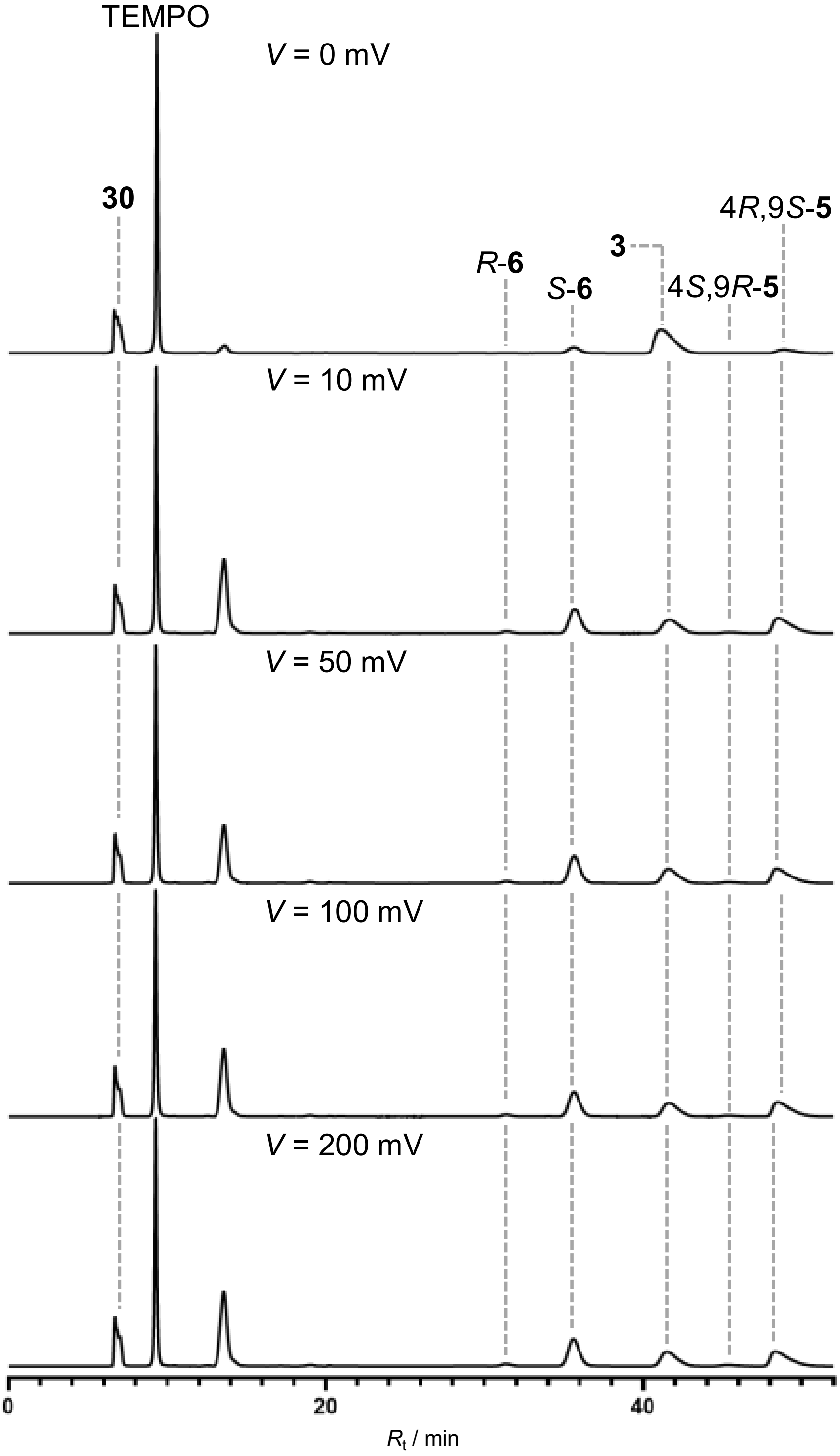

Figure S52. HPLC profiles showing the Robinson annulation products of **3** (50 mM) in DMSO (with **30** 49 mM, TEMPO 50 mM, **1**/**2** 1.00 mM, calculated based on the number of charged residues) using Gr as cathode and Pt as anode. Results are reported in Table S7 and Figure S15.

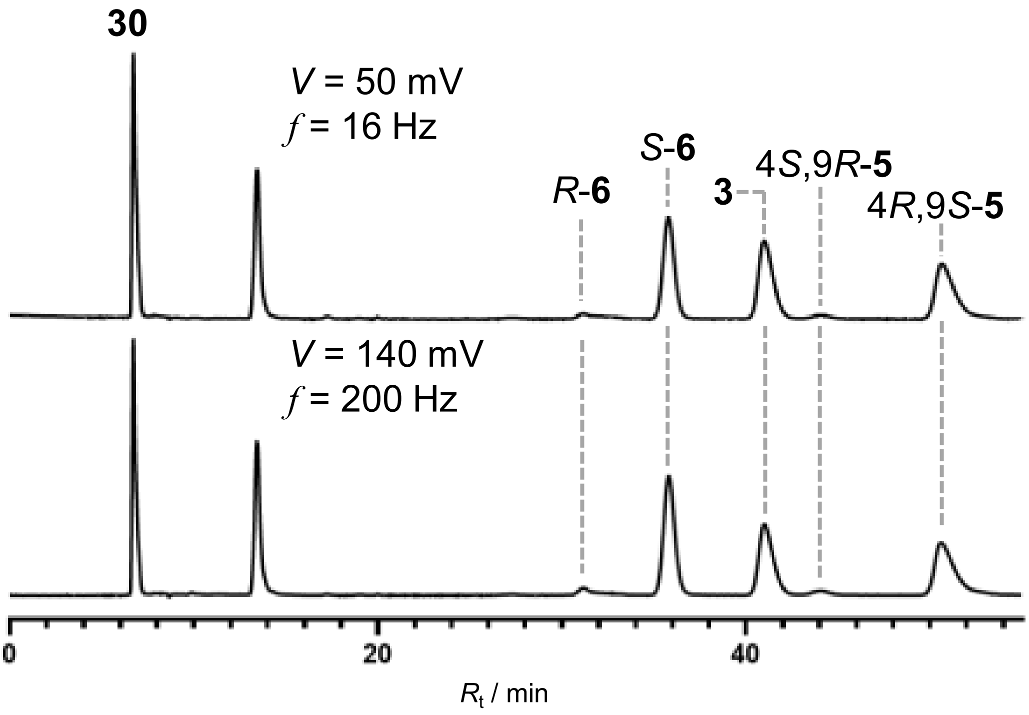

Figure S53. HPLC profiles showing the Robinson annulation products of **3** (50 mM) in DMSO (with **30** 50 mM, **1**/**2** 1.00 mM, calculated based on the number of charged residues) using two Gr as electrodes. Results are reported in Table S8 and Figure S16.

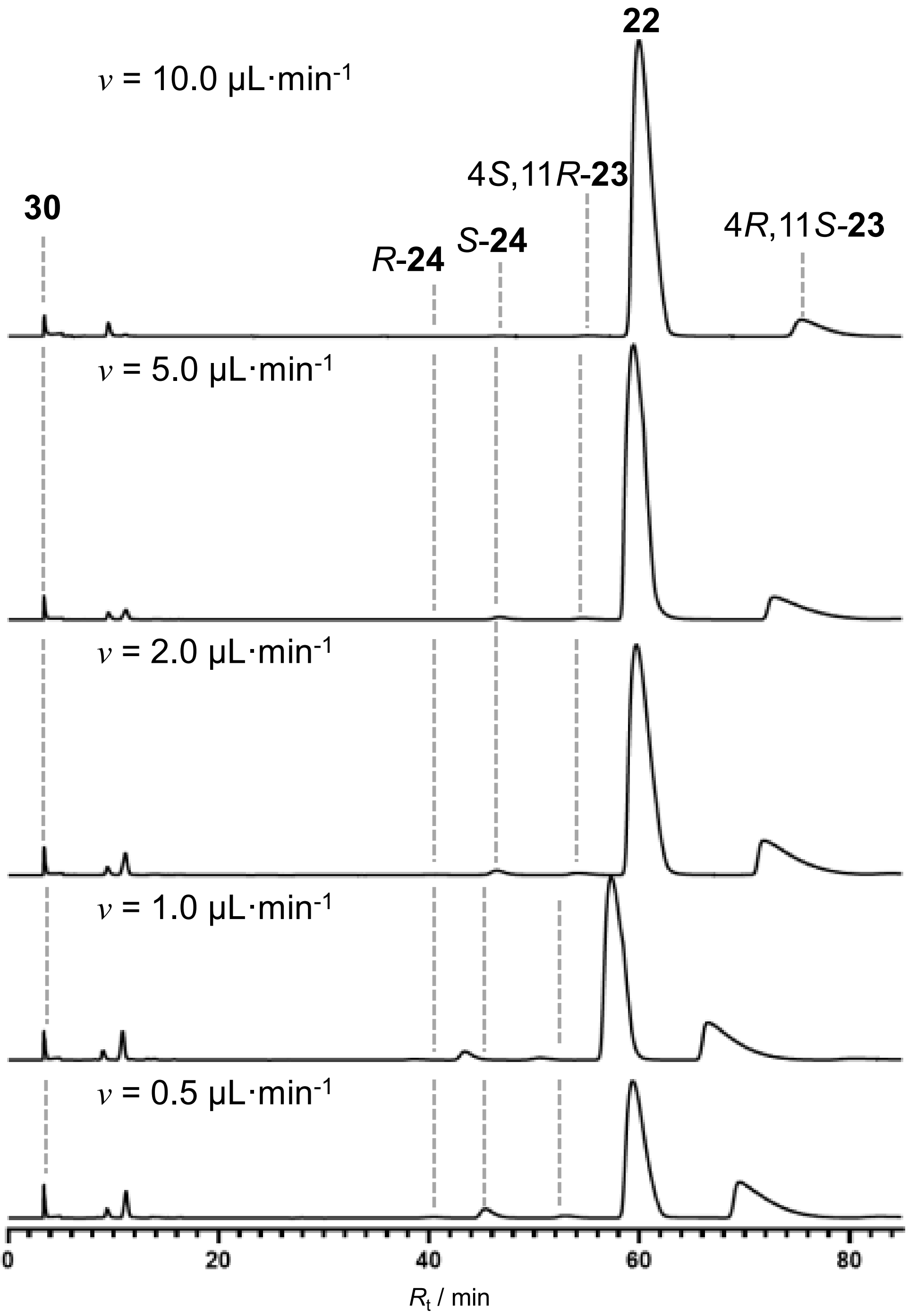

Figure S54. HPLC profiles showing the Robinson annulation products of **22** (50 mM) in DMSO (with **30** 50 mM, **1/16** 1.00 mM, calculated based on the number of charged residues) using Gr as cathode and Pt as anode. Results are reported in Table S9 and Figure S17.

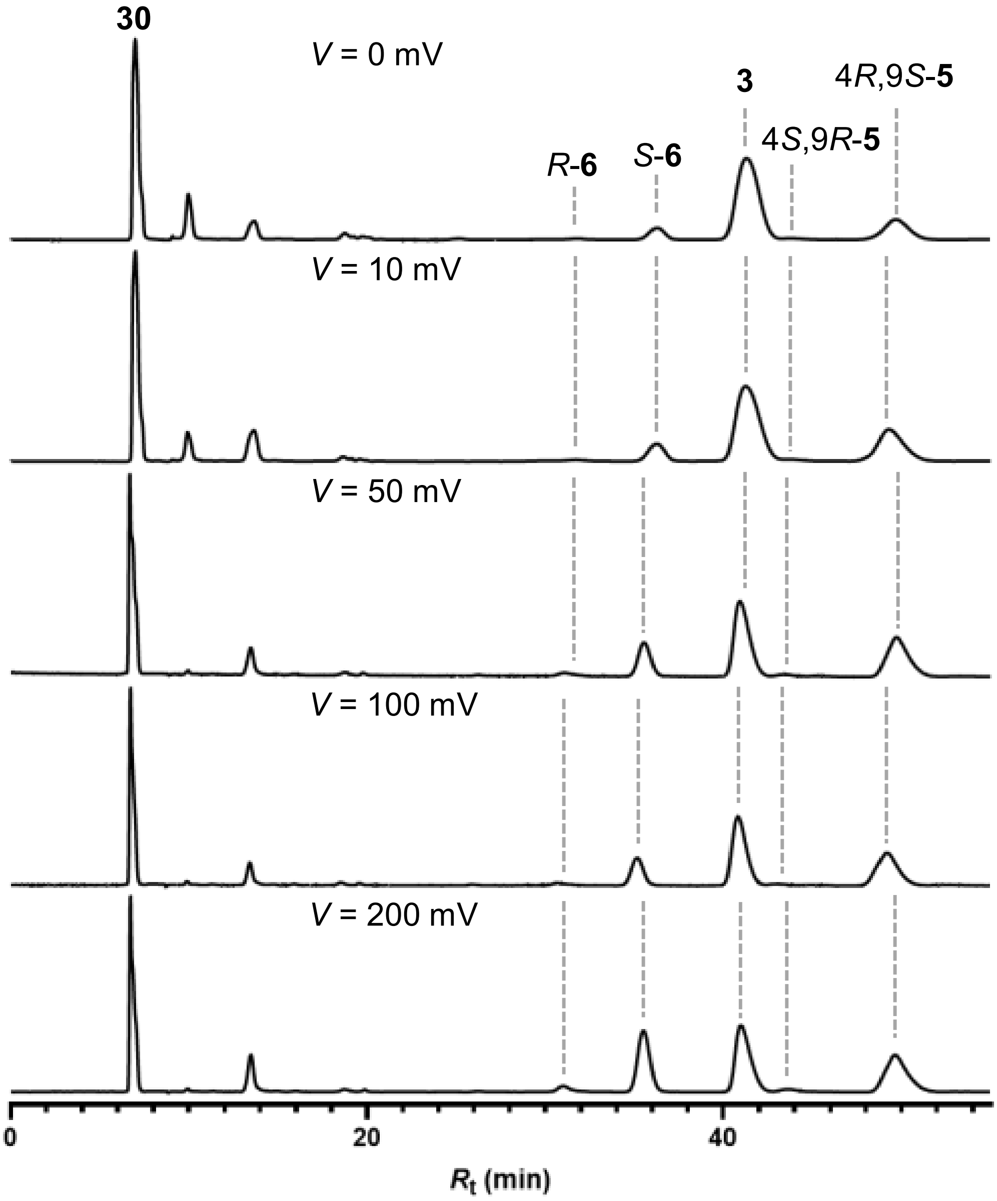

Figure S55. HPLC profiles showing the Robinson annulation products of **3** (50 mM) in DMSO (with **30** 54 mM, **1**/**2** 1.00 mM, calculated based on the number of charged residues) using Gr as cathode and Gr as anode. Results are reported in Table S10 and Figure S18.

7. NMR Spectra

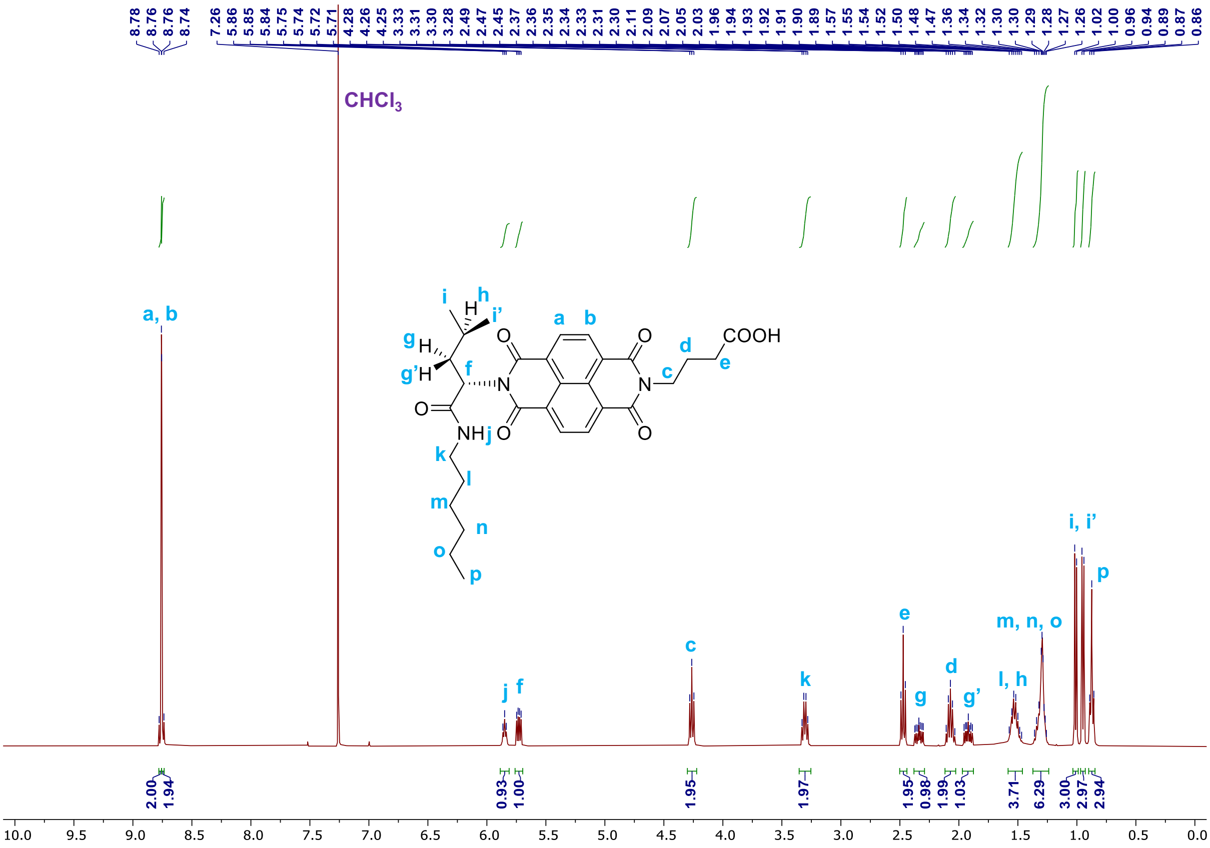

Figure S56. 400 MHz ^{1}H NMR spectrum of **29** in $CDCl_3$.

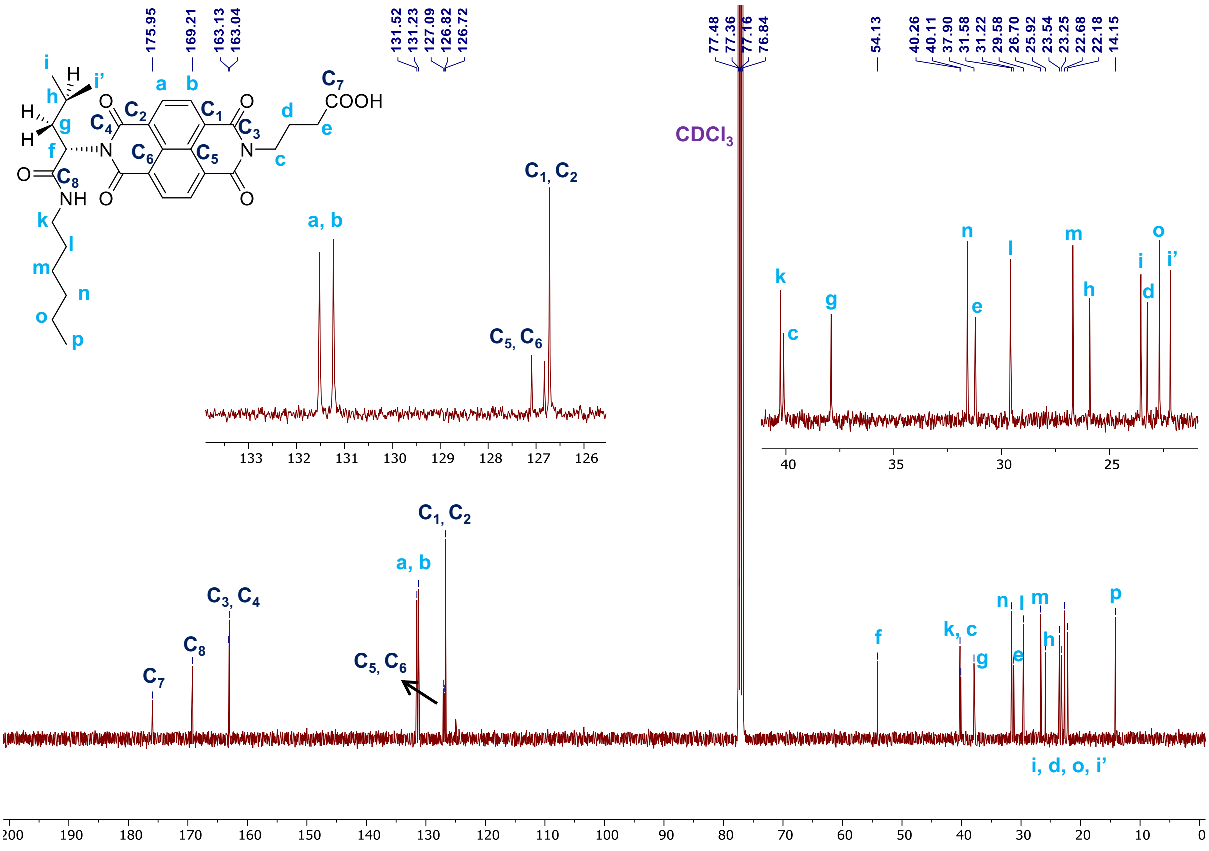

Figure S57. 101 MHz ^{13}C NMR spectrum of **29** in $CDCl_3$.

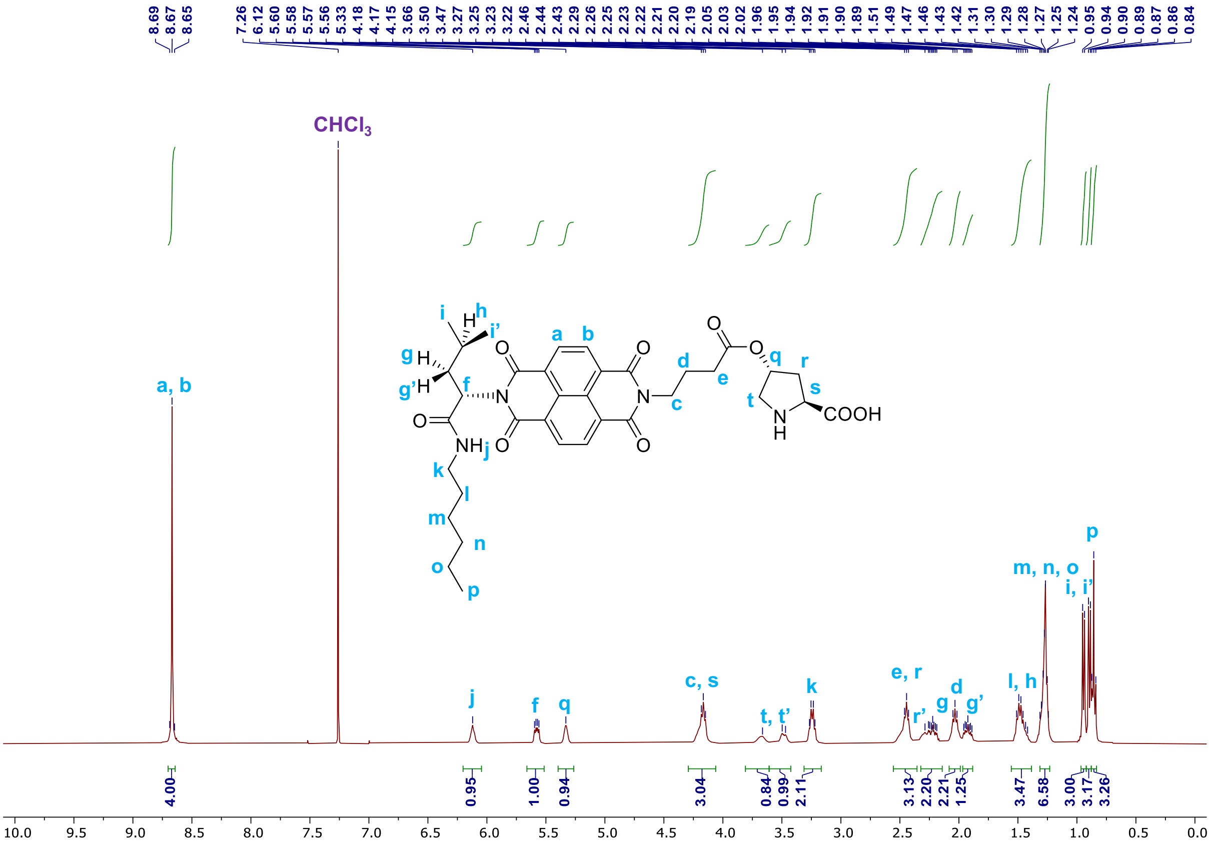

Figure S58. 400 MHz ^{1}H NMR spectrum of **4** in $CDCl_3$.

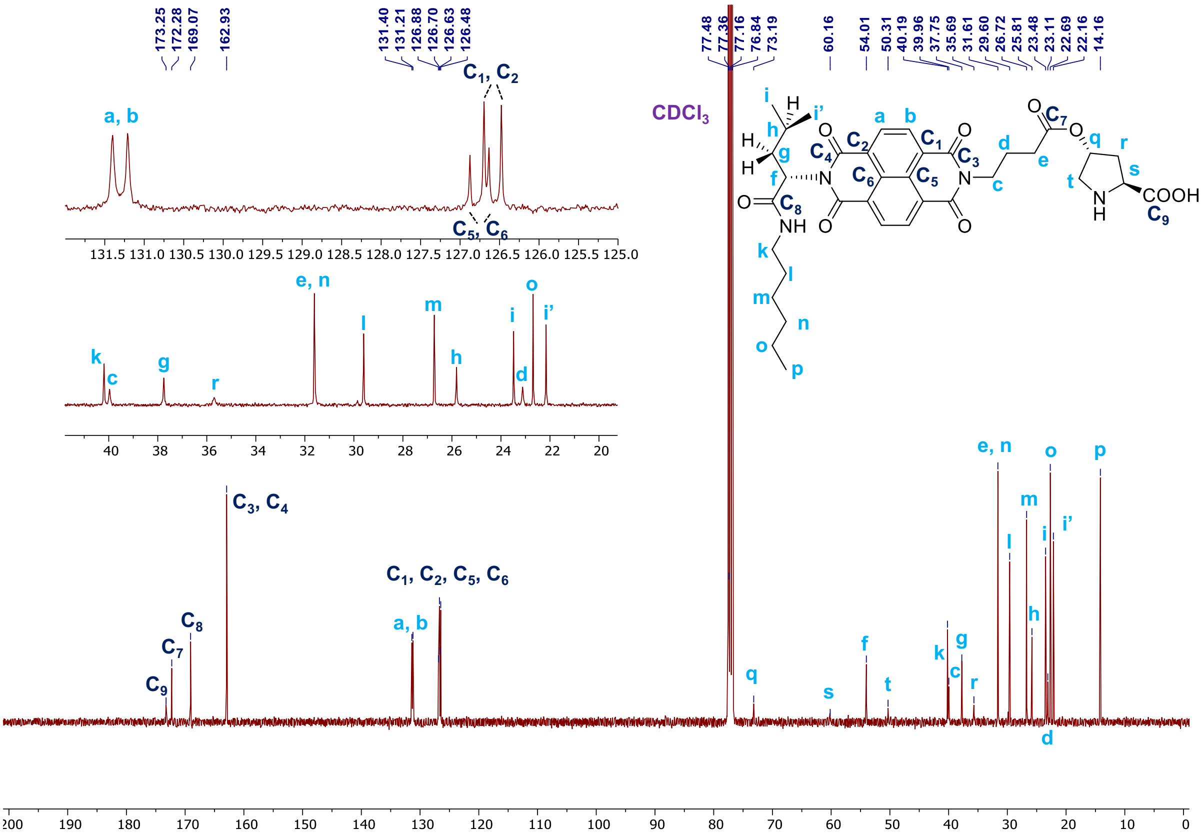

Figure S59. 101 MHz ^{13}C NMR spectrum of **4** in $CDCl_3$.
